# TargetMITO: a rule-based model for generating functional synthetic mitochondrial targeting sequences in yeast

**DOI:** 10.1093/nar/gkag986

**Published:** 2026-10-21

**Authors:** Kewin Gombeau, Raymond Wan, Joshua S James, Déborah Tribouillard-Tanvier, Yizhi Cai

**Affiliations:** Manchester Institute of Biotechnology, University of Manchester, 131 Princess Street, M1 7DN, Manchester, United Kingdom; Manchester Institute of Biotechnology, University of Manchester, 131 Princess Street, M1 7DN, Manchester, United Kingdom; Manchester Institute of Biotechnology, University of Manchester, 131 Princess Street, M1 7DN, Manchester, United Kingdom; CNRS, IBGC, UMR 5095, University of Bordeaux, F-33000 Bordeaux, France; Manchester Institute of Biotechnology, University of Manchester, 131 Princess Street, M1 7DN, Manchester, United Kingdom

## Abstract

Mitochondria are essential organelles containing their own genomes, encoding a few proteins essential for energy production. Most of the mitochondrial proteins are nucleus-encoded, translated as precursors in the cytosol, with a large fraction of these precursors properly addressed by an N-terminal mitochondrial targeting sequence (MTS). These MTS share common features but no consensus sequence can explain their functionality nor the precursors-specific determinants of mitochondrial import. To help understand this mechanism, we created a simple computational model to generate functional synthetic MTS while maintaining a tight control on the design parameters. Using the budding yeast, we demonstrated the presence of precursors-specific import capabilities in addressing artificially nucleus-relocated OXPHOS proteins. Our research confirms the existence of cargo-dependent functional compatibilities and takes us one step closer towards improving gene therapy-based treatment of mitochondrial diseases.

## Graphical Abstract

**Graphical Abstract:**
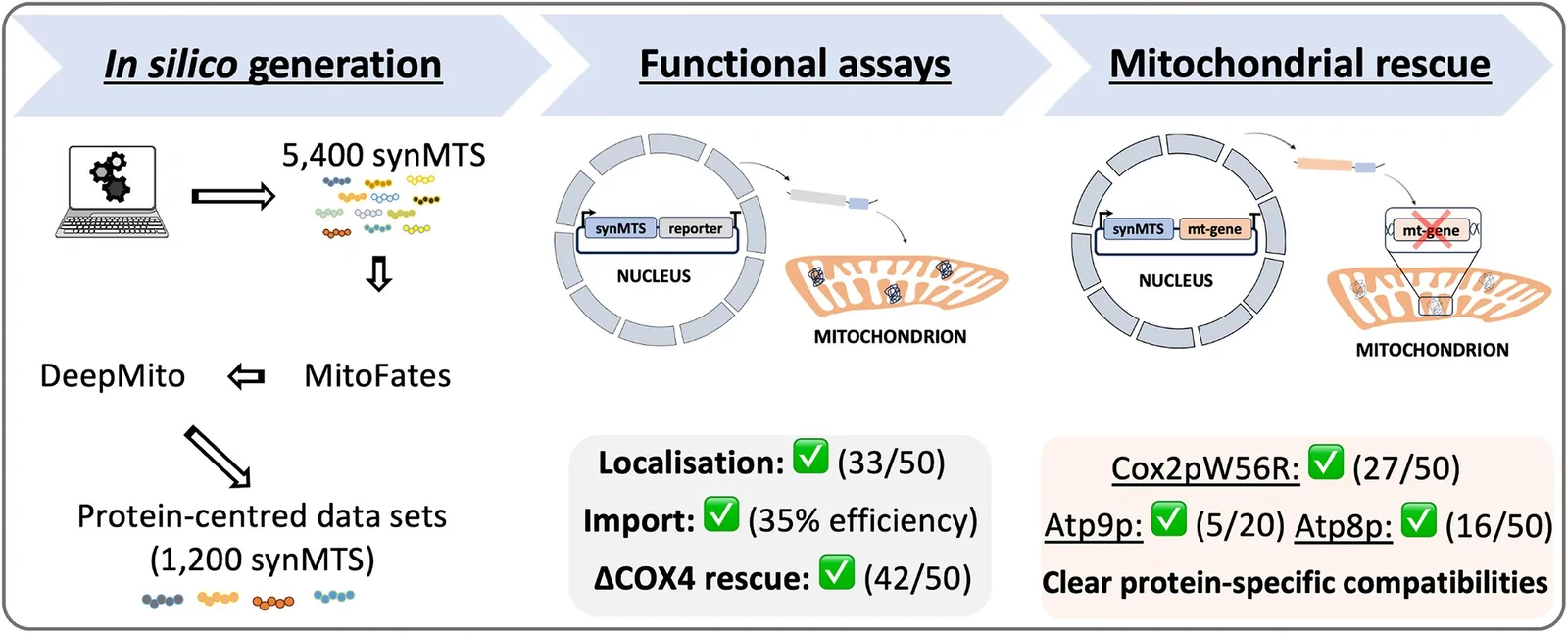

## Introduction

Mitochondria are crucial eukaryotic organelles hosting multiple essential biological processes encompassing the biosynthesis of branched chained amino acids and iron-sulphur clusters, calcium homeostasis, the tricarboxylic acid cycle (TCA cycle), lipids metabolism, regulation of apoptosis, and ATP production through the oxidative phosphorylation system (OXPHOS system) [1]. While most of these functions are encoded in genomic DNA, the OXPHOS system of most organisms presents a unique feature: a dual-genetic origin, from both the nuclear and mitochondrial genome [mitochondrial DNA (mtDNA)] [2]. Interestingly, on the one hand, this OXPHOS system involves about 100 different protein species among which a small fraction (13 in human and 7 in budding yeast) remains encoded in the mtDNA, along with a set of transfer RNAs and ribosomal RNAs essential for the intraorganellar translation of mitochondrial proteins [2]. On the other hand, the nucleus-encoded mitochondrial proteins are synthesized as protein precursors in the cytosol prior to being targeted to mitochondria. A large fraction of these precursors contains at their N-terminus a cleavable positively charged amphipathic presequence signal (with a length varying between 10 and 120 amino acids), often forming an alpha-helix, and referred to as the mitochondrial targeting sequence (MTS) [3]. These MTS play a crucial role in ensuring an efficient mitochondrial import of the protein precursor by guiding it towards its corresponding mitochondrial sub-compartment where it is matured through the action of mitochondrial peptidases excising the MTS [3].

For more than 40 years, researchers have explored the determinants of mitochondrial import by trying to identify a potential amino acid pattern explaining the functionality of these MTS [4–8]. The first efficient artificial MTS were generated in 1986 using different ratios of only three amino acids: arginine, serine, and leucine. They were able to address Cox4p to its correct mitochondrial location [5]. Two years later, in a more extensive study, the same group confirmed the central role played by amphiphilicity in the functionality of their artificial MTS while the helical structure appeared dispensable [8]. In 1989, Lemire and collaborators [7] generated 89 10 amino acids long artificial MTS (starting with a methionine and followed by 9 random amino acids) and tested them for their ability to address Cox4p to mitochondria. Among these random peptides, the quarter that was functional had the potential to form amphiphilic alpha-helixes, was enriched in arginine/leucine, and depleted in aspartate/glutamate (acidic residues) and proline (creating helix-breaking). These results demonstrated that rather than a specific amino acid consensus sequence, the amphipathic structure is central in explaining the functionality of the presequences. Indeed, their hydrophilic and positively charged side is crucial to promote recruitment and import through the translocase complexes (TOM and TIM for the outer and inner membrane, respectively), while their hydrophobic side enables smooth gliding through the lipid membrane bilayer [3, 9, 10]. Recently, a convolutional neural network was developed to generate artificial presequences derived from data sets of naturally occurring MTS [4]. The authors then tested four sets of eight randomly sampled presequences for their ability to address a fluorescent reporter to mitochondria in *Homo sapiens* (100% functional), *Rhodotorula toruloides* (75% functional), *Nicotiana benthamiana* (75% functional), and *Saccharomyces cerevisiae* (50% functional). In keeping in line with earlier works, the physicochemical properties of the newly generated peptides matched those of natural presequences: amphipathic peptides enriched in arginine with an alpha-helical structure.

The aforementioned studies confirmed that artificial sequences mimicking natural ones can be used to target simple soluble proteins to mitochondria. However, for more complex cargos such as nucleus-relocated mitochondrial proteins, MTS inter-compatibility does not always exist. Also referred to as allotopic expression, the artificial nuclear relocation of mitochondrial genes is widely used to both understand the forces responsible for the retention of mitochondrial protein-coding genes in the mtDNA as well as to create gene therapy-based treatments for mtDNA-linked diseases [11]. This method requires: (i) recoding the mitochondrial gene to match the nuclear genetic code; (ii) attaching to its N-terminus an MTS to properly address the cytosol-synthesized protein to the mitochondrial inner membrane; and (iii) adjusting the physicochemical properties of the relocated protein to prevent cytosolic aggregation and/or mislocalization [12–17]. In our recent study on *COX2-W56R* nuclear relocation in yeast, we demonstrated that despite identifying new functional mutations improving Cox2p-W56R import and sorting, a significant fraction of the allotopic protein was not imported into the organelle [18]. Importantly, while looking for potential endogenous MTS capable of efficiently addressing the protein to mitochondria, our findings highlighted that *OXA1* MTS was the most efficient, even when compared to endogenous MTS addressing nucleus-encoded Complex IV proteins (i.e *COX6, COX11*, or *COX15* MTS) [18]. This is in line with previously reported results [19] where 20 different individual MTS were tested for their ability to target nine different allotopic proteins in human cells. Those results pointed out a low efficiency of the endogenous sequences, highlighting the lack of effective MTS inter-compatibility between mitochondrial proteins. This is probably due to both the hydrophobic nature of these transmembrane proteins which impacts their mitochondrial import and the possibility that native MTS have co-evolved with the protein they are attached to, ensuring efficient co-translational import and sorting into mitochondria. As such, in addition to the amphipathic structure conferring MTS their function, additional yet-to-be-discovered rules appear to be governing the import of mitochondrial proteins. This hypothesis was recently corroborated by Rödl and collaborators who proposed that MTS contain a protein-specific priority code to efficiently address their cargo to its mitochondrial sub-compartment [20].

Elucidating the mechanism of mitochondrial protein import is crucial to aid in the development of new treatments for mtDNA-linked diseases. Indeed, these diseases are among the most common genetic disorders in humans [21], and to date, no effective treatment has been discovered. Multiple routes are being explored, among which gene therapy replacement, involving the allotopic expression of a healthy copy of a mitochondrial gene, is a promising candidate [22–24]. To identify the passenger protein-specific import determinants, a large set of *in vivo* functionally validated presequences (MTS) is required, and this is currently limited by both the small size of public data sets and the poor inter-compatibility of MTS. To fill this gap and help understand the addressing of artificially nucleus-relocated mitochondrial proteins, we have created TargetMITO, a simple model offering total control over the design parameters to generate functional synthetic presequences. The classic approaches using aggregated analyses have been useful in identifying the most commonly shared features between MTS (i.e. positively charged arginine-rich and amphipathic peptides with an *α*-helical structure carrying a mitochondrial peptidase cleavage site), but a direct consequence of this methodology is the dilution of the precursors-specific MTS composition that explains the addressing to a specific mitochondrial sub-compartment. Instead of deriving peptides from natural data sets of MTS (either pooled from a single organism or gathered from multiple life kingdoms), we defined four rules based on both the known physical-chemical properties conferring MTS functionality and empirical observations of MTS composition: (i) a sequence with a length between 40 and 120 amino acids starting with a methionine; (ii) the presence of a mitochondrial peptidase cleavage site at the end of the sequence; (iii) the presence of a repetitive pattern of arginine and lysine residues to promote amphiphilicity; and (iv) a doubled occurrence of arginine/lysine every 30–40 amino acids.

Using the aforementioned rules, we created nine different methods to generate synMTS and used MitoFates [25] to confirm *in silico* that those capturing all of the known MTS characteristics, Methods 4 and 7, were the most promising. The synMTS generated by these two methods were then merged into a single data set (1200 sequences), and using DeepMito [26], we benchmarked our synMTS *in silico* against commonly used MTS in artificial nuclear relocation experiments in yeast (the two gold-standards: *OXA1* MTS and *SU9* MTS). The presequences were appended to three mitochondrial proteins sequence: Cox2p-W56R, Atp9p, and Atp8p, and probability scores of mitochondrial targeting were obtained using DeepMito generating three sets of allotopic protein-centred synMTS rankings. We focused on these proteins as they represent stringent test cases since hydrophobic mtDNA-encoded membrane proteins are particularly prone to cytosolic aggregation, mistargeting and inefficient mitochondrial import when expressed from the nucleus. Additionally, Cox2p and Atp8p are especially relevant from a translational perspective because their human homologues are encoded by the mitochondrial genome and are therefore representative of clinically relevant targets for eventual future gene therapy-based therapeutic strategies. By contrast, Atp9p is already nucleus-encoded in mammals, but provides an unusually restrictive yeast benchmark because it is a small, highly hydrophobic ATP synthase subunit whose functional rescue requires an extended targeting architecture, and particularly the use of two MTS copies. Thus, these three cargos offer clinical relevance and increasing levels of import difficulty, allowing us to test whether synthetic presequences can support challenging mitochondrial targeting of inner membrane proteins.

Across the three sets of ranked presequences, 20 synMTS were selected (50 unique sequences in total), synthesized, and functionality was assessed *in vivo*. We applied various assays starting with a fluorescence-based localization test to confirm the ability of the synMTS to efficiently target mitochondria (66% functional). Using the IQ-Compete assay [20], we measured the import properties of our synthetic presequences (100% functional). Next, we tested the ability of these synMTS to address the nucleus-encoded Cox4p by virtue of restoring the respiratory growth of the *COX4* deletion strain (84% functional). After confirming the functionality of the 50 synMTS sampled, we tested their ability to target to mitochondria the three allotopically expressed proteins (Cox2p-W56R, Atp9p, and Atp8p). The first screening used the original protein-centred sub-groups of 20 synMTS. We evaluated the restoration of the respiratory growth of the three different deletion strains, and measured the oxygen consumption rates (OCR) in whole cells for a subset of the best performers. This first set of results demonstrated that our model could generate functional presequences able to efficiently address complex allotopic cargos to mitochondria. As only five of the twenty synMTS tested were compatible with Atp9p, we decided to focus on Cox2pW56R and Atp8p and extended the characterization to the entire set of 50 synMTS. We identified clear instances of passenger protein-specific functionality, emphasizing the potential our model offers to shed light on new rules governing mitochondrial import of nucleus-relocated OXPHOS proteins. By extending the characterization of these synMTS into human cell models, TargetMITO could improve the development of gene therapy-based treatment of primary mtDNA diseases affecting protein-coding genes in humans.

## Materials and methods

### synMTS generation

The design of our synthetic MTS involved maintaining a repetitive pattern of hydrophilic residues (arginine and lysine) while filling empty intervals with randomly chosen residues excluding the hydrophilic ones to randomly generate amphipathic presequences. We begin by defining some notation in order to explain the properties of our synMTS. We represent the set of all 20 canonical amino acids as $\mathcal {A}$. We segment $\mathcal {A}$ into separate sets as follows. The set $\mathcal {H}_1$ includes the two strongly hydrophilic and positively charged amino acids [lysine (K) and arginine (R)], while $\mathcal {H}_2$ includes the next two strongly hydrophilic negatively charged amino acids [aspartic acid (D) and glutamic acid (E)]. Meanwhile, $\mathcal {H}$ is defined as the union of these two sets. In other words, $\mathcal {H}_1 \in \lbrace \textrm {K}, \textrm {R}\rbrace$; $\mathcal {H}_2 \in \lbrace \textrm {D}, \textrm {E}\rbrace$; and $\mathcal {H} = \mathcal {H}_1 \cup \mathcal {H}_2$. Finally, the remaining 16 amino acids are placed in the set $\mathcal {O}$.

Using these sets as building blocks, we incrementally apply design rules to guide the generation of our synMTS data sets through nine different methods whose parameters are summarized in Table 1. Each column in this table indicates a feature, which from left-to-right, is associated with an increased stringency in the applied design. Method 1 simply requires the presequence to start with a methionine (M). The remainder of the presequence is calculated randomly from all of the amino acids using a uniform distribution, so that the total presequence is between 40 and 120 amino acids in length.

**Table 1.** Summary of the nine methods considered by this work for the generation of the synMTS data set

	Random pattern			Fixed pattern of			
	Begin	End with	Charge	hydrophilic residues			Doubled
Method	with M	R$\mathcal {A}$ / R$\mathcal {A}$Y	*≥* 8	$\mathcal {H}_1$	$\mathcal {H}_2$	$\mathcal {H}$	$\mathcal {H}_1$
1	✓						
2	✓	✓					
3	✓	✓	✓				
4	✓	✓		✓			
5	✓	✓			✓		
6	✓	✓				✓	
7	✓	✓		✓			✓
8	✓	✓			✓		✓
9	✓	✓				✓	✓

Each column represents a property that the method has, as indicated by ✓. In this table, $\mathcal {H}_1$ = lysine and arginine, $\mathcal {H}_2$ = aspartic acid and glutamic acid, $\mathcal {H}$ = $\mathcal {H}_1 \cup \mathcal {H}_2$, and $\mathcal {A}$ = all the amino acids. M = methionine, R = arginine, Y = tyrosine.

In addition to the starting methionine requirement, Method 2 stipulates that each MTS must also include a known mitochondrial peptidase cleavage site, corresponding to either an arginine at the *-2* position from the C-terminal (R-2) or arginine at the *-3* position followed by a tyrosine at the *-1* position (R-3 and Y-1) [27].

Building on Method 2, Method 3 aims to increase the net positive charge, a crucial feature of MTS. We achieve this by randomly permuting the amino acids in favour of the two $\mathcal {H}_1$ residues until the net positive charge of the entire presequence is eight or higher, to match the minimal positive net charge of *OXA1* MTS.

The remaining six methods include hydrophilic residues (i.e., $\mathcal {H}_1$, $\mathcal {H}_2$, or $\mathcal {H}$) at intervals of every 3–5 amino acids so as to mimic the observed repetitive pattern seen in the reference MTS (Fig. 1A and B). In between these hydrophilic residues are amino acids randomly taken from $\mathcal {O}$. Moreover, we observed in the reference MTS hydrophilicity patterns few instances of hydrophilic residues that were doubled. Thus, for the last three methods, we decided to explore the influence of this doubled hydrophilic positions by inserting an amino acid from $\mathcal {H}_1$ in the first 30–40 amino acids. For synMTS longer than 40 amino acids, these intervals are used: *[30, 45]; [60, 75]; [90, 110]*. A summary of the different methods deployed in this study is provided in Table 1. An alternative but more precise description of these nine methods is provided in the supplementary materials as a table of regular expressions (see Supplementary Table S8).

**Figure 1.**
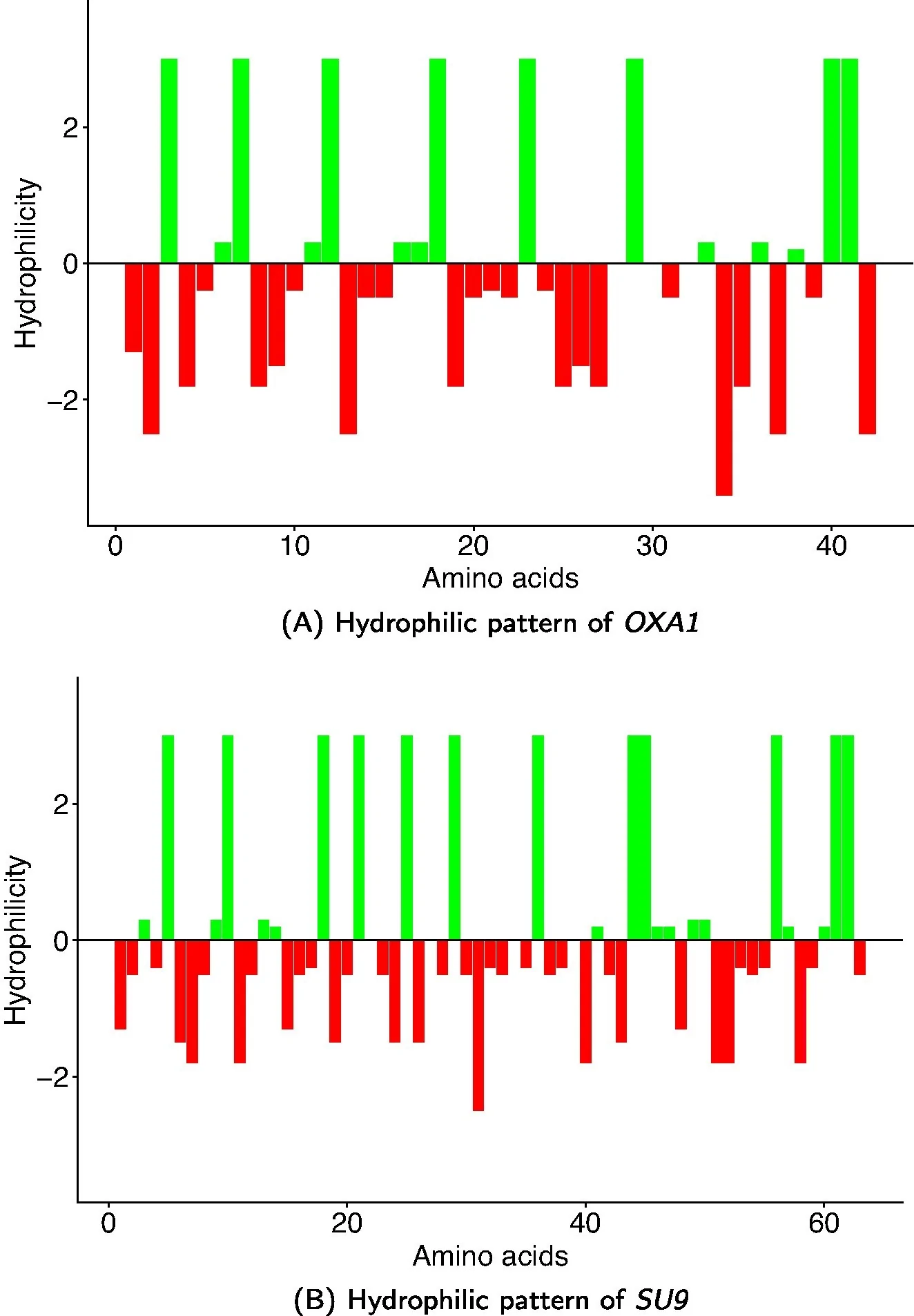
Hydrophilic patterns of the reference (**A**) *OXA1* and (**B**) *SU9* MTS used in artificial nuclear relocation experiments. The large green bars represent the hydrophilicity value of either arginine or lysine.

These methods were employed for the generation of synMTS *in silico*. More precisely, for each of these nine methods, three independent replicates of 200 synthetic MTS were produced (600 presequences per method), yielding 5400 synMTS in total (as depicted in Supplementary Fig. S1A).

Hydrophylic patterns in this work were calculated using the amino acid hydrophilicity scale of Hopp and Woods [28] (see also Supplementary Table S9).

### Homology search with BLASTP

Homology search was performed using BLASTP and the RefSeq protein database downloaded on 28 August 2025 from https://ftp.ncbi.nlm.nih.gov/blast/db/ [29].

BLASTP version 2.16.0+ was set up by installing the Ubuntu package “ncbi-blast+” version 2.16.0+ds-7. The synMTS data set was queried using the following command:

time blastp -query. /query.fasta -db \

refseq_protein -out results.tab -evalue \

1e-5 -outfmt 7 -num_threads 24

Here, an E-value cut-off of $1 \times 10^{-5}$ and 24 threads of execution was employed.

### Benchmarking the synMTS with MitoFates

The MitoFates web server [25] is a web-based prediction software that analyses the N-terminus region of the submitted protein sequence and returns a presequence probability score indicating the likelihood of the presence of an MTS. Since MitoFates had been trained on *S. cerevisiae* data sets of experimentally confirmed mitochondrial proteins, we considered it as an adequate tool to benchmark the different methods used to generate our synthetic MTS.

Our analysis with MitoFates was performed using the web server at the following address: https://mitf.cbrc.pj.aist.go.jp/MitoFates/cgi-bin/top.cgi (last accessed 1 September 2026).

### Exploring the “synMTS-passenger protein” synergy with DeepMito

The synergy between MTS and the appended protein can be evaluated using the DeepMito web server [26]. DeepMito provides predictions on: (i) whether a submitted protein, analysed in its entirety (MTS + passenger protein), is localizing to mitochondria; and (ii) the likelihood of mitochondrial sub-localization using artificial neural networks trained on data sets of mitochondrial proteins (covering multiple kingdoms of living organisms) whose sub-localization have been experimentally validated. For the former, DeepMito leverages BaCelLo and TPpred3 to return a “Yes/No” prediction of mitochondrial affiliation [30–32]. For the latter, DeepMito provides a score from 0 to 1 to represent the likelihood.

Analysis with DeepMito was performed using the web server at the following address: https://busca.biocomp.unibo.it/deepmito/ (last accessed 1 September 2026).

### Yeast culture and transformation

We used the previously described biolistic transformation procedure and the *ARG8^m^* marker to generate the different mitochondrial deletions [33]. Briefly, the different deletion cassettes were delivered in the rho0 DFS160 yeast strain which was then crossed with the wild type strain YCy5051 (referred to as WT) to create the different single deletion strains [either YCy6163 (referred to as *Δ**atp9::ARG8^m^*), or YCy6976 (referred to as *Δ**atp8::ARG8^m^*)]. All liquid precultures were performed in liquid synthetic media with corresponding dropouts, starting from fresh transformants and incubated without shaking. We performed the spot test assays on rich media (1% yeast extract and 2% peptone) supplemented with either 2% glucose or 2% glycerol. We spotted, for each strain, a 10-fold serial dilution, starting from a concentration of 1 OD_600nm_ unit/ml and growing the cells at 30$^\circ$C for the indicated time. A list of the yeast strains used and generated in this study is provided in Supplementary Table S1.

### Plasmids assembly strategy

In the present study, the different transcriptional units were assembled using the Golden Gate-based method called YeastFab [34], with modified overhangs to enable the incorporation of one or two MTS between a given promoter and a coding gene, as previously reported [18]. The Golden Gate reactions were transformed in TOP10 *Escherichia coli* competent cells and the expression vectors purified using the E.Z.N.A. plasmid miniprep kit (Omega Bio-tek, Norcross, GA, USA). Then, these expression vectors were transformed in yeast using the classic LiOAc-based transformation protocol isolating the correct transformants on synthetic solid media with corresponding amino acid dropouts. Lists of the primers (Supplementary Table S2) and plasmids (Supplementary Table S3) are provided in the supplementary files.

### Fluorescence-based procedures

Using fluorescence microscopy, we appraised the capability of our synthesized synMTS to address a Cherry fluorescent protein to mitochondria. To achieve this, we imaged cells during their exponential phase of growth using an inverted epifluorescence microscope (Nikon Eclipse TE2000U) equipped with an 100*×* immersion objective and a standard FITC filter. As a positive control, mitochondria were stained using MitoTracker™ Red CMXRos (Thermo Fischer, M7512). The results are presented in Fig. 4.

To quantitatively measure the import properties conferred by our synMTS, we used the IQ-Compete assay [20], adapting the procedure to make it compatible with our YeastFab assembly method [34]. Briefly, the Tobacco Etch virus protease (uTev) was combined with the different synMTS and co-expressed, along with the GFP-quencher-degron, on two different low-copy vectors (see Supplementary Table S3 for a list of plasmids generated). The GFP signal obtained under each condition was normalized to the cell density in each well, and transformed into a relative percentage of GFP import by normalizing the signal measured in both the condition where uTev is cytosolic (cyt.uTEV, no MTS added simulating 0% import) and the condition without uTev (w.o.uTEV, simulating 100% of import). The transformants were grown until saturation without shaking for 24 h, and then, the precultures were transferred into synthetic media with the corresponding dropouts supplemented with 2% glucose and the fluorescence monitored for 24 h using a Cytation 5 plate reader (Agilent). The GFP fluorescence signal was used when cells were in their exponential phase of growth. The results are presented in Fig. 5 and the corresponding data is presented in Supplementary Table S6.

### Measurement of whole cells OCR

We quantified the ability of the selected allotopic expression systems to complement the deletion of their corresponding mitochondrial gene (either *COX2-W56R, ATP9*, or *ATP8*) by measuring the rates of oxygen consumption (OCR, using a Clark electrode from Hansatech, UK). For each of the different allotopic strains, freshly obtained transformants were inoculated in static synthetic liquid media (with dropouts corresponding to the auxotrophy markers used and supplemented with 2% glucose) and grown overnight at 30$^\circ$C. The next day, the cultures were transferred to 6 ml of rich medium supplemented with 2% glycerol (YPGly 2%) at a starting OD_600nm_ of 0.25/ml and grown overnight at 30$^\circ$C with shaking. Finally, the cells were harvested during their exponential growth phase to measure the OCR on whole-cells. The OCR is expressed as nmol of O_2_ consumed per min per optical density (OD) unit. The raw data of OCR measurements on whole cells are available in Supplementary Table S7.

### Bioinformatics analyses

Additional bioinformatics analyses were performed by hmoment and Streme. Amphipathicity of a sequence was measured using the hmoment software from the EMBOSS software suite (version 6.6.0.0) [35] with the procedures by Eisenberg and collaborators [36, 37]. The hmoment software was applied with default options (sliding window of 10 amino acids, *α*-helix angle of 100 degrees, and *β*-sheet angle of 160 degrees). Three values of interest were considered: the maximal hydrophobic moment across the entire sequence (*μ*Hmax), the mean value of the Top10% hydrophobic moments (*μ*Htop10%), and the mean hydrophobic moment for the entire sequence (*μ*Hmean).

Streme version 5.5.9 of the Meme Suite was used to discover ungapped motifs [38]. Streme takes as inputs a positive set of protein sequences and a negative set of protein sequences. Its aim is to identify motifs in the positive set that are absent in the negative set. The same default options as the web site (https://meme-suite.org/meme/) were used, with the following exceptions: (i) the minimum width was set at 5, since that is the maximum length of the hydrophilic pattern that we have previously observed and (ii) the synMTS were aligned to the left since they are protein sequences.

We applied Streme in two different ways. First, we used the protein sequences as is. Next, because we hypothesized that mitochondrial targeting signals are based on the properties of the amino acids themselves, we grouped amino acids into the 7 categories of Supplementary Table S10. For example, lysine (K), arginine (R), and histidine (H) were all changed to the group “H”, which now represents “hydrophilic polar basic (positive charge)”.

Other than the externally available web applications MitoFates and DeepMito, all other computational experiments were performed locally on a Intel^®^ Core^™^ i9-14900K CPU with 32 GiB RAM running Ubuntu 26.04. Computational experiments included the execution of hmoment and Streme, as well as custom-made Perl scripts (for Perl version 5.32.1) and R scripts (for R version 4.4.2). All of these programs were wrapped within a series of Snakemake version 8.26.0 workflows [39].

### Statistical analyses on biological data

All quantitative biological experiments were conducted with three independent biological replicates per condition. Prior to group comparisons, data were assessed for normality and homogeneity of variance using the Shapiro–Wilk and Levene tests (*α* = 0.05). When both assumptions were met, a parametric one-way analysis of variance (ANOVA) with Dunnett’s *post-hoc* correction was applied; otherwise, a Kruskal–Wallis test followed by Mann–Whitney comparisons (*α* = 0.05) was used. Statistical analyses were performed in RStudio (version 4.2.3), and reported *P*-values are corrected values.

### Software and data availability

Source code for generating and evaluating the synMTS data sets is publicly available through this GitHub repository: https://github.com/the-cai-lab/synMTS.

The generated synMTS data set of 5400 synthetic MTS sequences and our results from submitting these sequences to the MitoFates and DeepMito web servers are also included within this GitHub repository, as are the scripts for applying and post-processing the results from hmoment and Streme.

## Results

### Design and validation of novel synthetic MTS

As previously mentioned, the design of our synMTS relied on four simple rules derived from both the known physical chemical properties conferring MTS functionality as well as empirical observations. Particularly, we carefully examined the physicochemical properties of the *OXA1* and *SU9* MTS which provided the best results in addressing Cox2p-W56R and Atp8p/Atp9p to mitochondria, respectively, in previous artificial nuclear relocation experiments in yeast [13, 16, 18, 40]. The hydrophylic patterns of *OXA1* MTS (8 positive net charges) and the *SU9* MTS (12 positive net charges) are shown in Fig. 1A and B. These two examples illustrate the features that are essential in determining the functionality of MTS: (i) a total positive net charge; (ii) an amphipathic structure; and (iii) a frequent occurrence of arginine and lysine residues. In addition, we observed a doubled occurrence of positively charged residues every 30-40 amino acids which should also be considered when designing our synthetic presequences.

Then, we generated synMTS sequences with lengths (Supplementary Fig. S2) and total net charges (Supplementary Fig. S3) that were consistent with the set of rules imposed across the different methods. A homology search using BLASTP on the entire data set was performed, with no hits returned, confirming that the generated sequences are entirely new-to-nature.

We appended five proteins to these presequences among which three are mitochondrial proteins localizing to the inner mitochondrial membrane (Cox2p-W56R, Atp8p, and Atp9p) that have been previously artificially expressed from the nucleus using either *OXA1* or *SU9* MTS [13, 16, 18, 40]. The fourth sequence is a nuclear transcription factor (Hac1p), which will act as a negative control, confirming the absence of any passenger protein influence on MitoFates predictions. The fifth sequence (Mmf1p) is a matrix-localized protein involved in mtDNA maintenance and amino acid metabolism, which will act as a positive control.

After applying MitoFates to these synMTS, we observed variations in the presequence probability scores across the different methods according to the stringency of the applied rules (see Fig. 2A–E). The data set of random sequences starting with a methionine (Method 1) returned low probability scores, which was not improved by the addition of known mitochondrial cleavage sites (Method 2). While a slight improvement was observed when the synthetic MTS had to carry a minimum of eight positive net charges (Method 3), the most substantial enhancement was achieved by enforcing a regular pattern of positively charged residues (Methods 4 and 7). Conversely, incorporating a mixture of hydrophilic charged residues (Methods 6 and 9) led to a significant reduction in prediction scores, while designs utilizing only negatively charged residues completely eliminated any positive predictions (Methods 5 and 8), confirming the need to only use positively charged hydrophilic residues.

**Figure 2.**
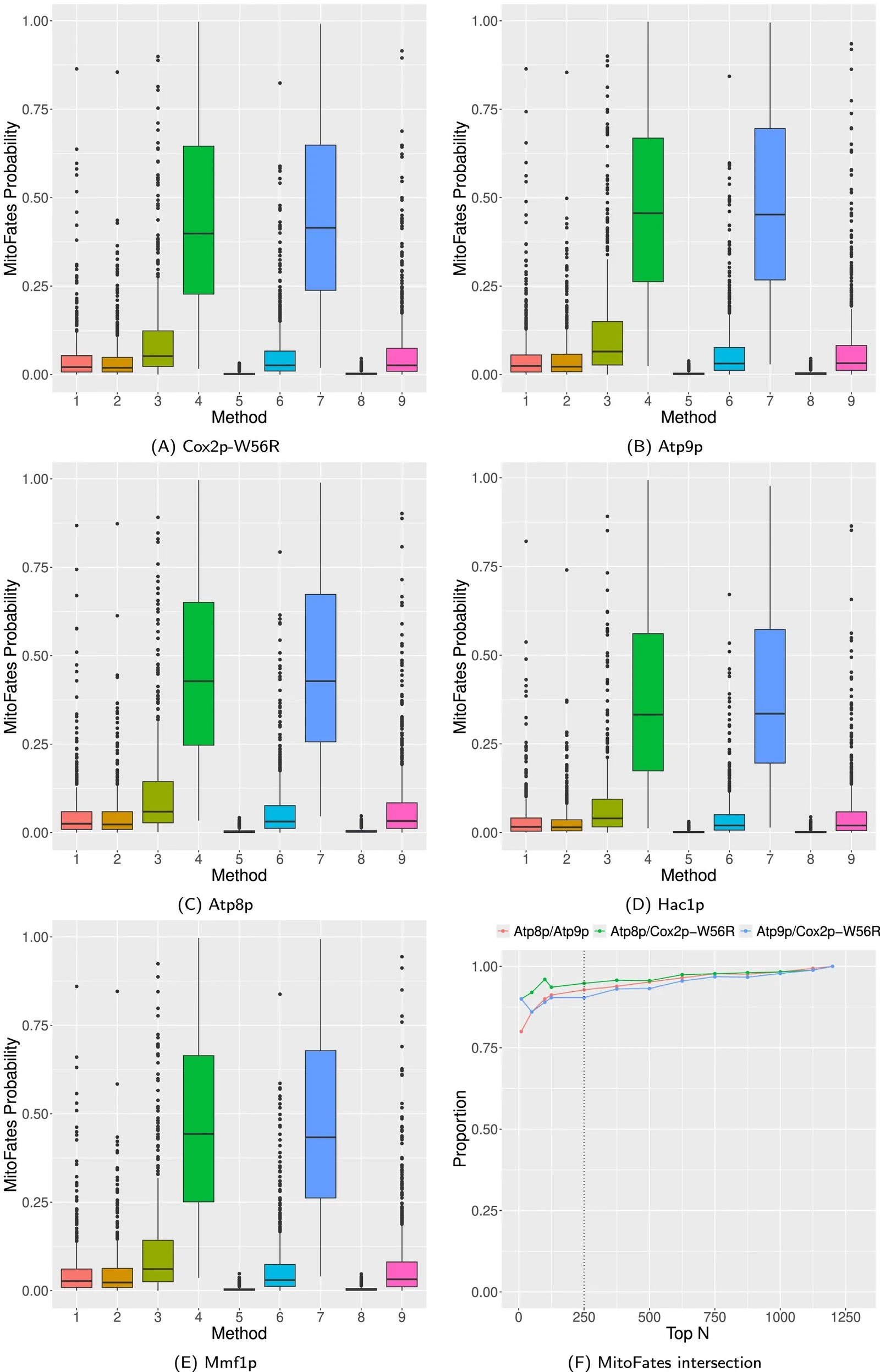
Summary of findings from MitoFates. Box plots of the distribution of the MitoFates presequence probability scores obtained for each method, separated by passenger protein: (**A**) Cox2p-W56R, (**B**) Atp9p, (**C**) Atp8p, (**D**) Mmf1p, and (**E**) Hac1p. (**F**) Intersection of the top N MitoFates presequence probability scores for the merged data sets from Methods 4 and 7 with either Cox2p-W56R, Atp9p, or Atp8p appended. The dotted black vertical line indicates the proportion of overlapping scores when only the top 250 synMTS-passenger proteins are considered.

Given the well-known central role of positive net charges in promoting mitochondrial import, we tested whether the total positive net charge count was the main driver of the high MitoFates prediction scores. Here, we focus on Methods 4 and 7, since they offer the highest scores. To achieve this, we replaced in these synMTS all instances of arginine by a lysine, except those involved in constituting the mitochondrial peptidase cleavage site (R-2 or R-3 + Y-1), and re-submitted the sequences to MitoFates. We obtained new prediction scores, and plotted the scores with substitutions versus those previously obtained when using arginine (Supplementary Figs S4–S8). We observed that using lysine only resulted in a significant drop in the MitoFates prediction scores, despite maintaining the same total positive net charge count. This is consistent with the central role given to the presence of arginine residues in the MitoFates prediction scores, as reported by Fukasawa and collaborators: “the best feature is the score of MPP cleavage site (F-score = 0.250), and the next best four are the composition of Arg in the N-terminal 30 residues (0.217), the total hexamer motif score (0.159), the dipeptide composition of Leu-Arg (0.126), and the positively charged amphiphilicity (PA) score (0.126), respectively.” [25].

To appraise the dependency of the calculated MitoFates probability scores on the protein sequence appended to the different MTS, we included the negative control Hac1p (a nuclear transcription factor) and the positive control Mmf1p. The obtained results confirmed that MitoFates predictions are robustly consistent across the different conditions regardless of the appended protein.

To further confirm these findings, we merged the high scoring data sets from Methods 4 and 7 for each protein, as depicted in Supplementary Fig. S1B. We then sorted the scores from highest to lowest and took the top N scores, for various values of N. Finally, we intersected the lists for every possible pair of mitochondrial proteins (Fig. 2F). For example, for the pair Atp8p and Atp9p, 92.8% of the top 250 scores for these two passenger proteins overlap (the dotted black vertical line in this figure). As this graph confirms, the results are highly similar across the different conditions and rather insensitive to the appended protein.

We measured different metrics describing the amphipathicity of the synthetic presequences. Across the nine methods, hydrophobic-moment profiling revealed that Methods 4 and 7 generated the most strongly amphipathic synthetic presequences with higher *μ*Hmax (Supplementary Fig. S9), *μ*Htop10% (Supplementary Fig. S10), and *μ*Hmean (Supplementary Fig. S11). We tested whether these parameters correlated with the obtained MitoFates prediction scores, but only identified weak positive associations (Supplementary Figs S12–S26). This result is consistent with their markedly positive net charges and with their superior MitoFates scores, indicating that these methods most efficiently recreate the canonical physical-chemical features of mitochondrial presequences.

### Cargo-specific synMTS rankings based on predicted sub-mitochondrial localization scores

Next, we aimed to explore the synergy between our synMTS and the appended proteins with the merged data sets created from just Methods 4 and 7. These data sets were submitted to the DeepMito web server for assessment [26]. As shown in Fig. 3A and B, correct mitochondrial localization was confirmed for >90% of the synMTS-mitochondrial proteins precursors, as well as for the positive control, but not for the negative control.

**Figure 3.**
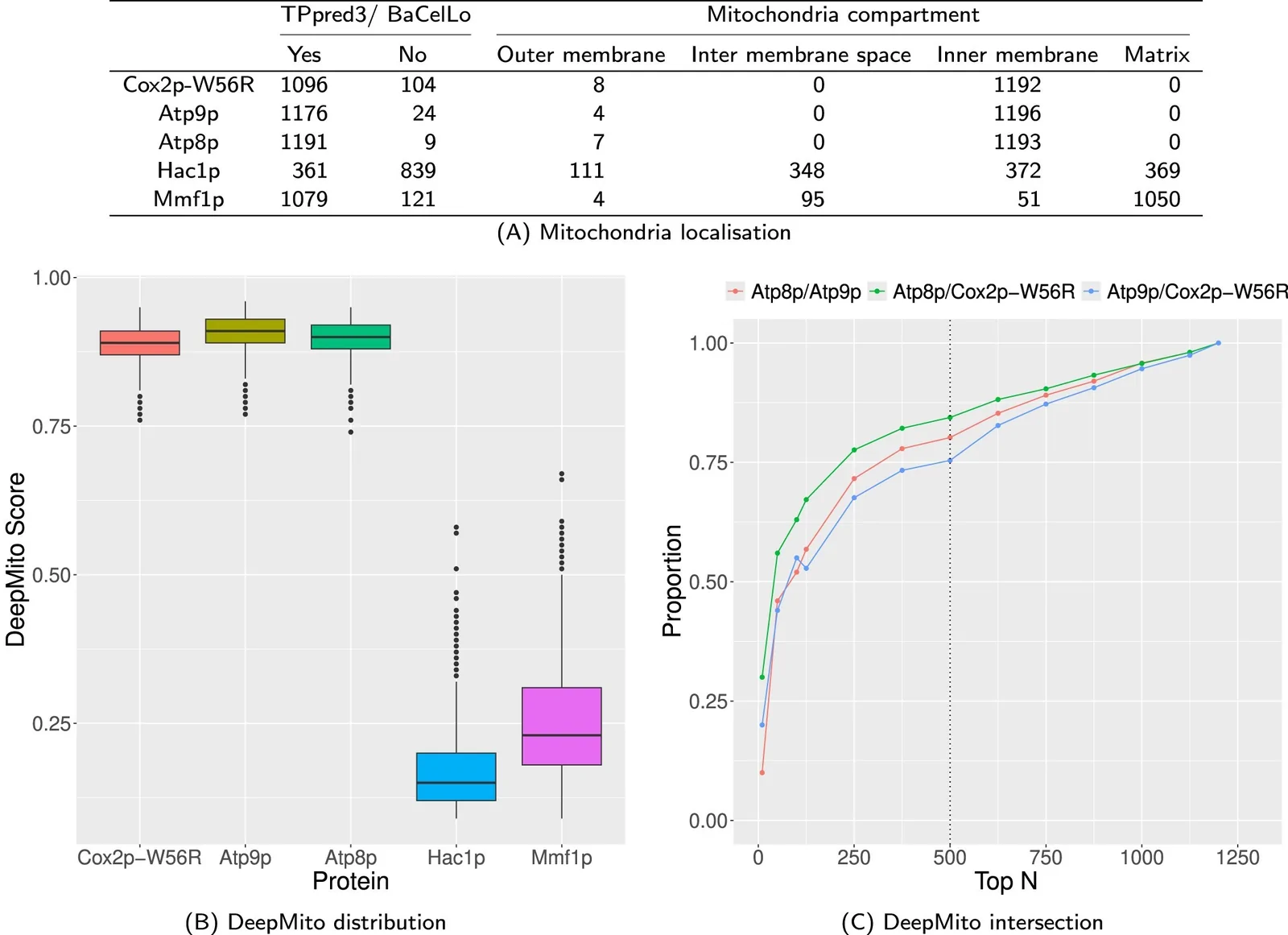
Summary of findings from DeepMito, after focussing on just Methods 4 and 7. (**A**) Mitochondria localization, as reported by TPpred3 and BaCelLo as a “Yes” or a “No”. Regarding the sub-compartment localization, DeepMito calculates a probability score for each compartment and returns the highest score and the corresponding sub-compartment. (**B**) Box plots presenting the DeepMito sub-compartment localization scores for each of the five passenger proteins. (**C**) Pairwise comparison of synMTS-passenger protein pairs, focussing on the three allotopic proteins. The graph shows the proportion of overlap between the two lists of DeepMito scores.

Similar to what was done with Fig. 2F, we considered the overlap in probability scores between two mitochondrial passenger proteins with various cut-offs (see Fig. 3C). Interestingly, the top N sorted probability scores reveals the presence of a possible passenger protein-specific synMTS ranking for each data set. Among the 100 synMTS with the highest scores across each data set, significant protein-specific import capabilities in the ranking could be observed with overlaps ranging from 20% to 58% for any two of the three data sets. However, the similarity in the ranking rapidly increases with the number of cumulated synMTS sequences, ranging between 75% and 85% in the top 500 synMTS of each of the three lists (dotted vertical line, Fig. 3C).

### Analysis of the mitochondrial import properties of the selected synMTS

After obtaining these three protein-specific rankings of our synMTS data set (Supplementary Fig. S1C), we explored the functionality of a subset of the ranked sequences *in vivo*. To select the sequences to be tested, we opted for a hypothesis-driven selection rather than a random selection of sequences. Indeed, as the core hypothesis of this study is to create a simple model generating new-to-nature and functional synMTS to be used in nuclear relocation experiments, we selected sequences based on their obtained DeepMito ranking to try isolating the best ones. We picked the synMTS with ranks of 1–5, 398–402, 798–802, and 1196–1200 (Supplementary Fig. S1C). Among the 60 sequences shortlisted across the three data sets, 50 were unique (these sequences are provided in Supplementary Table S4). Using SnapGene^®^ version 4.1.9 [41], we generated the corresponding yeast codon optimized DNA sequences (Supplementary Table S4) [42] which were then chemically synthesized by Twist Bioscience (South San Francisco, CA, USA).

First, we tested their ability to address to mitochondria a simple nonchemically challenging fluorescent reporter (Fig. 4). Using YeastFab assembly [34], we built the different expression vectors, transformed it into our BY4741 rho$^{+}$ strain and performed microscopy fluorescence observations including MitoTracker Red as a positive control. When the reporter was not associated with an MTS (“No MTS”), the fluorescence signal was present in the entire cell. When the reporter was associated with the reference MTS (“*OXA1* MTS” or “*SU9* MTS”), the signal was only detectable in the reticulated network of mitochondria, similar to the condition stained with MitoTracker Red. When associated with our synMTS, the results obtained revealed that a majority (33/50, or 66%) was functional and able to efficiently address the reporter to mitochondria (i.e. “synMTS_7_422” or “synMTS_4_361”). Of note, we observed a decreased efficiency in addressing the reporter to mitochondria with decreasing DeepMito scores (Supplementary Table S5).

**Figure 4.**
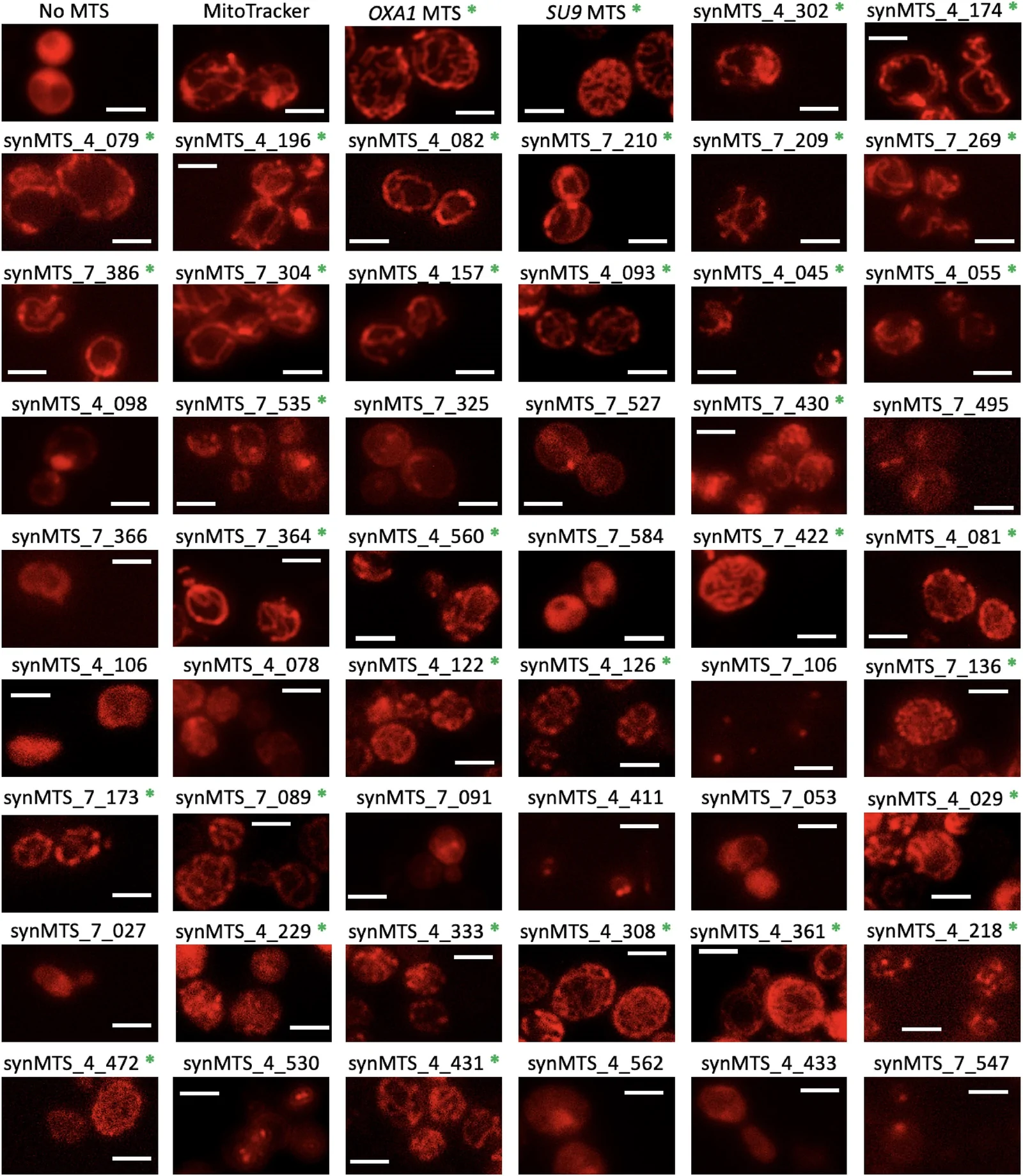
Fluorescence imaging of the BY4741 rho+ strain either stained with MitoTracker Red CMX ROS or expressing the different MTS-Cherry constructs. Scale bar = 5 *μ*m. The green asterisks denote a satisfactory addressing conferred by the corresponding synMTS.

The import properties of our synMTS were then further characterized using the IQ-Compete assay (an *in vivo* assay returning an import efficiency inversely correlated to the GFP signal measured) [20]. As compared to the original method, encoding the transcriptional units on low copy plasmids instead of a single genomic copy had a minor effect, with slightly decreased import efficiencies for the reference MTS (*MDL2, SU9, OXA1*, and *COX4* [20]). Regarding the values measured for our synMTS, the results demonstrated that all the sequences could import the uTev protease into mitochondria, with an average import efficiency of 35% (Fig. 5, see Supplementary Table S7 for the percentage values).

**Figure 5.**
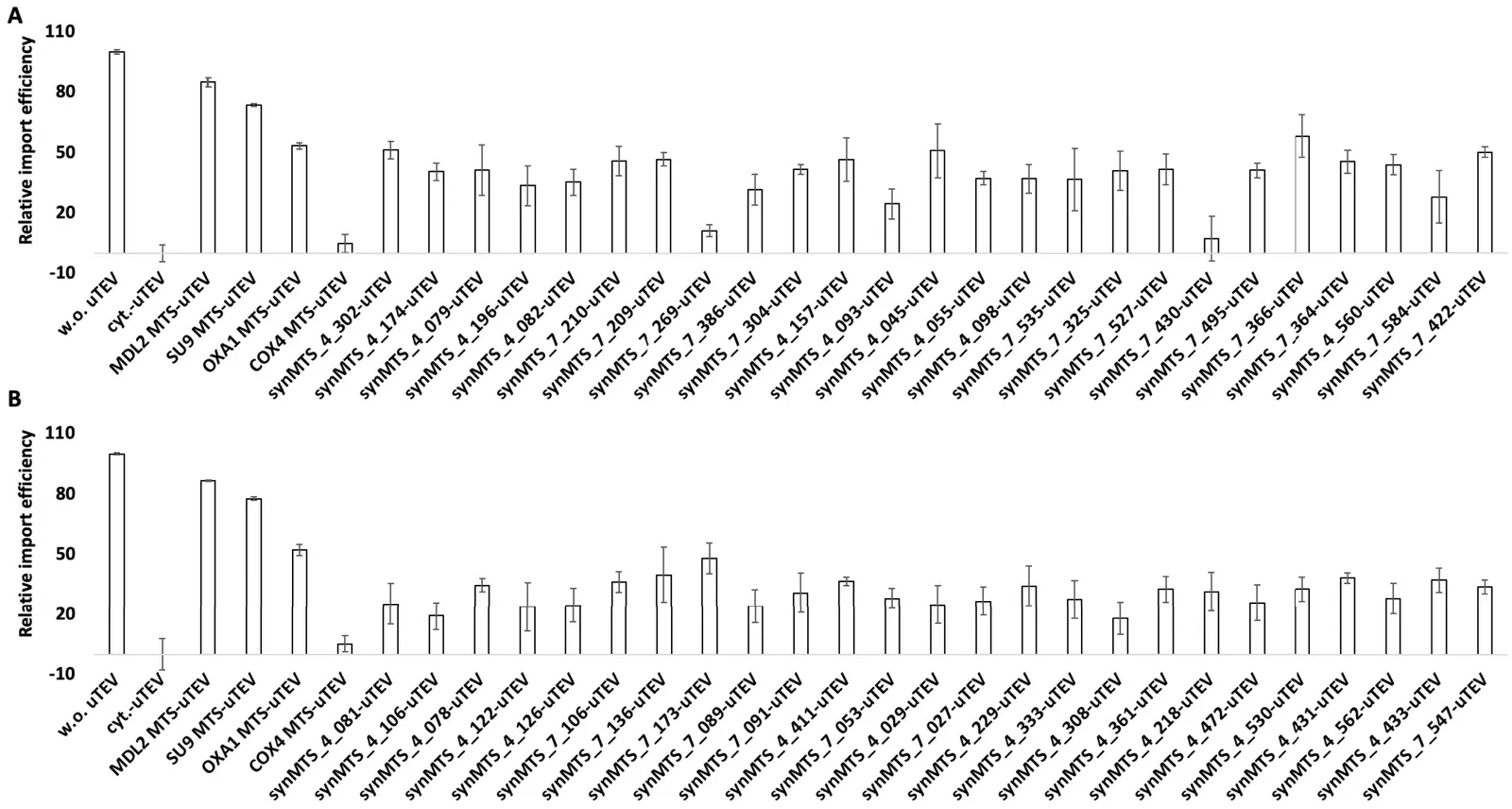
Results of the IQ-Compete assay performed using the reference MTS and the 50 unique synthetic presequences generated in this study. The relative import efficiency has been determined relative to the GFP signal measured in the condition where uTev is cytosolic (cyt.uTEV, no MTS added simulating 0% import) and the condition without uTev (w.o.uTEV, simulating 100% of import). For each condition, the data were measured in three independent biological repeats and are expressed as mean ± standard deviation.

Finally, the last test performed involved assessing the capability of our presequences to target Cox4p to mitochondria in a *COX4* deletion mutant strain. Using YeastFab [34], we built the different expression vectors and transformed it into the *COX4* deletion strain. Their performance was evaluated based on their ability to restore the respiratory growth of the *COX4* deletion mutant (see Fig. 6). When we expressed the *COX4* ORF with its native presequence, the respiratory growth of the *COX4* deletion mutant could be restored. However, this did not happen when the *COX4* MTS (condition referred to as “No MTS”) was removed. Using the *OXA1* and *SU9* MTS also enabled the restoration of respiratory growth of the deletion strain. When testing our 50 synMTS, we observed that >80% (42/50, Fig. 6) were also able to target Cox4p to mitochondria, confirming the correctness of the rules used to design our synthetic presequences.

**Figure 6.**
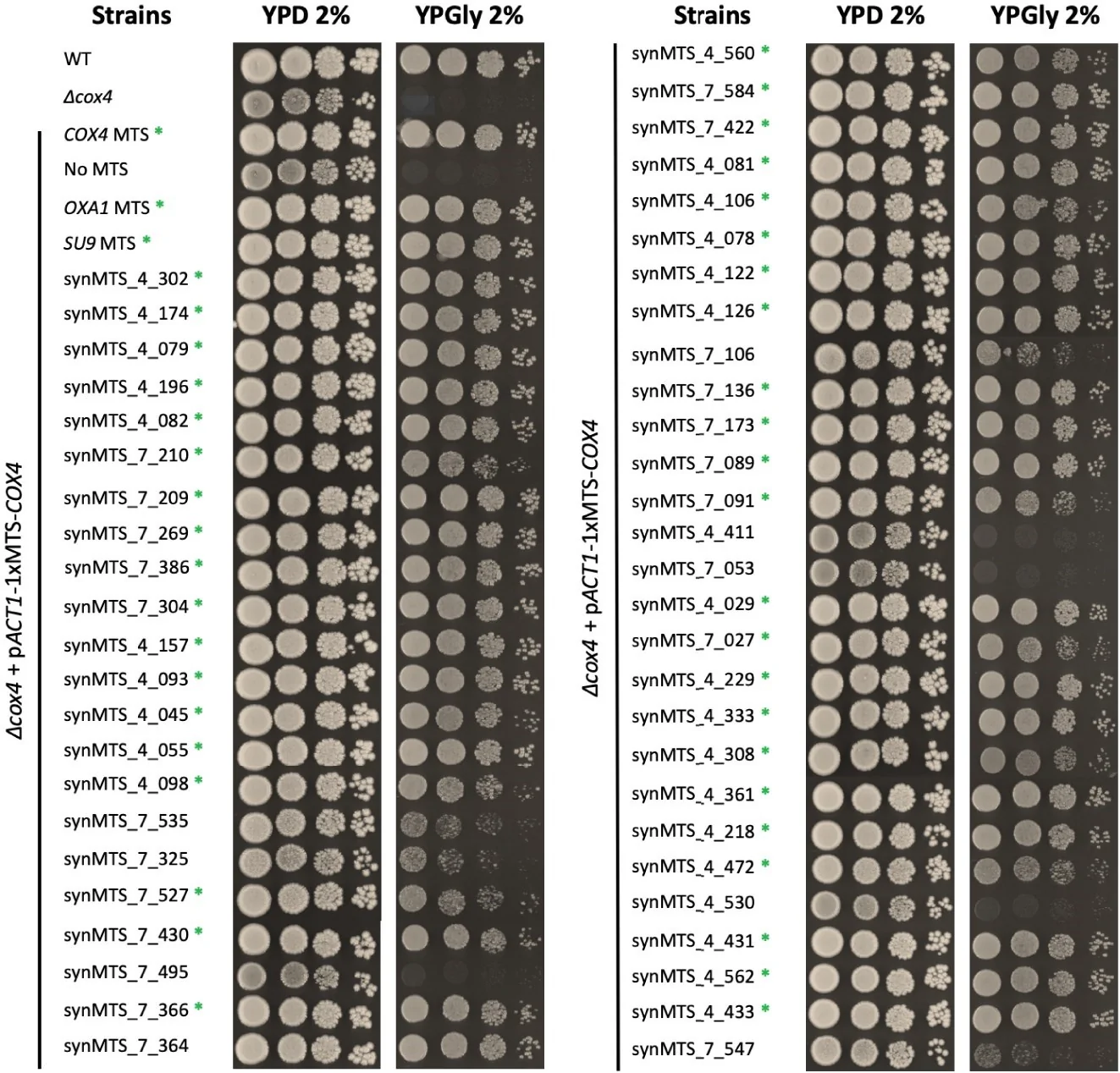
Spot-test presenting the growth benefit conferred by using reference or synthetic MTS to address Cox4p to mitochondria in the COX4 deletion strain. The pictures were taken after incubating the plates for 3 days at 30$^\circ$C. The green asterisks denote a satisfactory addressing conferred by the corresponding synMTS.a

### Functional validation of the selected synMTS with allotopic cargos

We then performed a first screening of our presequences testing the synMTS ability to promote the mitochondrial import of three artificially nucleus-relocated allotopic proteins (Cox2p-W56R, Atp9p, or Atp8p). The different expression vectors were assembled using the YeastFab assembly [34] with the adaptations previously described [18]. Based on our previous observations [18], the *COX2-W56R* gene was encoded on a low-copy vector with one MTS copy under the control of the *ICL1* promoter. These expression conditions offer the best respiratory growth dynamics to identify the improvements conferred by the synMTS [18]. The constructs were then delivered in our *COX2* deletion strain and tested for their ability to restore the deletion mutant’s respiratory growth (Fig. 7). Regarding *ATP8* and *ATP9* expression conditions, our preliminary tests (data not shown) suggest the use of a multicopy vector and the *ACT1* promoter. Concerning the MTS copy number, decent growth was observed when Atp8p was associated with a single MTS copy. In contrast, with Atp9p, our preliminary tests revealed that a single copy of *SU9* MTS could not promote Atp9p import (data not shown). Instead, we observed a minimal restoration of the respiratory growth when two copies were used. Thus, all the expression vectors for Atp9p in the present paper were built with two MTS copies. The spot-test assays performed in the *ATP9* and *ATP8* deletion strains are presented in Figs 8 and 9, respectively.

**Figure 7.**
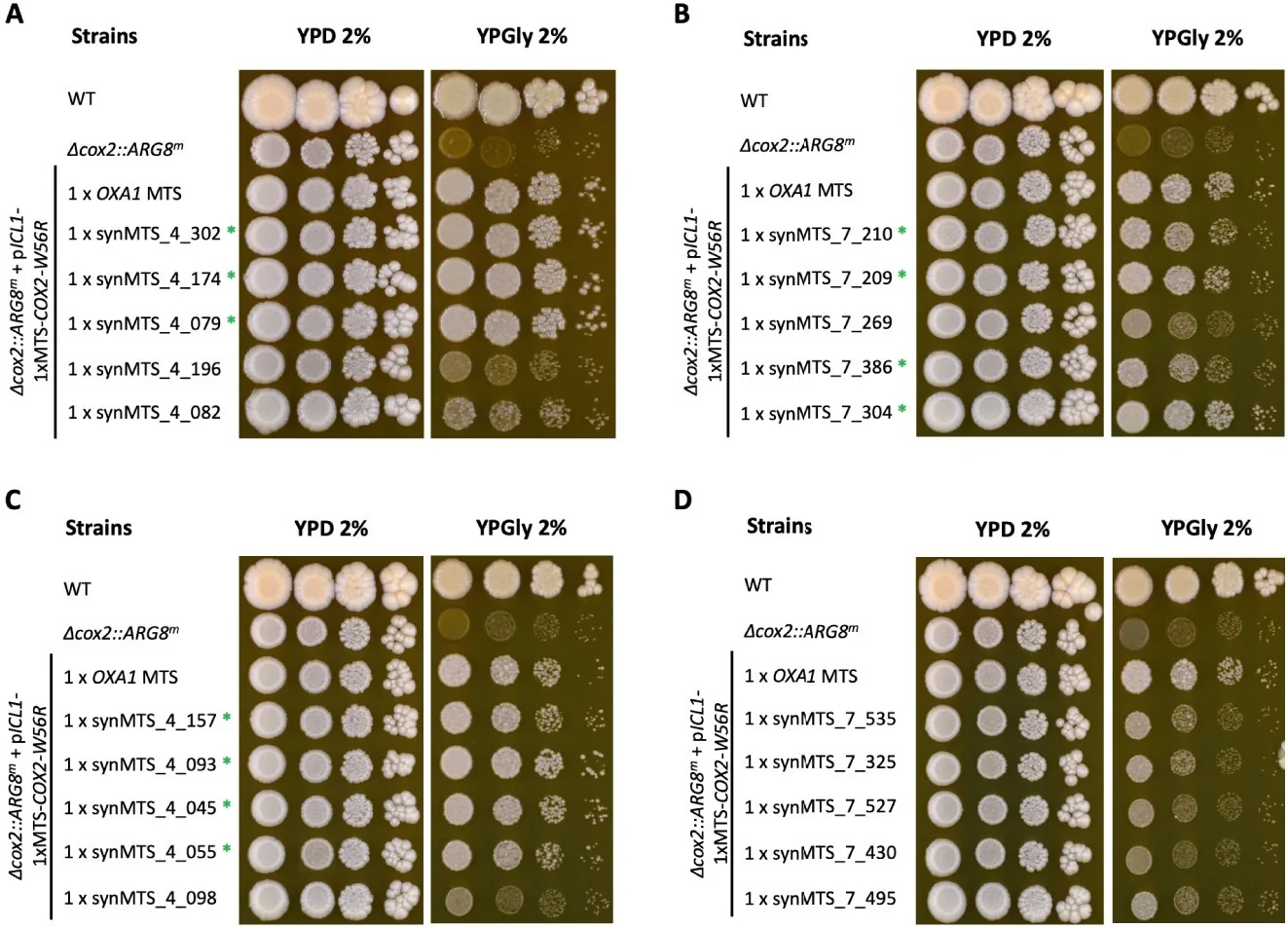
Spot-tests presenting the respiratory growth benefit conferred by the different sets of synthetic MTS in the *COX2-W56R* deletion strain. The sequences were ranked based on their DeepMito scores and sampled at different positions throughout the data set: (**A**) from 1 to 5, (**B**) from 398 to 402, (**C**) from 798 to 802 and (**D**) from 1196 to 1200. The pictures were taken after incubating the plates for 6 days at 30$^\circ$C. The green asterisks denote a satisfactory addressing conferred by the corresponding synMTS.

**Figure 8.**
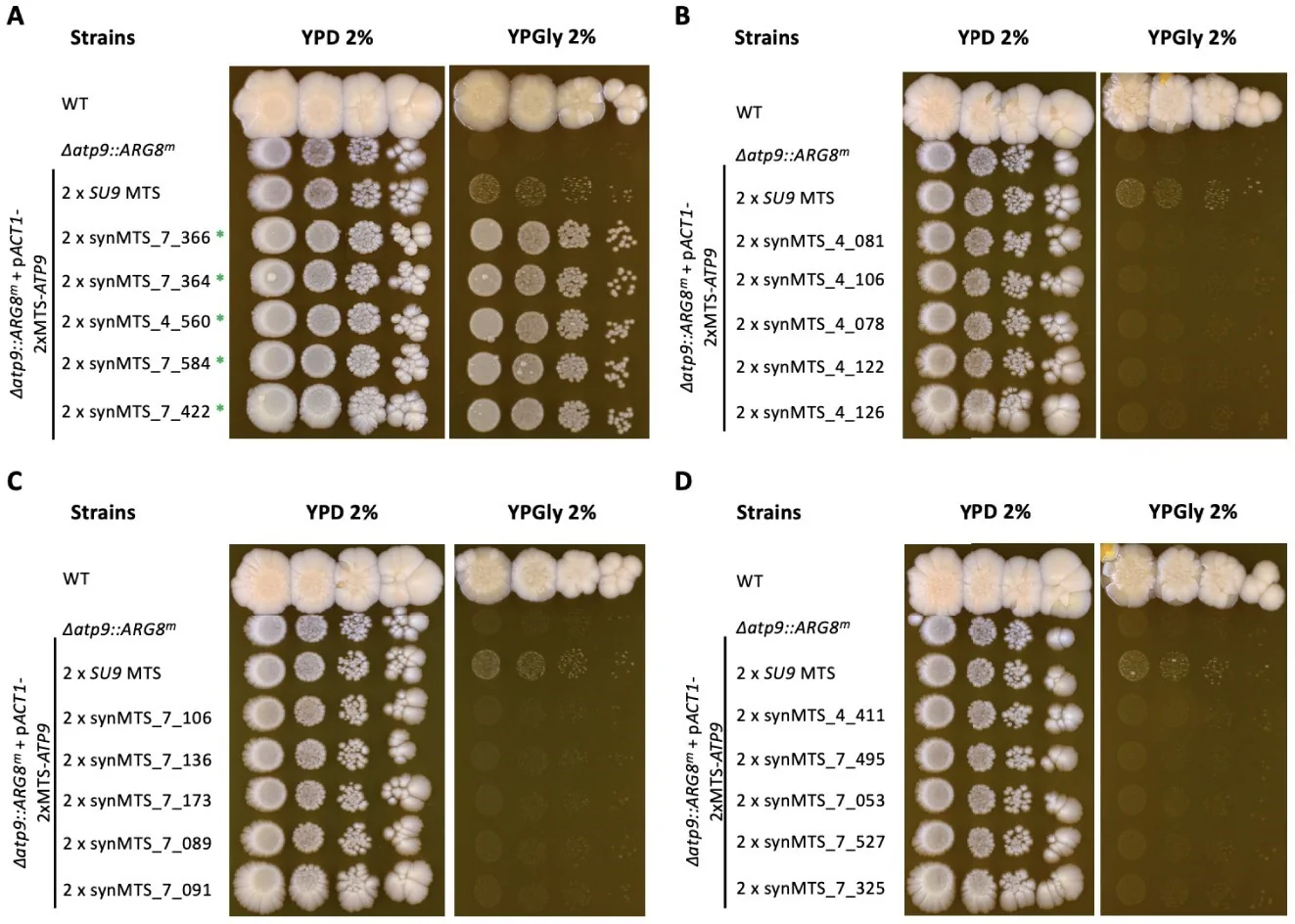
Spot-tests presenting the respiratory growth benefit conferred by the different sets of synthetic MTS in the *ATP9* deletion strain. The sequences were ranked based on their DeepMito scores and sampled at different position throughout the data set: (**A**) from 1 to 5, (**B**) from 398 to 402, (**C**) from 798 to 802 and (**D**) from 1196 to 1200. The pictures were taken after incubating the plates for 10 days at 30$^\circ$C. The green asterisks denote a satisfactory addressing conferred by the corresponding synMTS.

**Figure 9.**
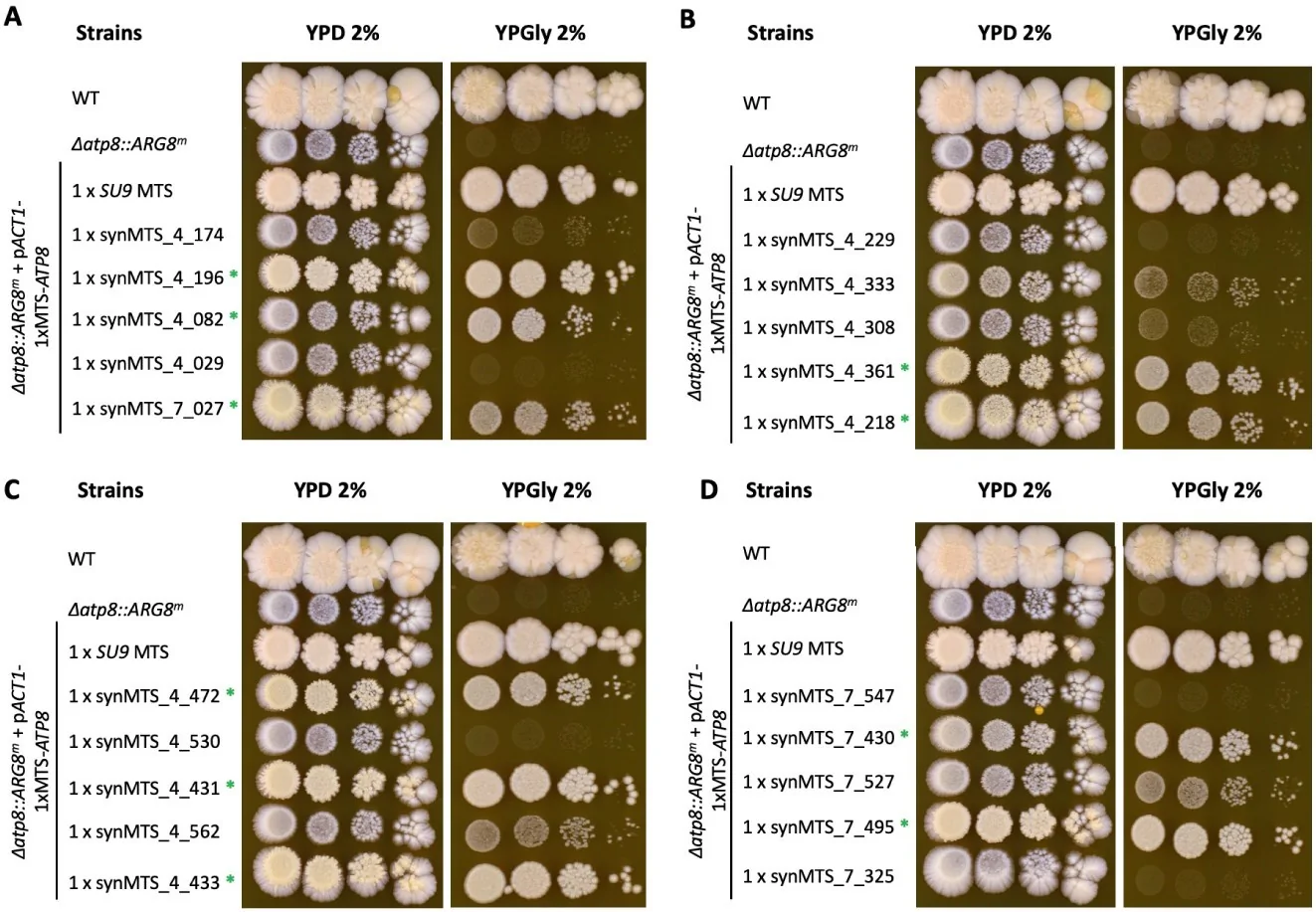
Spot-tests presenting the respiratory growth benefit conferred by the different sets of synthetic MTS in the *ATP8* deletion strain. The sequences were ranked based on their DeepMito scores and sampled at different position throughout the data set: (**A**) from 1 to 5, (**B**) from 398 to 402, (**C**) from 798 to 802, and (**D**) from 1196 to 1200. The pictures were taken after incubating the plates for 10 days at 30$^\circ$C. The green asterisks denote a satisfactory addressing conferred by the corresponding synMTS.

This first round of growth assays revealed that the ability of the tested synMTS-containing expression vectors to complement the respiratory growth of the different mitochondrial deletion strains drastically varied. Indeed, for both Cox2p-W56R and Atp8p, efficient synMTS were observed throughout the entire subsets of tested sequences (Figs 7 and 9). On the other hand, for Atp9p, only the five highest-ranking synMTS were able to partially restore the respiratory growth of the mutant (Fig. 8A). Moreover, for Cox2p-W56R and Atp8p, none of the tested synMTS surpassed the reference MTS sequence (either from *OXA1* or *SU9*). In contrast, fewer functional sequences were identified for Atp9p, but as compared to the condition using *SU9* MTS, they improved the *ATP9* deletion mutant’s respiratory growth, even though the impact on the OCR measured on whole cells was indiscernible (Supplementary Fig. S27B).

For both Cox2p-W56R and Atp8p, we repeated the growth experiment shortlisting the most efficient synMTS from this first round of spot-tests and taking pictures at shorter intervals. As only four potential candidates synMTS were identified for Atp8p, we also included the top 5 from Atp9p. Since both proteins belong to the F1/FO ATP synthase complex, we hypothesized that a potential synMTS cross-compatibility could exist. The results obtained for Cox2p-W56R and Atp8p are presented in Supplementary Figs S28 and S29, respectively.

For Cox2p-W56R, after 3 days of incubation we identified five synMTS that appeared as efficient as the *OXA1* MTS (Supplementary Fig. S28). We measured the levels of OCRs on whole cells and the results were similar between these conditions, confirming the efficiency of the shortlisted sequences in addressing Cox2p-W56R to mitochondria (Supplementary Fig. S27A).

For Atp8p (Supplementary Fig. S29), this second round of respiratory growth analysis revealed that: (i) none of the shortlisted sequences from the first set of spot-tests were as efficient as *SU9* MTS; and (ii) a partial synMTS inter-compatibility exists between Atp9p and Atp8p. More precisely, among the top five sequences from the Atp9p analyses, three were compatible with Atp8p (synMTS_7_364, synMTS_4_560, and synMTS_7_422, Supplementary Fig. S29). The measurement of OCRs in whole cells showed no significant differences compared to the condition using the *SU9* MTS (Supplementary Fig. S27C).

Based on the results obtained during the first screening on the different data sets of 20 synMTS, we decided to expand our analysis of the entire set of 50 synMTS to both Cox2p-W56R and Atp8p. Following the first screening, we decided to focus on these two proteins because: (i) only five out of the 20 synMTS tested were compatible with Atp9p (Fig. 8A); (ii) the restoration of the *ATP9* deletion mutant’s respiratory growth was only minimal; (iii) the expression vectors are complex to build as they require two copies of the synMTS; (iv) Atp9p is already encoded in the nucleus in humans, limiting translation. Due to its high hydrophobicity, Atp9p is an interesting stressful cargo to test synMTS import capabilities. Importantly, with TargetMITO, we particularly aim to help decipher the mechanisms of mitochondrial import of complex nucleus-relocated OXPHOS proteins. Since *COX2* and *ATP8* are also encoded by the mitochondrial genome in humans, the identification in yeast of functional synthetic presequences capable of supporting their allotopic expression provides a strong rationale for future translational studies. In particular, these synMTS could contribute to the development of improved nuclear gene-replacement strategies for primary mitochondrial diseases caused by pathogenic variants in *MT-CO2* or *MT-ATP8*. To perform these analyses, we built the remaining 30 expression vectors for both proteins, and tested the 50 synMTS for their ability to import either Cox2p-W56R (Fig. 10A) or Atp8p (Fig. 10B). Of the two cargos, Cox2p-W56R was the most permissive with 27/50 synMTS promoting a decent restoration of the mutant’s respiratory growth. On the other hand, the allotopic expression of Atp8p was more stringent, with only 16/50 synMTS able to promote a decent restoration of the respiratory growth. Interestingly, this expanded comparison clearly revealed discrepancies between our synMTS with presequences able to address efficiently either Cox2p-W56R, Atp8p, both cargos or none of them.

**Figure 10.**
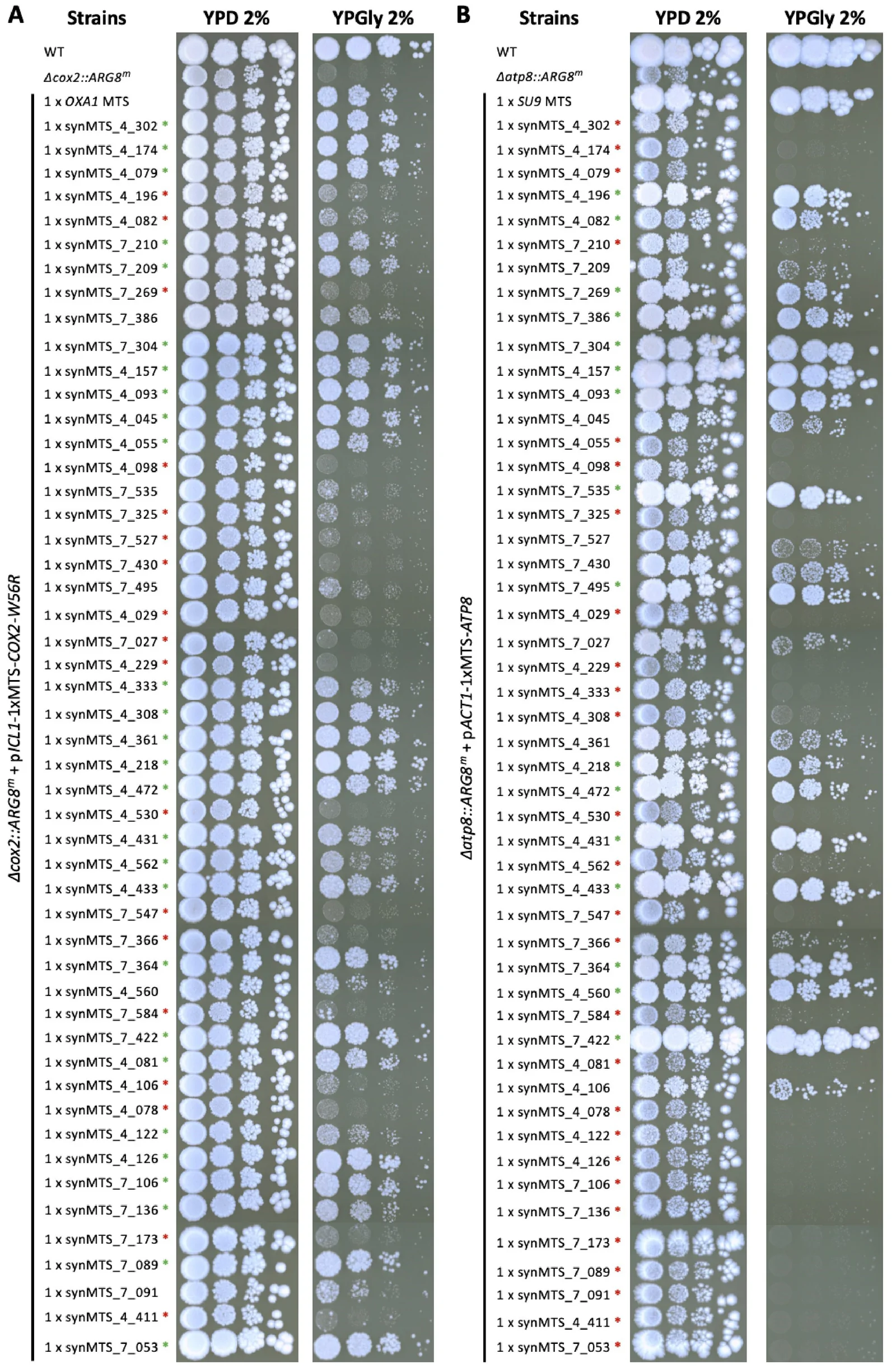
Spot-test presenting the respiratory growth benefit conferred by the allotopic expression systems associated with the 50 synthetic MTS in the *COX2* and *ATP8* deletion strains. The pictures were taken after incubating the plates for 6 or 10 days at 30$^\circ$C, respectively. The green asterisk denotes a satisfactory restoration of the respiratory growth while a red one indicates a nonsatisfactory respiratory growth.

We then focused on the most functional synMTS for each allotopic protein (Phenotype “++”, see Supplementary Table S11) and the less functional (Phenotype “−”, see Supplementary Table S11). We analysed the presequences composition [see Supplementary Table S12 (Cox2p-W56R), Supplementary Table S13 (Atp9p), and Supplementary Table S14 (Atp8p)] but could not find any significant difference for Cox2p-W56R and Atp8p data sets due to the high sequence diversity. However, the results for Atp9p revealed that the five functional synMTS were shorter, and consequently less charged, but due to the small sample size, this should be confirmed on a larger data set.

We also analysed the first six N-terminal residues as described by Nashed and collaborators [43] (Supplementary Fig. S30 and Supplementary Table S15) and tried discovering new motifs using Streme to explain presequences functionality (see Supplementary Figs S31–S36). Again, due to the small sample size and the high sequence diversity, these analyses did not return any significant differences between Cox2p-W56R and Atp8p sets of functional or nonfunctional synMTS. Again, a trend was observed with Atp9p where all compatible synMTS harboured a hydrophobic residue at position 2, whereas this was observed in less than half of Atp9p-incompatible candidates. In addition, most of the functional presequences contained valine at position 2, suggesting that *ATP9* may favour a broader hydrophobic N-terminal head than the canonical L/F/I/W signature described in natural yeast MTS, but due to the small sample size, this observation has to be repeated on a larger data set.

## Discussion

In the present study, we introduce TargetMITO, a new method to generate functional synthetic MTS using only four simple rules derived from both the known MTS characteristics and empirical observations. Our objective was to generate a robust method offering total control over the design parameters to generate passenger protein-specific synthetic MTS to shed new light on the mechanisms governing mitochondrial proteins import. We implemented nine different methods, generated more than 5000 synthetic MTS *in silico* (Supplementary Fig. S1A) and benchmarked them using two software (MitoFates and DeepMito). Two promising methods (4 and 7), characterized by the most stringent rules, were identified using MitoFates (Supplementary Fig. S1B), and the most beneficial trait identified imposed a regular pattern of arginine and lysine residues, which is essential for building both amphipathic motifs (Supplementary Figs S9–S11) and accumulated positive net charges (Supplementary Fig. S3), on to the sequences. This is in line with the results previously published on artificial MTS [4–8]. These parameters are known to be essential for the recognition of the mitochondrial protein precursor by the mitochondrial translocases of both the outer and inner membranes [3, 9, 10].

In light of the previously published studies, the first extensive research work on artificial MTS by Lemire and collaborators [7] reported that 25% of the randomly generated presequences could efficiently address Cox4p to mitochondria. With TargetMITO, we generated synMTS using rules capturing the known characteristics of natural MTS, and could achieve an efficiency higher than 80% (Fig. 6). These results confirm that presequences with a structure similar to natural MTS, instead of a random peptide sequence, are more able to promote the mitochondrial import of a simple cargo such as Cox4p. We also corroborated this observation during the side-by-side analyses we performed on the nine variational autoencoder-derived “artificial mitochondrial targeting sequences” (AMTS) validated in yeast in the recently published paper from Boob and collaborators (Supplementary Fig. S37B) [4].

In addition to generating functional synMTS, we aimed at producing presequences able to route artificially nucleus-relocated OXPHOS proteins (Cox2p-W56R, Atp9p, and Atp8p) in yeast. These mtDNA-encoded proteins are usually highly hydrophobic and extremely difficult to express from the nucleus as they are prone to cytosolic aggregation, incorrect addressing or impaired mitochondrial import [13, 16]. While engineering their protein sequence to artificially decrease hydrophobicity improves the mitochondrial import and maturation [13, 16, 18], a substantial fraction of these proteins still remains unprocessed in the cytosol. In addition, as the use of natural MTS is extremely limited due to a significant lack of MTS inter-compatibility [18, 19], there is a need for enhanced strategies to optimize mitochondrial allotopic proteins uptake through engineered MTS. Instead of screening randomly sampled synthetic sequences in our data sets, we tested if the DeepMito prediction scores could indicate a high probability of passenger-protein specific synergies between our synMTS and the different allotopic proteins (see Supplementary Figs S1C, and 3B and C). During the first round of screening, we observed that functional sequences were found throughout the entire panel of selected synMTS for both Cox2p-W56R and Atp8p (Figs 7 and 9). Instead, for Atp9p, only the TOP5 presequences were able to partially restore the respiratory growth of the corresponding deletion strain. These results confirm our application of DeepMito as a means to identify synMTS that are able to efficiently address allotopic proteins to mitochondria. In addition, for Atp9p, we were even able to identify synthetic presequences performing better than the gold-standard MTS from *SU9* gene (Fig. 8).

The last objective of our developed method involved highlighting clear instances of protein-specific synergies between our synMTS and the allotopic proteins in order to enlarge the set of functionally validated and protein-specific presequences. Enriching these data sets is essential to shedding light on the determinants of allotopic mitochondrial proteins import. To this, we extended the characterization of the 50 synMTS to Cox2p-W56R and Atp8p, as both homologues in humans are encoded in the mitochondrial genome, offering thus a foundation for future translational studies. We observed instances of passenger-specific functionalities (i.e. synMTS_4_174 for Cox2p-W56R or synMTS_7_535 for Atp8p, Fig. 10) and also identified synMTS with broader compatibility (i.e. synMTS_7_422, Fig. 10). Unfortunately, due to sequence diversity between the sets of functional/nonfunctional presequences, we could not identify new determinants explaining the observed import efficiencies. Despite that, this second round of growth assays clearly highlighted that TargetMITO is a strong model able to generate functional presequences competent for allotopic expression. This was particularly strengthened by the results we obtained during the side-by-side comparison with Boob’s AMTS. Indeed, when we tested these AMTS for their ability to import the three allotopic proteins, none were able to promote the mitochondrial import of any of the three nucleus-relocated cargos (Supplementary Fig. S37C–E). These data confirmed that our simple model TargetMITO is capable of generating efficient and new-to-nature synthetic MTS able to promote the mitochondrial import of nucleus-relocated hydrophobic OXPHOS proteins.

In line with these results, Rödl and collaborators proposed that MTS contain a protein-specific priority code to efficiently address its cargo to the correct mitochondrial sub-compartment [20]. We hypothesize that this priority code is defined by the filler amino acids present between the repeated hydrophilic residues which create a physical-chemical pattern recognized by the import machineries and promotes efficient import and addressing. Indeed, in the present study, we observed clear instances of protein-specific import properties, but due to sequence diversity, we could not identify new motifs explaining functionality. Thus, we propose to set both the length and hydrophilic pattern in future studies to identify which motifs in the filler amino acids explain the functionality.

Of note, in this study, we demonstrated that to search for new determinants governing the mitochondrial import of nucleus-relocated proteins, assays must focus on assessing the targeting of the proteins of interest to mitochondria (i.e. spot-assays or OCR quantification). While indirect assays can be informative (i.e. addressing a fluorescent reporter to mitochondria, measuring import properties or routing a nonchallenging nucleus-encoded OXPHOS subunit), it cannot be used to infer their efficacy to address allotopic proteins. Importantly, while we only tested 50 out of the 1200 generated presequences, we could still identify functional sequences and observe clear instances of passenger protein-specific synergies (i.e. working with Cox2p-W56R but not with Atp8p), confirming the strength of our model. TargetMITO is not only aiming at producing “stronger” presequences, but it defines a privileged region of sequence space for building functional synMTS by generating a functional baseline from which finer determinants—likely encoded by the amino-acid composition and local organization between the regularly spaced basic residues—can modulate passenger-specific compatibility. The results presented herein confirm the existence of a cargo-dependent functional compatibility embedded within MTS [20]. By further expanding our set of functionally validated synMTS, we will be able to identify the determinants that confer the ability to import nucleus-relocated OXPHOS subunits.

To conclude, the outcome of this study is of a great interest when considering gene therapy-based approaches to treat primary mitochondrial diseases. Because the human homologues of *COX2* and *ATP8* are also encoded by human mitochondrial DNA, the identification of functional synthetic presequences for these cargos in yeast provides a foundation for future translational studies to validate their performance in human cells. These synMTS may ultimately help improve allotopic gene-therapy strategies for primary mitochondrial diseases caused by pathogenic mutations in *MT-CO2* or *MT-ATP8*.

## Supplementary Material

gkag986_Supplemental_Files

## Data Availability

The data underlying this article are available in the article, its online supplementary material, and the GitHub repository at https://github.com/the-cai-lab/synMTS and Zenodo at https://zenodo.org/records/22878575 (DOI: 10.5281/zenodo.22878575).

## References

[1] Malina C, Larsson C, Nielsen J. Yeast mitochondria: an overview of mitochondrial biology and the potential of mitochondrial systems biology. FEMS Yeast Res. 2018;18:foy040. 10.1093/femsyr/foy040 29788060

[2] Wallace DC . Why do we still have a maternally inherited mitochondrial DNA? Insights from evolutionary medicine. Annu Rev Biochem. 2007;2007:781–821. 10.1146/annurev.biochem.76.081205.150955 17506638

[3] Dudek J, Rehling P, van der Laan M. Mitochondrial protein import: common principles and physiological networks. Biochim Biophys Mol Cell Res. 2013;1833:274–285. 10.1016/j.bbamcr.2012.05.028 22683763

[4] Boob AG, Tan S-I, Zaidi A et al. Design of diverse, functional mitochondrial targeting sequences across eukaryotic organisms using variational autoencoder. Nat Commun. 2025;16:4151. 10.1038/s41467-025-59499-3 PMC12050285 40320395

[5] Allison DS, Schatz G. Artificial mitochondrial presequences. Proc Natl Acad Sci USA. 1986;83:9011–9015. 10.1073/pnas.83.23.9011 PMC387064 3024162

[6] Douglas MG, McCammon MT, Vassarotti A. Targeting proteins into mitochondria. Microbiol Rev. 1986;50:166–178. 10.1128/mr.50.2.166-178.1986 PMC373062 2941675

[7] Lemire BD, Fankhauser C, Baker A et al. The mitochondrial targeting function of randomly generated peptide sequences correlates with predicted helical amphiphilicity. J Biol Chem. 1989;264:20206–20215. 10.1016/S0021-9258(19)47048-8 2555347

[8] Roise D, Theiler F, Horvath SJ et al. Amphiphilicity is essential for mitochondrial presequence function. EMBO J. 1988;7:649–653. 10.1002/j.1460-2075.1988.tb02859.x PMC454369 3396537

[9] Araiso Y, Imai K, Endo T. Role of the TOM complex in protein import into mitochondria: structural views. Annu Rev Biochem. 2022;21:679–703. 10.1146/annurev-biochem-032620-104527 35287471

[10] Jain N, Chacinska A, Rehling P. Understanding mitochondrial protein import: a revised model of the presequence translocase. Trends Biochem Sci. 2025;50:585–595. 10.1016/j.tibs.2025.03.001 40155273

[11] Artika IM . Allotopic expression of mitochondrial genes: basic strategy and progress. Genes Dis. 2020;7:578–584. 10.1016/j.gendis.2019.08.001 PMC7729113 33335957

[12] Claros MG, Perea J, Shu Y et al. Limitations to *in vivo* import of hydrophobic proteins into yeast mitochondria. The case of a cytoplasmically synthesized apocytochrome b. Eur J Biochem. 1995;228:762–771. 7737175

[13] Supekova L, Supek F, Greer JE et al. A single mutation in the first transmembrane domain of yeast COX2 enables its allotopic expression. Proc Natl Acad Sci USA. 2010;107:5047–5052. 10.1073/pnas.1000735107 PMC2841951 20194738

[14] Popot JL, de Vitry C. On the microassembly of integral membrane proteins. Annu Rev Biophys Biophys Chem. 1990;19:369–403. 10.1146/annurev.bb.19.060190.002101 2194481

[15] Daley DO, Clifton R, Whelan J. Intracellular gene transfer: reduced hydrophobicity facilitates gene transfer for subunit 2 of cytochrome c oxidase. Proc Natl Acad Sci USA. 2002;99:10510–10515. 10.1073/pnas.122354399 PMC124958 12142462

[16] Bietenhader M, Martos A, Tetaud E et al. Experimental relocation of the mitochondrial ATP9 gene to the nucleus reveals forces underlying mitochondrial genome evolution. PLoS Genet. 2012;8:e1002876. 10.1371/journal.pgen.1002876 PMC3420929 22916027

[17] Nieto-Panqueva F, Rubalcava-Gracia D, Hamel PP et al. The constraints of allotopic expression. Mitochondrion. 2023;73:30–50. 10.1016/j.mito.2023.09.004 37739243

[18] Gombeau K, Hoffmann SA, Cai Y. A new set of mutations in the second transmembrane helix of the Cox2p-W56R substantially improves its allotopic expressionin *Saccharomyces cerevisiae*. Genetics. 2025;229:iyaf037. 10.1093/genetics/iyaf037 PMC12005268 40178993

[19] Chin RM, Panavas T, Brown JM et al. Optimized mitochondrial targeting of proteins encoded by modified mRNAs rescues cells harboring mutations in mtATP6. Cell Rep. 2018;22:2818–2826. 10.1016/j.celrep.2018.02.059 29539412

[20] Rödl S, Hoffman Y, Jung F et al. A protein-specific priority code in presequences determines the efficiency of mitochondrial protein import. PLoS Biology. 2025;23:e3003298. 10.1371/journal.pbio.3003298 PMC12306757 40690527

[21] Gorman GS, Schaefer AM, Ng Y et al. Prevalence of nuclear and mitochondrial DNA mutations related to adult mitochondrial disease. Ann Neurol. 2015;77:753–759. 10.1002/ana.24362 PMC4737121 25652200

[22] Soldatov VO, Kubekina MV, Skorkina MY et al. Current advances in gene therapy of mitochondrial diseases. J Transl Med. 2022;20:562. 10.1186/s12967-022-03685-0 PMC9724384 36471396

[23] Newman NJ, Man PY-W, Carelli V et al. Intravitreal gene therapy vs. natural history in patients with Leber hereditary optic neuropathy carrying the m.11778G> a ND4 mutation: systematic review and indirect comparison. Frontiers Neurol. 2021;12:662838 10.3389/fneur.2021.662838 PMC8181419 34108929

[24] Wan X, Pei H, Zhao M-J et al. Efficacy and safety of rAAV2-ND4 treatment for Leber’s hereditary optic neuropathy. Sci Rep. 2016;19:21587. 10.1038/srep21587 PMC4759604 26892229

[25] Fukasawa Y, Tsuji J, Fu S-C et al. MitoFates: improved prediction of mitochondrial targeting sequences and their cleavage sites. Mol Cell Proteomics. 2015;14:1113–1126. 10.1074/mcp.M114.043083 PMC4390256 25670805

[26] Savojardo C, Bruciaferri N, Tartari G et al. DeepMito: accurate prediction of protein sub-mitochondrial localization using convolutional neural networks. Bioinformatics. 2020;36:56–64. 10.1093/bioinformatics/btz512 PMC6956790 31218353

[27] Gavel Y, von Heijne G. Cleavage-site motifs in mitochondrial targeting peptides. Protein Eng. 1990;4:33–37. 10.1093/protein/4.1.33 2290832

[28] Hopp TP, Woods KR. Prediction of protein antigenic determinants from amino acid sequences. Proc Natl Acad Sci USA. 1981;78:3824–3828. 10.1073/pnas.78.6.3824 PMC319665 6167991

[29] Altschul SF, Madden TL, Schäffer AA et al. Gapped BLAST and PSI-BLAST: a new generation of protein database search programs. Nucleic Acids Res. 1997;25:3389–3402. 10.1093/nar/25.17.3389 PMC146917 9254694

[30] Pierleoni A, Martelli PL, Fariselli P et al. BaCelLo: a balanced subcellular localization predictor. Bioinformatics. 2006;22:e408–e416. 10.1093/bioinformatics/btl222 16873501

[31] Indio V, Martelli PL, Savojardo C et al. The prediction of organelle-targeting peptides in eukaryotic proteins with grammatical-restrained hidden conditional random fields. Bioinformatics. 2013;29:981–988. 10.1093/bioinformatics/btt089 23428638

[32] Savojardo C, Martelli PL, Fariselli P et al. TPpred3 detects and discriminates mitochondrial and chloroplastic targeting peptides in eukaryotic proteins. Bioinformatics. 2015;31:3269–3275. 10.1093/bioinformatics/btv367 26079349

[33] Bonnefoy N, Bsat N, Fox TD. Mitochondrial translation of *Saccharomyces cerevisiae* COX2 mRNA is controlled by the nucleotide sequence specifying the pre-Cox2p leader peptide. Mol Cell Biol. 2001;21:2359–2372. 10.1128/MCB.21.7.2359-2372.2001 PMC86869 11259585

[34] Guo Y, Dong J, Zhou T et al. YeastFab: the design and construction of standard biological parts for metabolic engineering in *Saccharomyces cerevisiae*. Nucleic Acids Res. 2015;43:e88. 10.1093/nar/gkv464 PMC4513847 25956650

[35] Rice P, Longden I, Bleasby A. EMBOSS: The European Molecular Biology Open Software Suite. Trends Genet. 2000;16:276–277. 10.1016/S0168-9525(00)02024-2 10827456

[36] Eisenberg D, Schwarz E, Komaromy M et al. Analysis of membrane and surface protein sequences with the hydrophobic moment plot. J Mol Biol. 1984;179:125–142. 10.1016/0022-2836(84)90309-7 6502707

[37] Eisenberg D, Weiss RM, Terwilliger TC. The helical hydrophobic moment: a measure of the amphiphilicity of a helix. Nature. 1982;299:371–374. 10.1038/299371a0 7110359

[38] Bailey TL . STREME: accurate and versatile sequence motif discovery. Bioinformatics. 2021;37:2834–2840. 10.1093/bioinformatics/btab203 PMC8479671 33760053

[39] Köster J, Rahmann S. Snakemake–a scalable bioinformatics workflow engine. Bioinformatics. 2012;28:2520–2522. 10.1093/bioinformatics/bts480 22908215

[40] Nagley P, Farrell LB, Gearing DP et al. Assembly of functional proton-translocating ATPase complex in yeast mitochondria with cytoplasmically synthesized subunit 8, a polypeptide normally encoded within the organelle. Proc Natl Acad Sci USA. 1988;85:2091–2095. 10.1073/pnas.85.7.2091 PMC279934 2895470

[41] GraphPad Software, LLC . Snapgene software, version 4.1. https://www.snapgene.com/ (1 September 2026, date last accessed).

[42] Nakamura Y, Gojobori T, Ikemura T. Codon usage tabulated from international DNA sequence databases: status for the year 2000. Nucleic Acids Res. 2000;28:292. 10.1093/nar/28.1.292 PMC102460 10592250

[43] Nashed S, Barbry HE, Benchouaia M et al. Functional mapping of N-terminal residues in the yeast proteome uncovers novel determinants for mitochondrial protein import. PLoS Genet. 2023;19:e1010848. 10.1371/journal.pgen.1010848 PMC10482271 37585488

