# Supplementary material for: TargetMITO: a rule-based model for generating functional synthetic mitochondrial targeting sequences in yeast
Source: Nucleic Acids Res. 2026 Oct 21;54(19):gkag986. doi: 10.1093/nar/gkag986 (PMC13684279; doi:10.1093/nar/gkag986)
Supplement: Supplementary file 1 [file gkag986_supplemental_files.zip › supplementary.pdf]

TargetMITO: A rule-based model for generating functional synthetic mitochondrial targeting sequences in yeast

Kewin Gombeau,[1,*] Raymond Wan,[1,*] Joshua S. James,[1] Déborah Tribouillard-Tanvier[2] and Yizhi Cai[1,*]

[1]Manchester Institute of Biotechnology, University of Manchester, 131 Princess Street, M1 7DN, Manchester, United Kingdom
[2]CNRS, IBGC, UMR 5095, University of Bordeaux, F-33000 Bordeaux, France

[*]Corresponding authors kewin.gombeau@manchester.ac.uk and raymond.wan@manchester.ac.uk and yizhi.cai@manchester.ac.uk

Contents

1 Methods 3
  1.1 synMTS Generation 3
2 Results 5
  2.1 Design and validation of novel synthetic MTS 5
  2.2 Functional validation of the selected synMTS 18

List of Figures

S1 Summary of the data sets 3
S2 Distribution of synMTS lengths for the 9 different methods. 5
S3 Distribution of total net charges of the synMTS for the 9 different methods. 6
S4 Scatter plot of the MitoFates prediction scores using Cox2p-W56R showing the effects from replacing arginine with lysine. 6
S5 Scatter plot of the MitoFates prediction scores using Atp9p showing the effects from replacing arginine with lysine. 7
S6 Scatter plot of the MitoFates prediction scores using Atp8p showing the effects from replacing arginine with lysine. 7
S7 Scatter plot of the MitoFates prediction scores using Hac1p showing the effects from replacing arginine with lysine. 8
S8 Scatter plot of the MitoFates prediction scores using Mmf1p showing the effects from replacing arginine with lysine. 8
S9 Distribution of the maximum hydrophobic moments of each synMTS. 9
S10 Distribution of the average of the top 10% hydrophobic moments of each synMTS. 9
S11 Distribution of the average hydrophobic moments of each synMTS. 10
S12 Scatter plot of maximum hydrophobic moment values against MitoFates prediction scores (Cox2p-W56R). 10
S13 Scatter plot of maximum hydrophobic moment values against MitoFates prediction scores (Atp9p). 11
S14 Scatter plot of maximum hydrophobic moment values against MitoFates prediction scores (Atp8p). 11
S15 Scatter plot of maximum hydrophobic moment values against MitoFates prediction scores (Hac1p). 12
S16 Scatter plot of maximum hydrophobic moment values against MitoFates prediction scores (Mmf1p). 12
S17 Scatter plot of average of the top 10% hydrophobic moment values against MitoFates prediction scores (Cox2p-W56R). 13
S18 Scatter plot of average of the top 10% hydrophobic moment values against MitoFates prediction scores (Atp9p). 13
S19 Scatter plot of average of the top 10% hydrophobic moment values against MitoFates prediction scores (Atp8p). 14
S20 Scatter plot of average of the top 10% hydrophobic moment values against MitoFates prediction scores (Hac1p). 14
S21 Scatter plot of average of the top 10% hydrophobic moment values against MitoFates prediction scores (Mmf1p). 15
S22 Scatter plot of average hydrophobic moment values against MitoFates prediction scores (Cox2p-W56R). 15
S23 Scatter plot of average hydrophobic moment values against MitoFates prediction scores (Atp9p). 16
S24 Scatter plot of average hydrophobic moment values against MitoFates prediction scores (Atp8p). 16
S25 Scatter plot of average hydrophobic moment values against MitoFates prediction scores (Hac1p). 17
S26 Scatter plot of average hydrophobic moment values against MitoFates prediction scores (Mmf1p). 17
S27 Oxygen consumption rates on whole cells following the first round of screening. 18
S28 Time-course spot-test of the shortlisted candidates synMTS for *COX2-W56R* allotopic expression. 18
S29 Time-course spot-test of the shortlisted candidates synMTS for *ATP8* allotopic expression. 19
S30 Analysis of the N-terminal composition in the sets of functional/non-functional presequences. 24
S31 Ungapped motif discovery using the usual amino acids (Cox2p-W56R). 25
S32 Ungapped motif discovery using amino acid categories of Table S10 (Cox2p-W56R). 25
S33 Ungapped motif discovery using the usual amino acids (Atp9p). 26
S34 Ungapped motif discovery using amino acid categories of Table S10 (Atp9p). 26
S35 Ungapped motif discovery using the usual amino acids (Atp8p). 27
S36 Ungapped motif discovery using amino acid categories of Table S10 (Atp8p). 27
S37 Side-by-side comparison with Boob and collaborators VAE-based AMTS. 28

List of Tables

S8 Regular expressions for synMTS generation. 3
S9 Amino acid hydrophilicity scale. 4
S10 Remapping of 20 canonical amino acids for Streme. 4
S11 Summary of the visual assessments of each synMTS. 20
S12 Properties of the synMTS sequences (Cox2p-W56R). 21
S13 Properties of the synMTS sequences (Atp9p). 21
S14 Properties of the synMTS sequences (Atp8p). 21
S15 synMTS sequences used for motifs discovery and generation of N6 logos. 23

1. Methods

1.1. synMTS Generation

Method	Regular expression
1	$\mathsf{M}(\mathcal{A})^{+}$
2	$\mathsf{M}\mathcal{A}^{\ +}(\mathsf{R}\mathcal{A}\mathsf{Y}^{\{0,1\}})$
3	$\mathsf{M}\mathcal{A}^{\ *}(\mathcal{H}_1^{\ +}\mathcal{A}^{\ *})^{+}(\mathsf{R}\mathcal{A}\mathsf{Y}^{\{0,1\}})$
4	$\mathsf{M}\mathcal{O}^{\ \{2,4\}}(\mathcal{H}_1\ \mathcal{O}^{\ \{2,4\}})^{+}(\mathsf{R}\mathcal{A}\mathsf{Y}^{\{0,1\}})$
5	$\mathsf{M}\mathcal{O}^{\ \{2,4\}}(\mathcal{H}_2\ \mathcal{O}^{\ \{2,4\}})^{+}(\mathsf{R}\mathcal{A}\mathsf{Y}^{\{0,1\}})$
6	$\mathsf{M}\mathcal{O}^{\ \{2,4\}}(\mathcal{H}\ \mathcal{O}^{\ \{2,4\}})^{+}(\mathsf{R}\mathcal{A}\mathsf{Y}^{\{0,1\}})$
7	$\mathsf{M}\mathcal{O}^{\ \{2,4\}}(\mathcal{H}_1^{\ \{1,2\}}\mathcal{O}^{\ \{2,4\}})^{+}(\mathsf{R}\mathcal{A}\mathsf{Y}^{\{0,1\}})$
8	$\mathsf{M}\mathcal{O}^{\ \{2,4\}}(\mathcal{H}_2\ \mathcal{H}_1^{\ \{0,1\}}\mathcal{O}^{\ \{2,4\}})^{+}(\mathsf{R}\mathcal{A}\mathsf{Y}^{\{0,1\}})$
9	$\mathsf{M}\mathcal{O}^{\ \{2,4\}}(\mathcal{H}\ \mathcal{H}_1^{\ \{0,1\}}\mathcal{O}^{\ \{2,4\}})^{+}(\mathsf{R}\mathcal{A}\mathsf{Y}^{\{0,1\}})$

Table S8 Regular expressions for the 9 methods used to generate the synMTS presequences.

The description of our 9 methods in the main text is supplemented with regular expressions in Table S8. The characters M, R, and Y represent amino acids (specifically, methionine, arginine, and tyrosine, respectively) while characters in a cursive font (i.e., $\mathcal{H}$, $\mathcal{H}_1$, $\mathcal{H}_2$, $\mathcal{O}$, and $\mathcal{A}$) represent *sets* of amino acids. Superscripts indicate how many times an amino acid or an element in a set occurs. For example, a "*" indicates that the amino acid or set element occurs 0 or more times, while a "+" indicates that there is one or more occurrences. A pair of numbers in braces, such as $2, 4$, indicates that the amino acid or set element occurs 2 to 4 times. Some of these notations are part of the extended regular expressions, which are used by various programming languages (4).

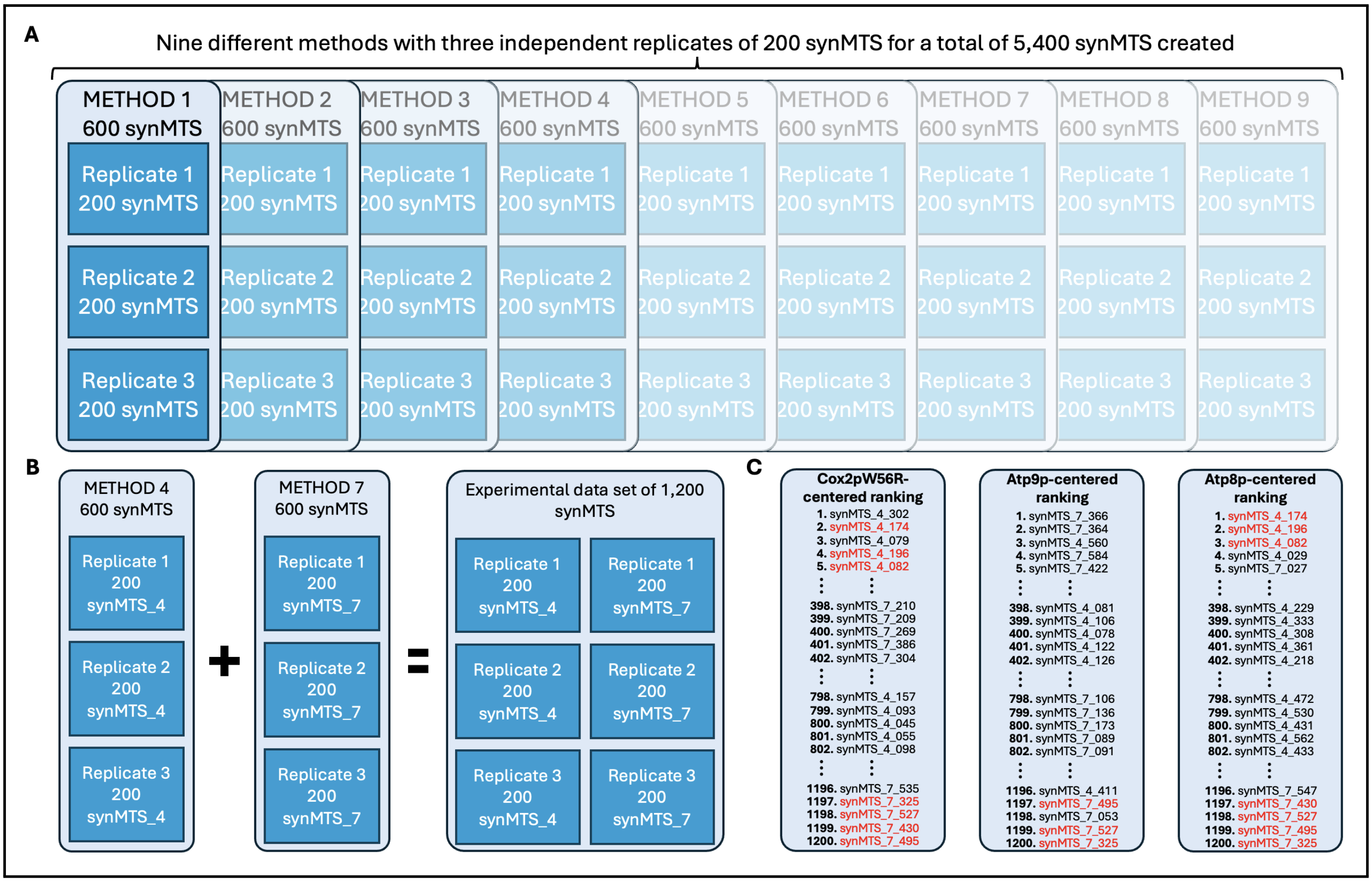

Figure S1 Summary of the data sets created and the partitioning employed by this study. (A) Three replicates of 200 synMTS each were created for each of the 9 methods. (B) The highest scoring methods according to MitoFates were Methods 4 and 7. The synMTS of these two methods were combined into a single data set. (C) These synMTS were fused with one of three passenger proteins (Cox2p, Atp9p, and Atp8p) and ranked according to DeepMito. The synMTS highlighted in red are present in at least two data sets.

Amino acid	Abbreviation	Hydrophilicity
alanine	A	-0.5
cysteine	C	-1
aspartate	D	3
glutamate	E	3
phenylalanine	F	-2.5
glycine	G	0
histidine	H	-0.5
isoleucine	I	-1.8
lysine	K	3
leucine	L	-1.8
methionine	M	-1.3
asparagine	N	0.2
proline	P	0
glutamine	Q	0.2
arginine	R	3
serine	S	0.3
threonine	T	-0.4
valine	V	-1.5
tryptophan	W	-3.4
tyrosine	Y	-2.3

Table S9 Amino acid hydrophilicity scale for the 20 canonical amino acids (5).

Category	Amino acids	Replacement
Hydrophobic aliphatic	A I L M P V	A
Hydrophobic aromatic	F W	F
Glycine	G	G
Cysteine	C	C
Hydrophilic polar uncharged	N Q S T Y	N
Hydrophilic polar basic (positive charge)	H K R	H
Hydrophilic polar acidic (negative charge)	D E	D

Table S10 Summary of how the 20 canonical amino acids were remapped to characters based on amino acid category (7), prior to processing by Streme. For example, the six amino acids which are hydrophobic aliphatic (first row) were all substituted with an "A".

2. Results

2.1. Design and validation of novel synthetic MTS

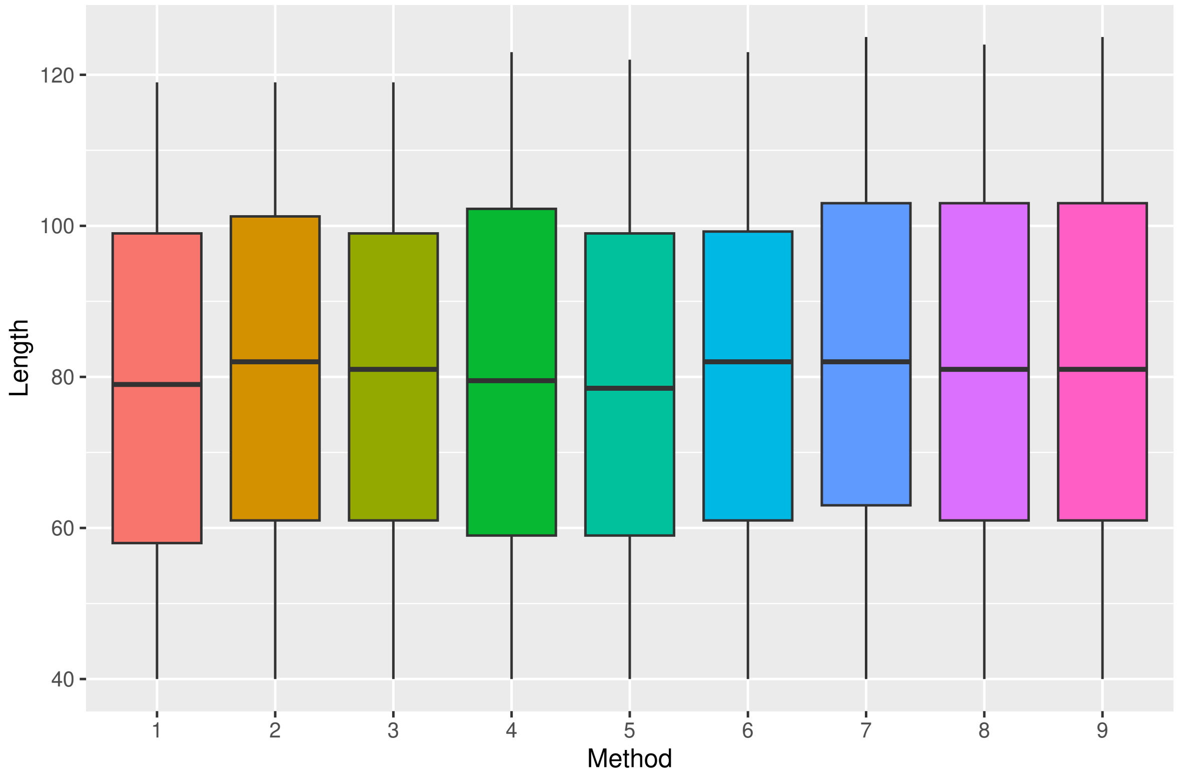

Figure S2 Distribution of synMTS lengths for the 9 different methods.

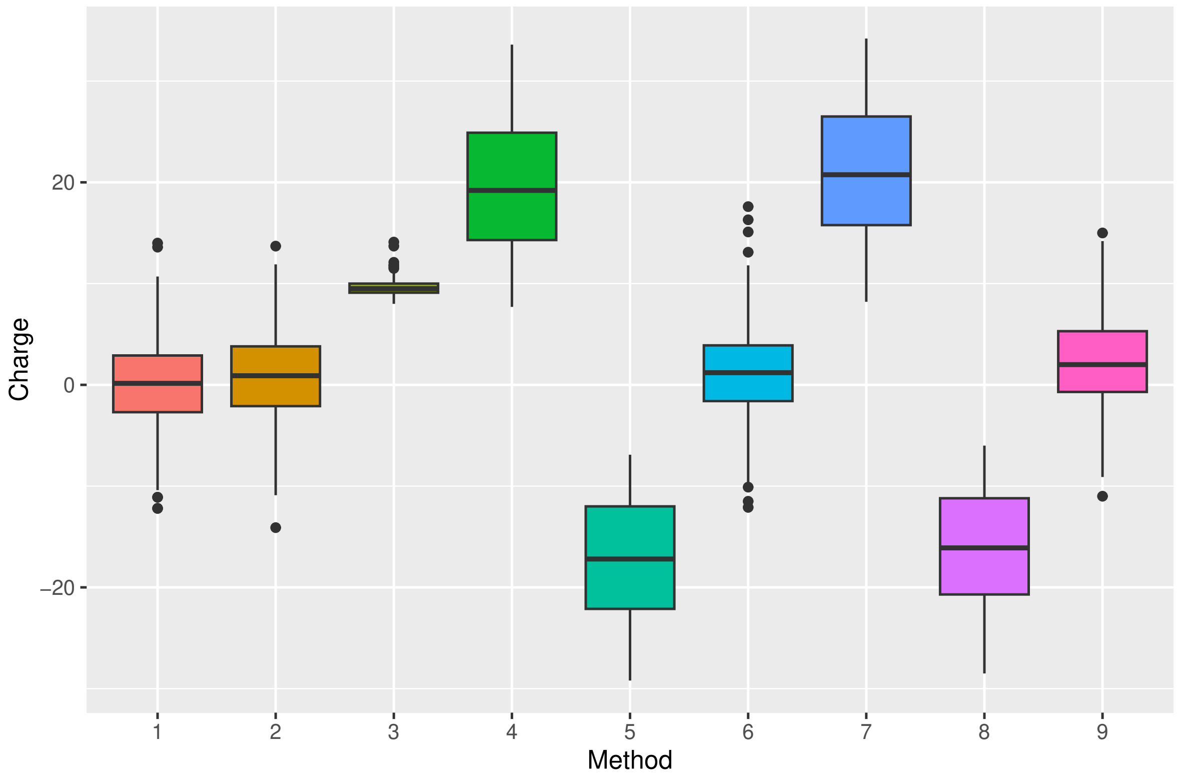

Figure S3 Distribution of total net charges of the synMTS for the 9 different methods.

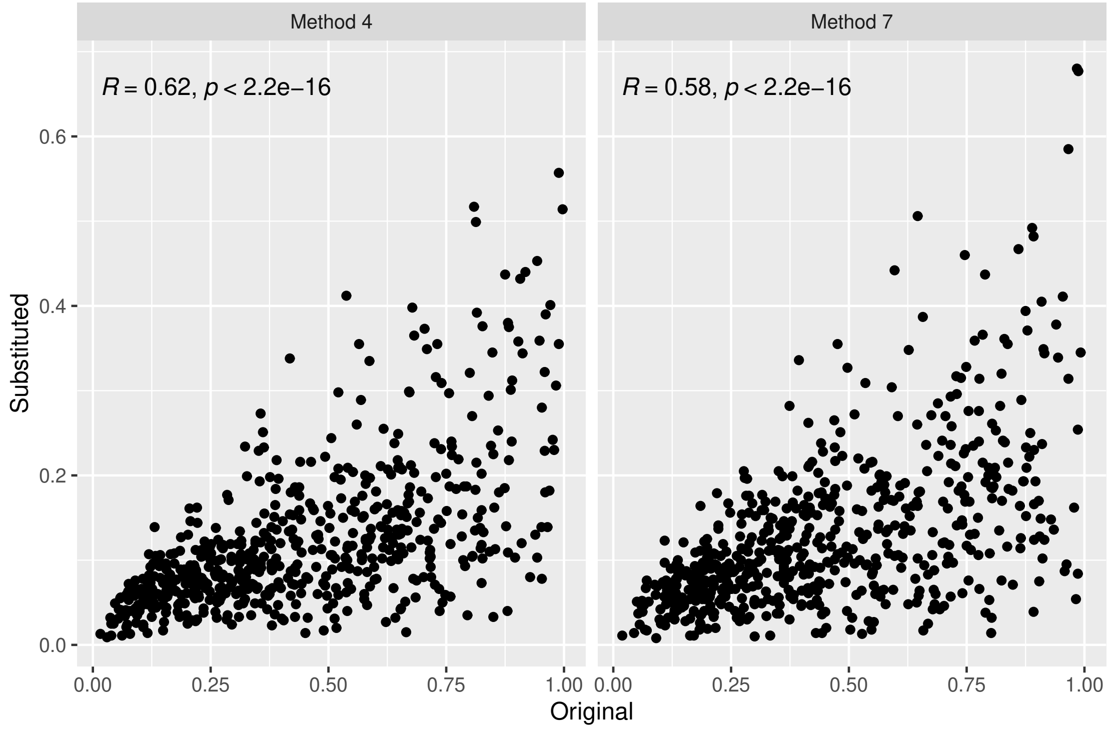

Figure S4 Scatter plot of the MitoFates prediction scores obtained for each individual synMTS from Methods 4 or 7 after appending Cox2p-W56R, using either the original design rules or substituting all of the arginines, except the final one involved in defining the mitochondrial peptidase cleavage site, for lysine. Each method contains 600 synMTS sequences. Spearman correlation coefficient was calculated using R version 4.4.2.

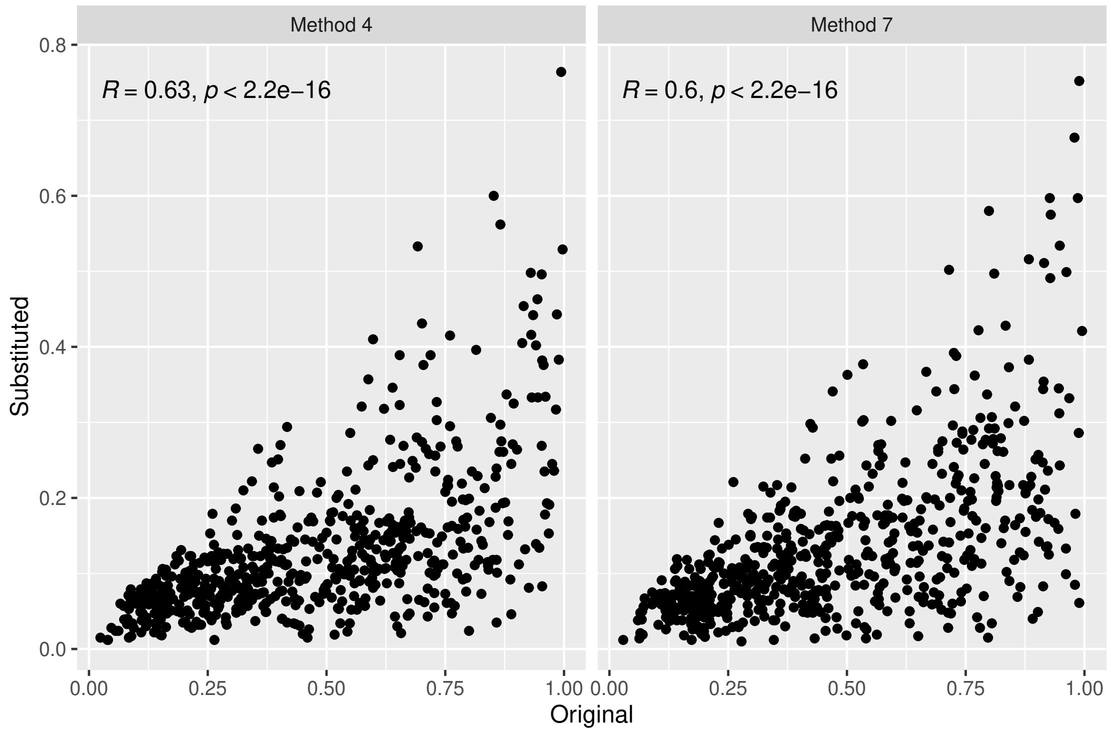

Figure S5 Scatter plot of the MitoFates prediction scores obtained for each individual synMTS from Methods 4 or 7 after appending Atp9p, using either the original design rules or substituting all of the arginines, except the final one involved in defining the mitochondrial peptidase cleavage site, for lysine. Each method contains 600 synMTS sequences. Spearman correlation coefficient was calculated using R version 4.4.2.

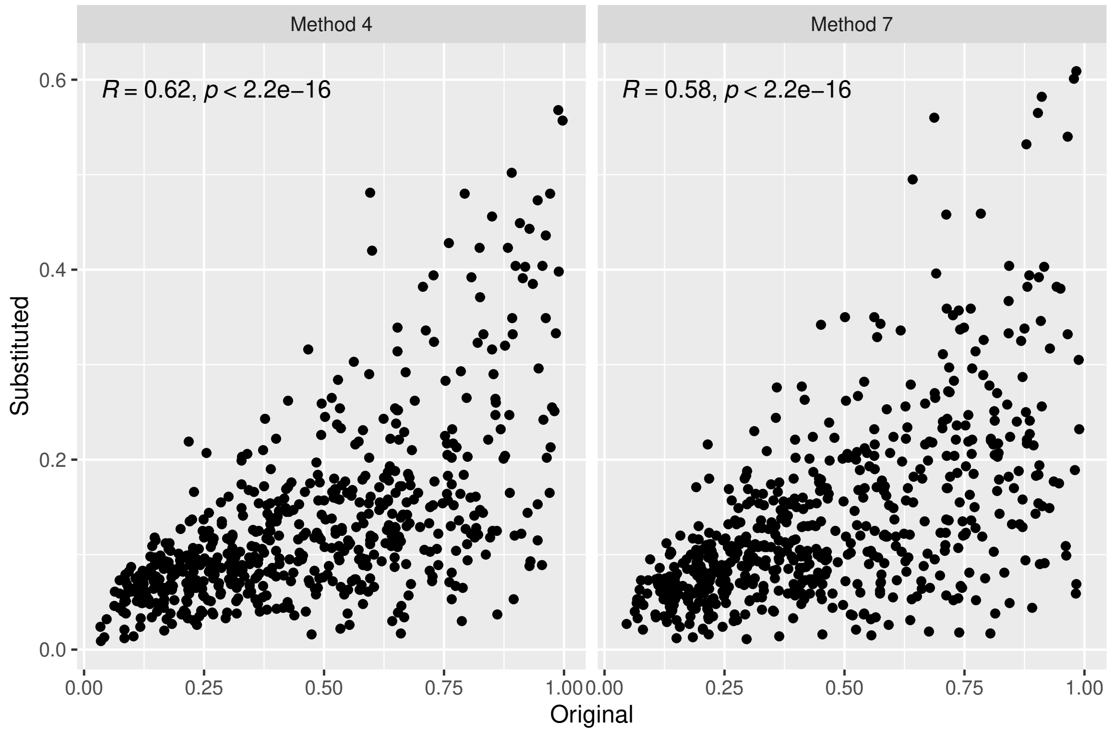

Figure S6 Scatter plot of the MitoFates prediction scores obtained for each individual synMTS from Methods 4 or 7 after appending Atp8p, using either the original design rules or substituting all of the arginines, except the final one involved in defining the mitochondrial peptidase cleavage site, for lysine. Each method contains 600 synMTS sequences. Spearman correlation coefficient was calculated using R version 4.4.2.

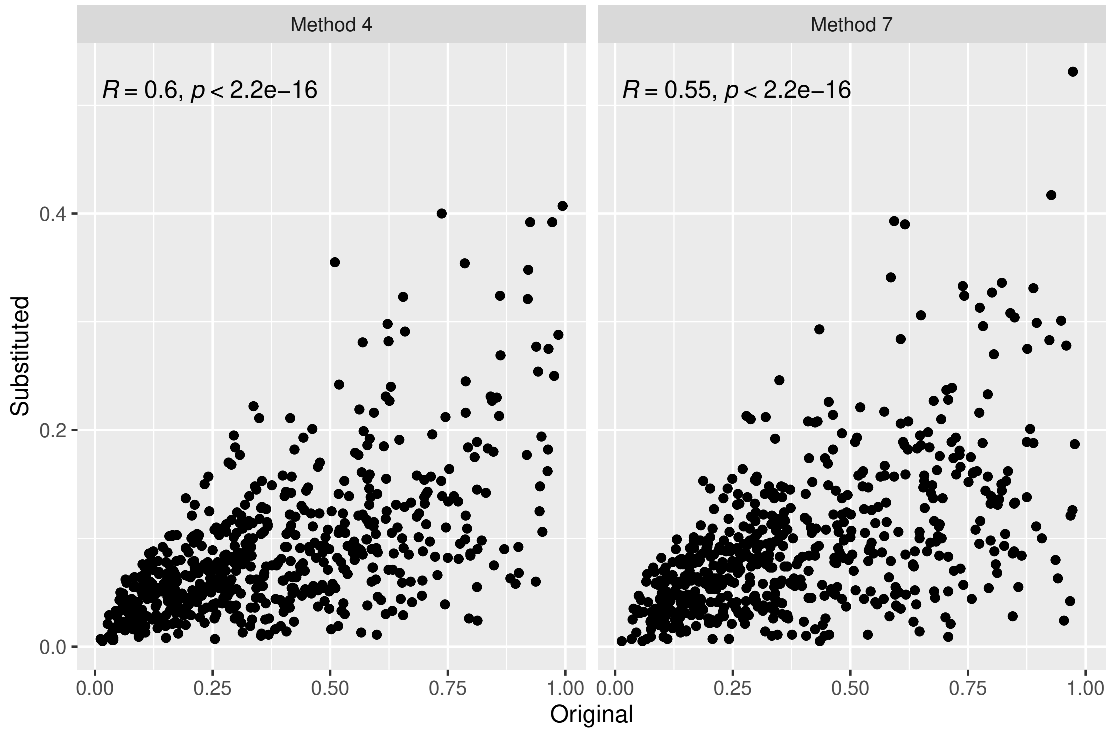

Figure S7 Scatter plot of the MitoFates prediction scores obtained for each individual synMTS from Methods 4 or 7 after appending Hac1p, using either the original design rules or substituting all of the arginines, except the final one involved in defining the mitochondrial peptidase cleavage site, for lysine. Each method contains 600 synMTS sequences. Spearman correlation coefficient was calculated using R version 4.4.2.

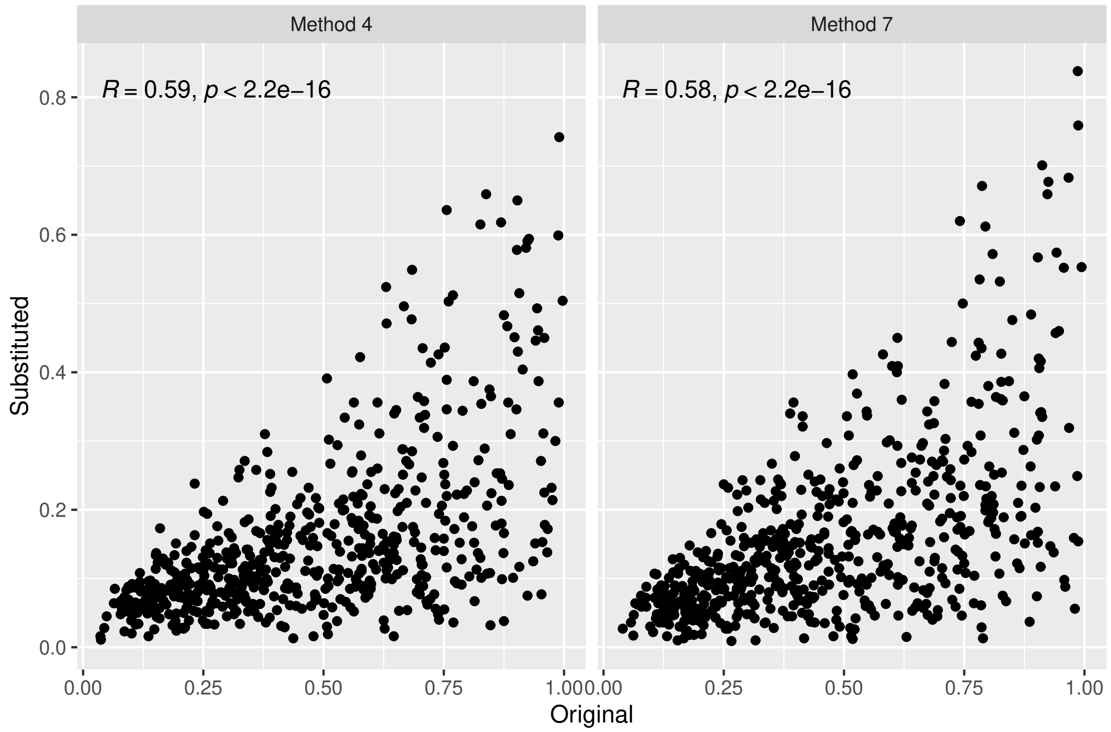

Figure S8 Scatter plot of the MitoFates prediction scores obtained for each individual synMTS from Methods 4 or 7 after appending Mmf1p, using either the original design rules or substituting all of the arginines, except the final one involved in defining the mitochondrial peptidase cleavage site, for lysine. Each method contains 600 synMTS sequences. Spearman correlation coefficient was calculated using R version 4.4.2.

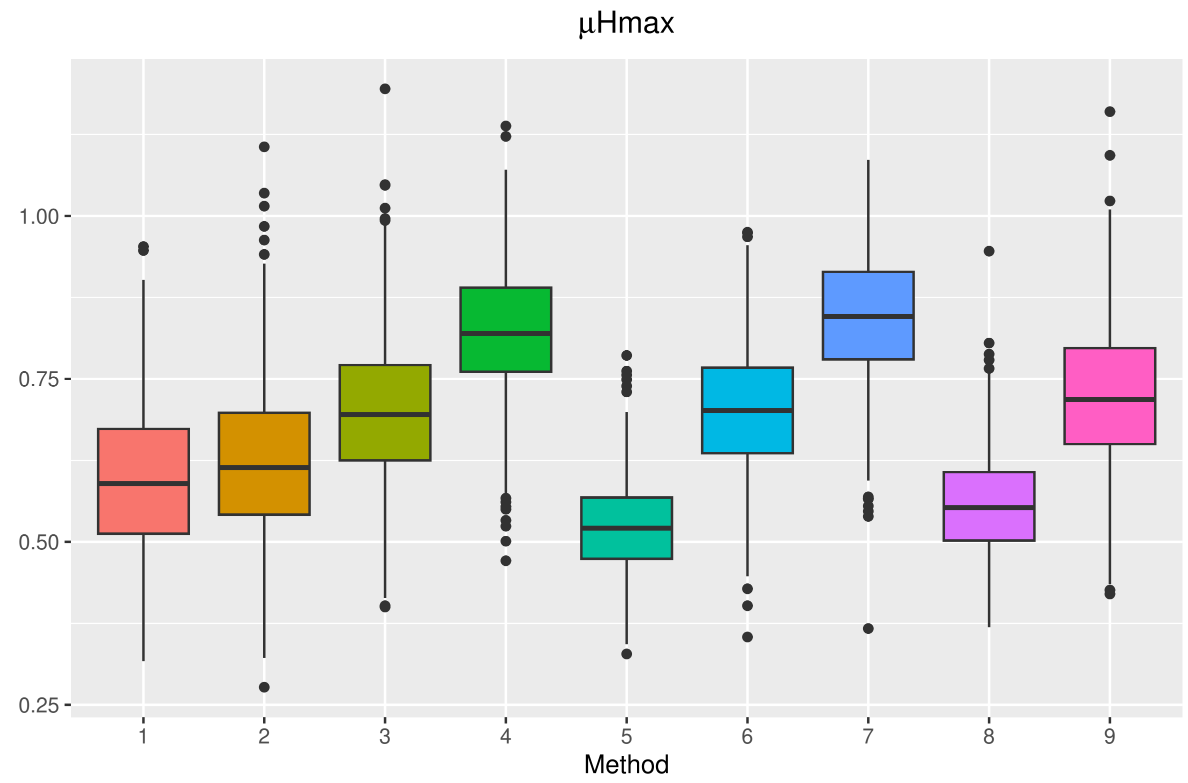

Figure S9 Box plots of the distribution of the maximum hydrophobic moments of each synMTS, across all of the methods.

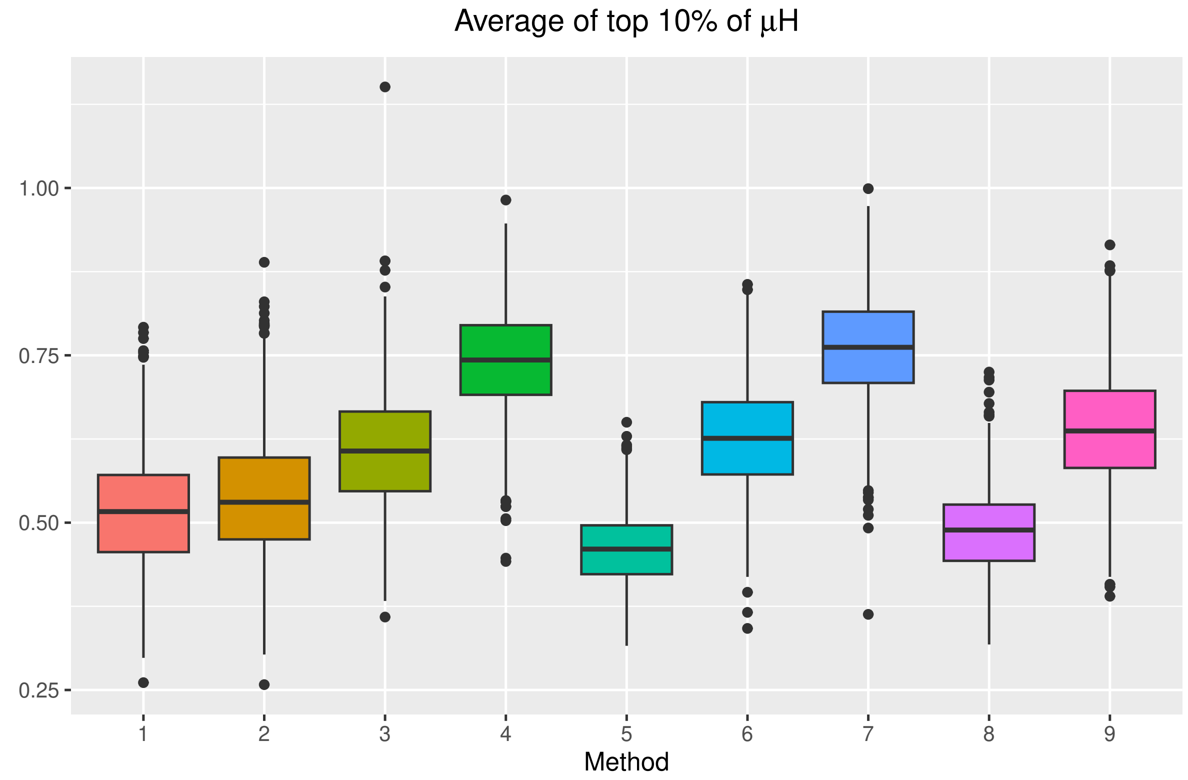

Figure S10 Box plots of the distribution of the average of the top 10% hydrophobic moments of each synMTS, across all of the methods.

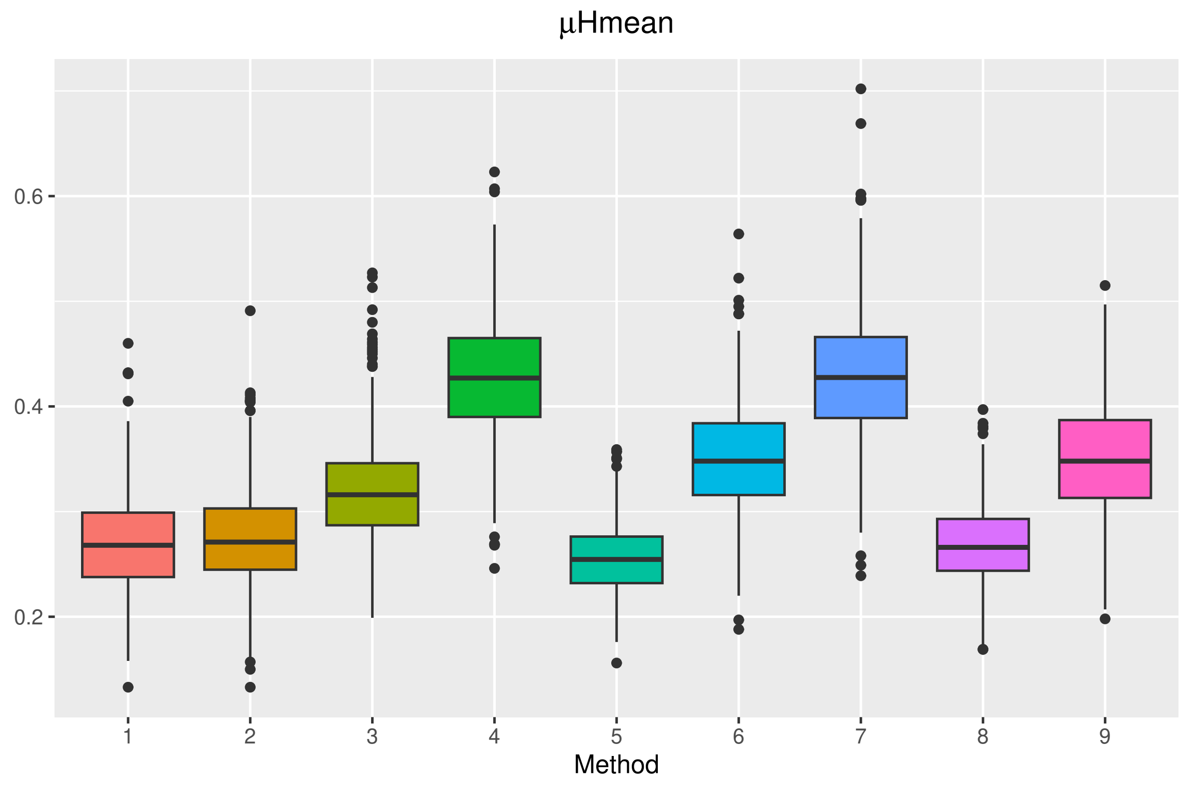

Figure S11 Box plots of the distribution of the average hydrophobic moments of each synMTS, across all of the methods.

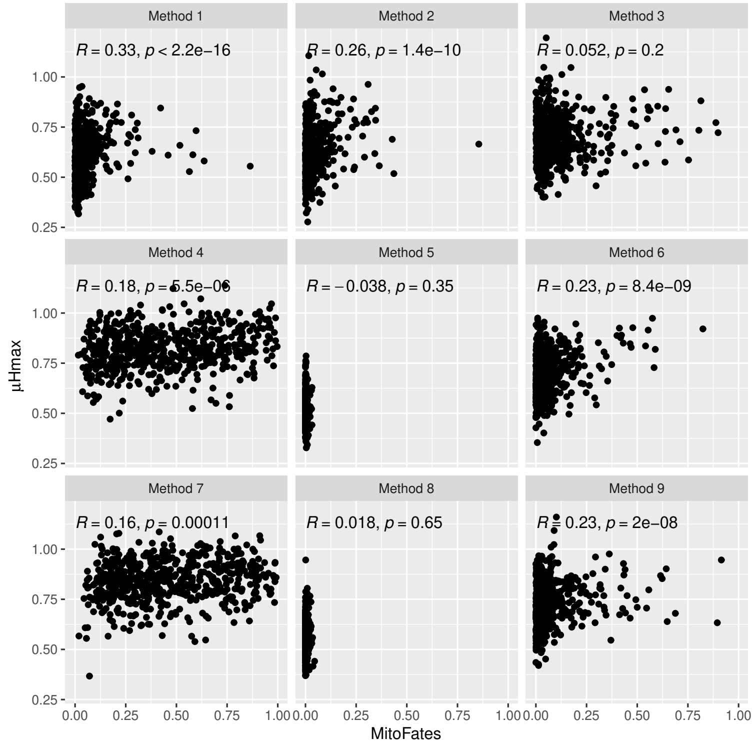

Figure S12 Scatter plot of maximum hydrophobic moment values against MitoFates prediction scores, with Cox2p-W56R appended to each synMTS. Each method has 600 synMTS sequences. The maximum hydrophobic moment values was calculated using hmoment, while Spearman correlation coefficient was calculated using R version 4.4.2.

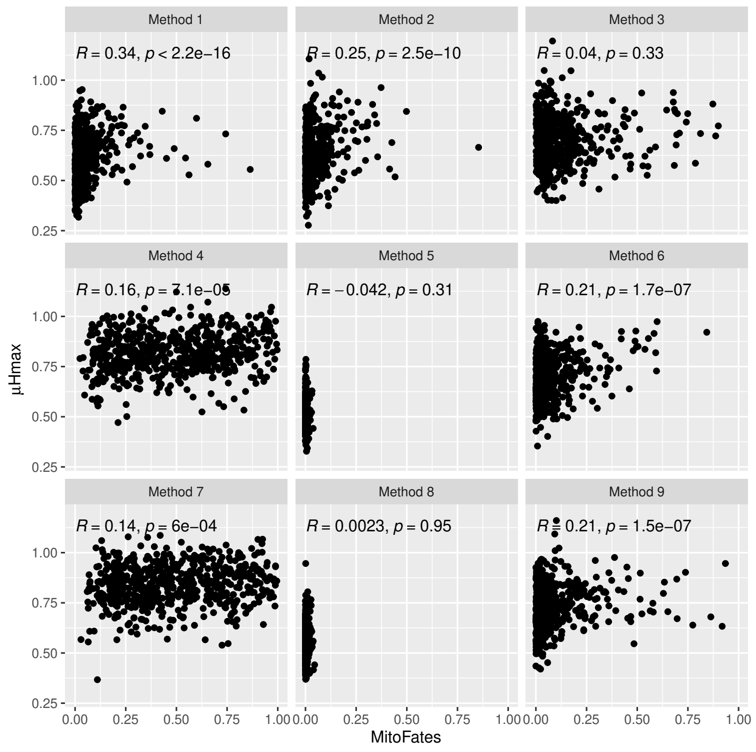

Figure S13 Scatter plot of maximum hydrophobic moment values against MitoFates prediction scores, with Atp9p appended to each synMTS. Each method has 600 synMTS sequences. The maximum hydrophobic moment values was calculated using hmoment, while Spearman correlation coefficient was calculated using R version 4.4.2.

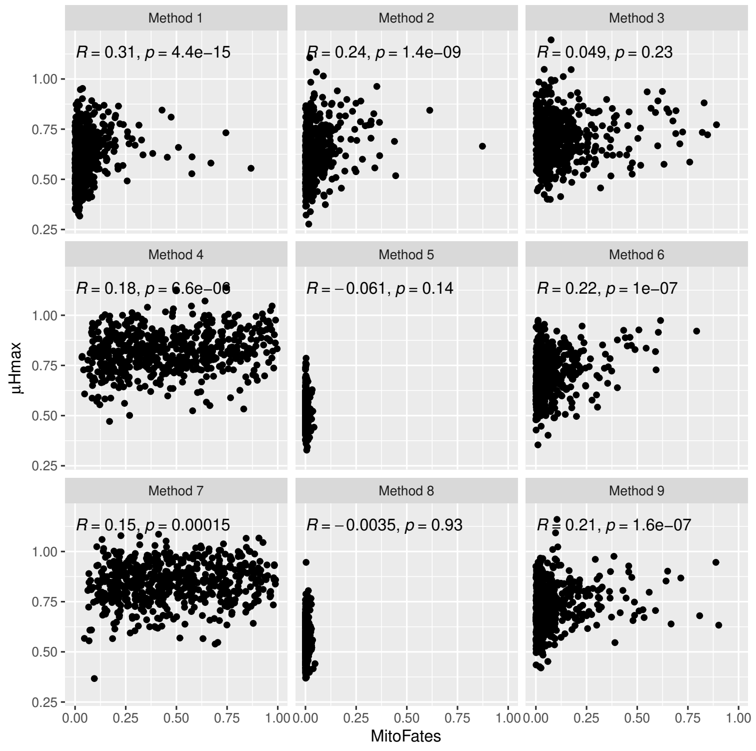

Figure S14 Scatter plot of maximum hydrophobic moment values against MitoFates prediction scores, with Atp8p appended to each synMTS. Each method has 600 synMTS sequences. The maximum hydrophobic moment values was calculated using hmoment, while Spearman correlation coefficient was calculated using R version 4.4.2.

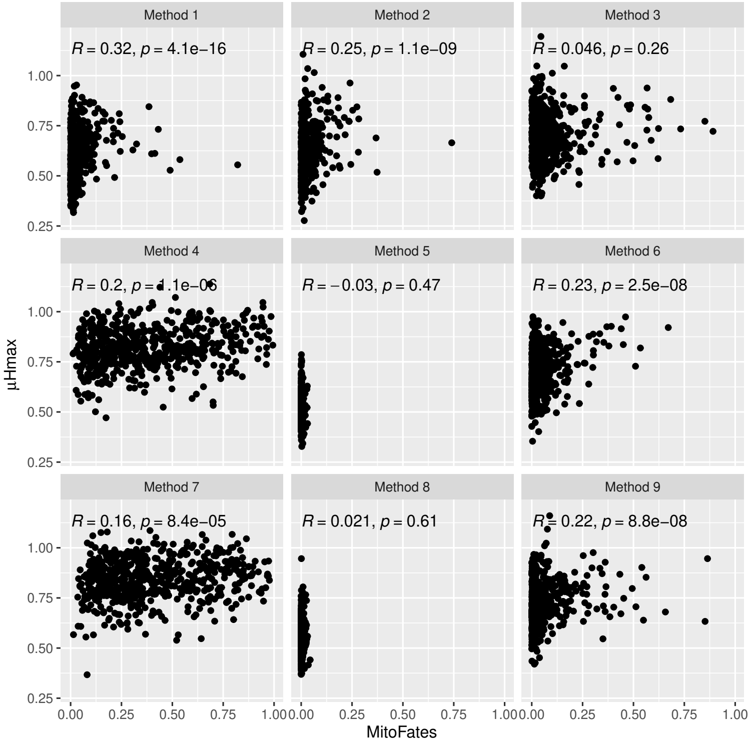

Figure S15 Scatter plot of maximum hydrophobic moment values against MitoFates prediction scores, with Hac1p appended to each synMTS. Each method has 600 synMTS sequences. The maximum hydrophobic moment values was calculated using hmoment, while Spearman correlation coefficient was calculated using R version 4.4.2.

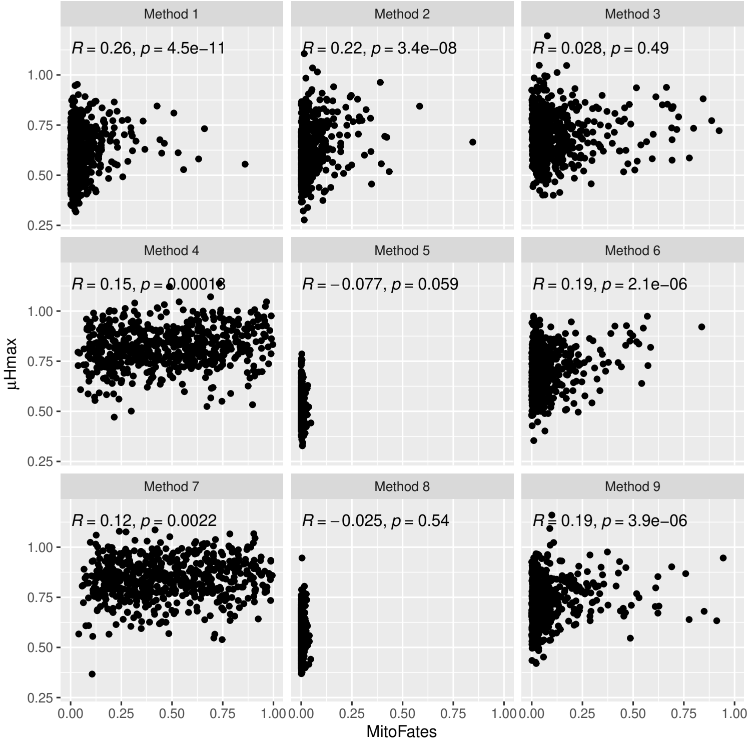

Figure S16 Scatter plot of maximum hydrophobic moment values against MitoFates prediction scores, with Mmf1p appended to each synMTS. Each method has 600 synMTS sequences. The maximum hydrophobic moment values was calculated using hmoment, while Spearman correlation coefficient was calculated using R version 4.4.2.

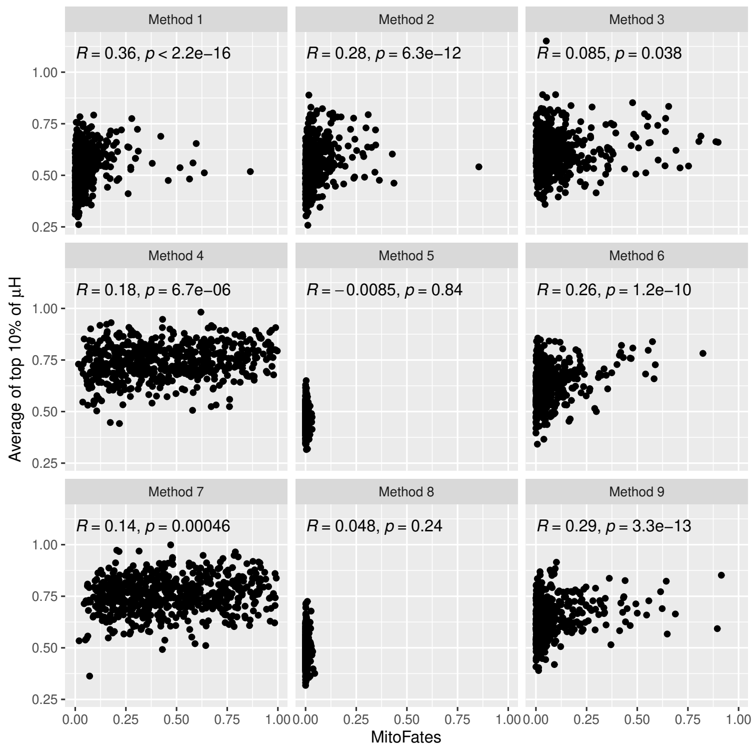

Figure S17 Scatter plot of average of the top 10% hydrophobic moment values against MitoFates prediction scores, with Cox2p-W56R appended to each synMTS. Each method has 600 synMTS sequences. The average of the top 10% hydrophobic moment values was calculated using hmoment, while Spearman correlation coefficient was calculated using R version 4.4.2.

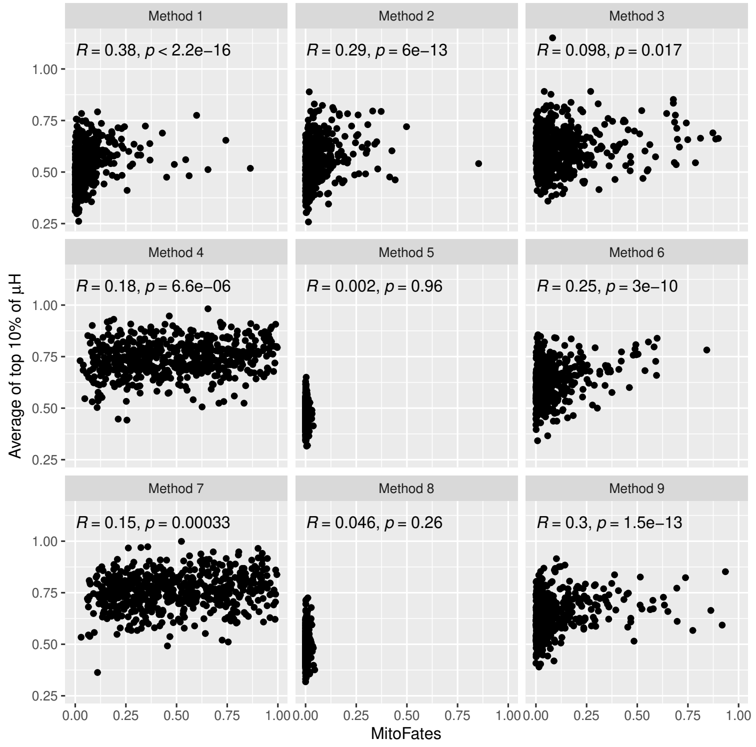

Figure S18 Scatter plot of average of the top 10% hydrophobic moment values against MitoFates prediction scores, with Atp9p appended to each synMTS. Each method has 600 synMTS sequences. The average of the top 10% hydrophobic moment values was calculated using hmoment, while Spearman correlation coefficient was calculated using R version 4.4.2.

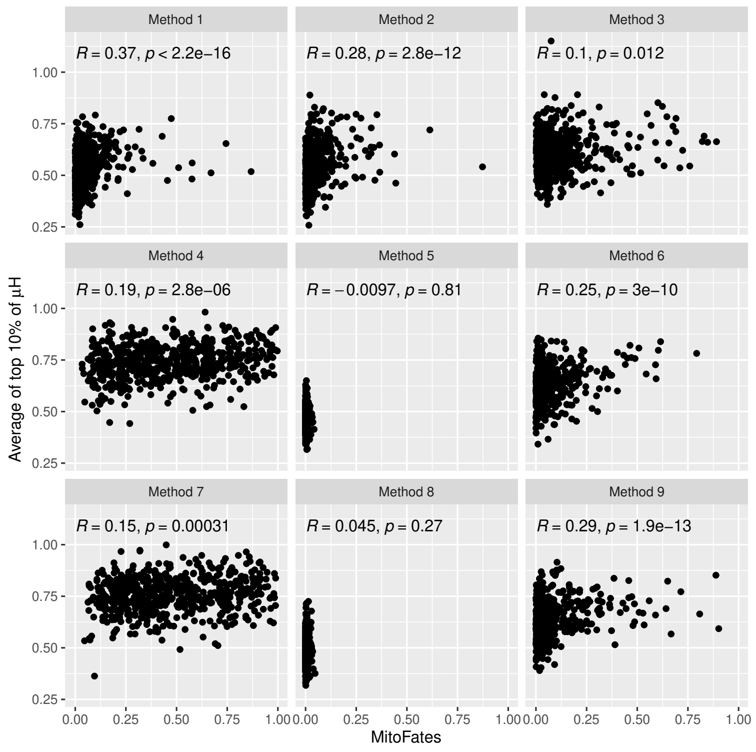

Figure S19 Scatter plot of average of the top 10% hydrophobic moment values against MitoFates prediction scores, with Atp8p appended to each synMTS. Each method has 600 synMTS sequences. The average of the top 10% hydrophobic moment values was calculated using hmoment, while Spearman correlation coefficient was calculated using R version 4.4.2.

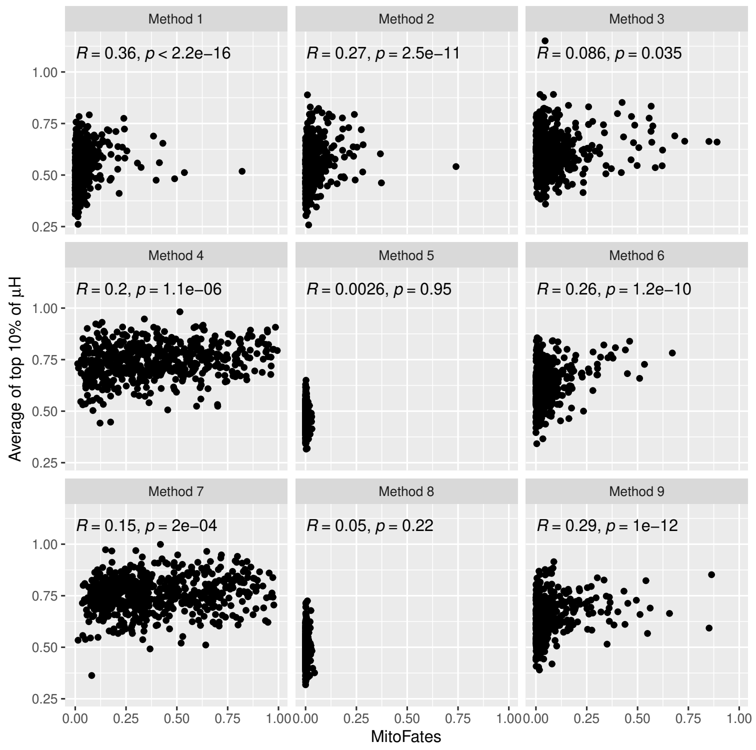

Figure S20 Scatter plot of average of the top 10% hydrophobic moment values against MitoFates prediction scores, with Hac1p appended to each synMTS. Each method has 600 synMTS sequences. The average of the top 10% hydrophobic moment values was calculated using hmoment, while Spearman correlation coefficient was calculated using R version 4.4.2.

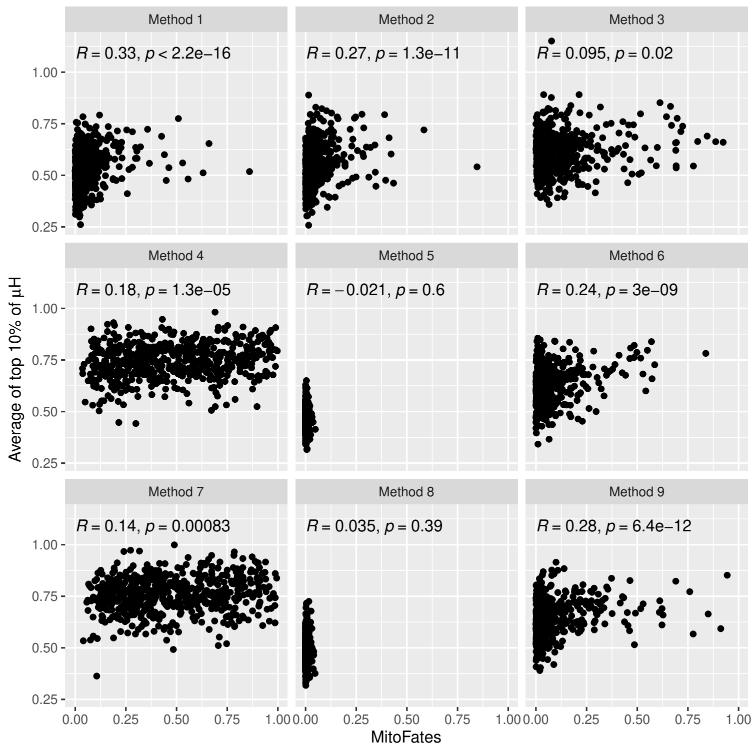

Figure S21 Scatter plot of average of the top 10% hydrophobic moment values against MitoFates prediction scores, with Mmf1p appended to each synMTS. Each method has 600 synMTS sequences. The average of the top 10% hydrophobic moment values was calculated using hmoment, while Spearman correlation coefficient was calculated using R version 4.4.2.

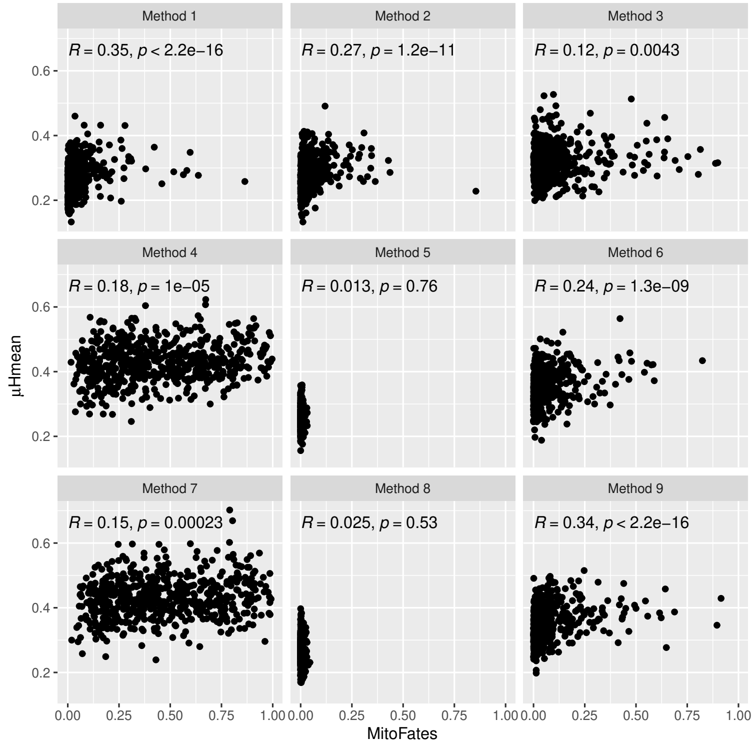

Figure S22 Scatter plot of average hydrophobic moment values against MitoFates prediction scores, with Cox2p-W56R appended to each synMTS. Each method has 600 synMTS sequences. The average hydrophobic moment values was calculated using hmoment, while Spearman correlation coefficient was calculated using R version 4.4.2.

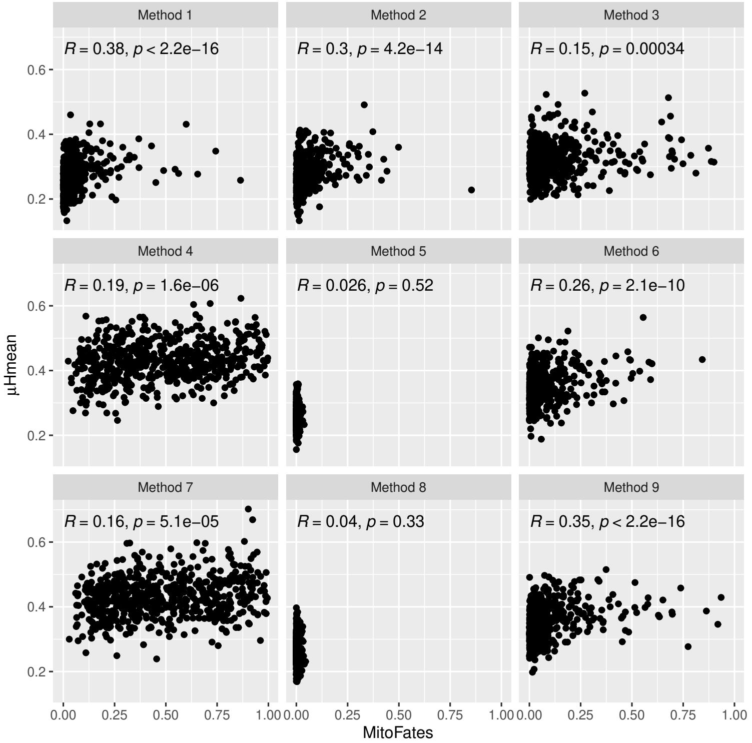

Figure S23 Scatter plot of average hydrophobic moment values against MitoFates prediction scores, with Atp9p appended to each synMTS. Each method has 600 synMTS sequences. The average hydrophobic moment values was calculated using hmoment, while Spearman correlation coefficient was calculated using R version 4.4.2.

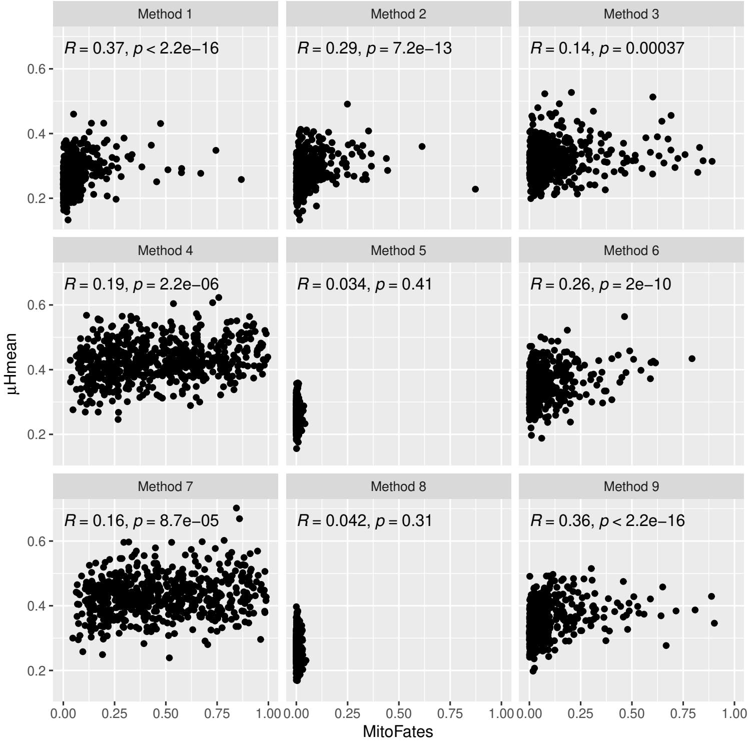

Figure S24 Scatter plot of average hydrophobic moment values against MitoFates prediction scores, with Atp8p appended to each synMTS. Each method has 600 synMTS sequences. The average hydrophobic moment values was calculated using hmoment, while Spearman correlation coefficient was calculated using R version 4.4.2.

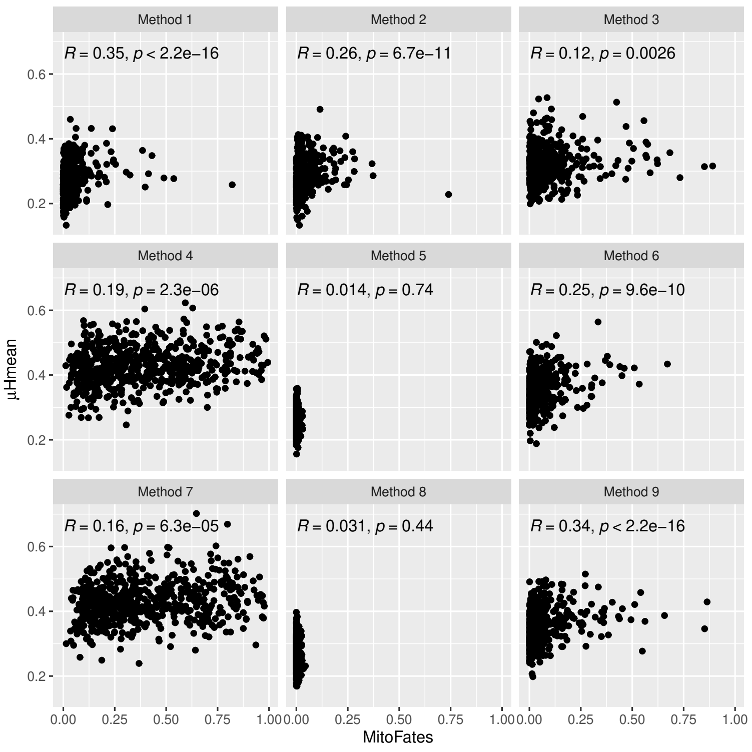

Figure S25 Scatter plot of average hydrophobic moment values against MitoFates prediction scores, with Hac1p appended to each synMTS. Each method has 600 synMTS sequences. The average hydrophobic moment values was calculated using hmoment, while Spearman correlation coefficient was calculated using R version 4.4.2.

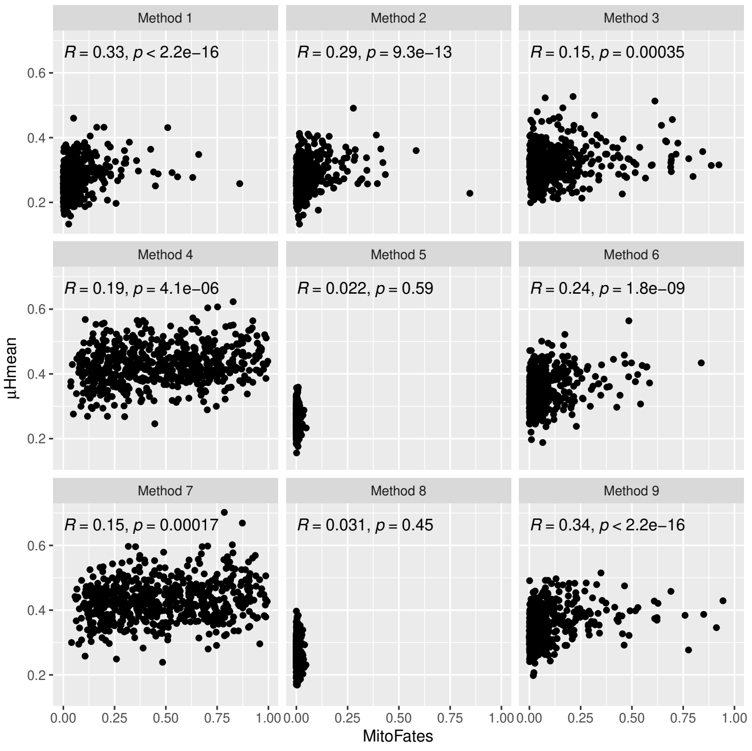

Figure S26 Scatter plot of average hydrophobic moment values against MitoFates prediction scores, with Mmf1p appended to each synMTS. Each method has 600 synMTS sequences. The average hydrophobic moment values was calculated using hmoment, while Spearman correlation coefficient was calculated using R version 4.4.2.

2.2. Functional validation of the selected synMTS

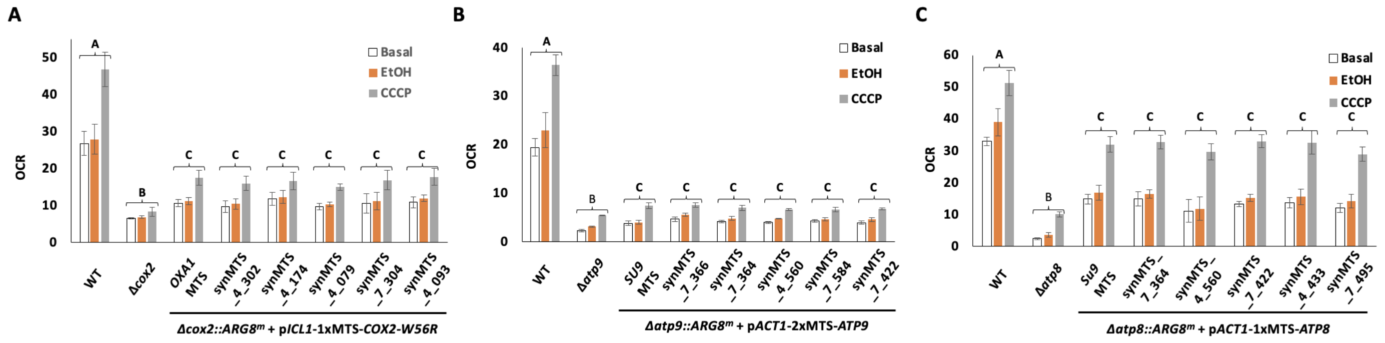

Figure S27 Measure of the oxygen consumption rates (OCR, nmol O2.min^{-1}.OD$_{600nm}$unit^{-1}, $n = 3$, mean +/- SD) on whole cells for the five best synthetic MTS for (A) *COX2-W56R*, (B) *ATP9*, and (C) *ATP8* allotopic expression systems identified in the first round of screening.

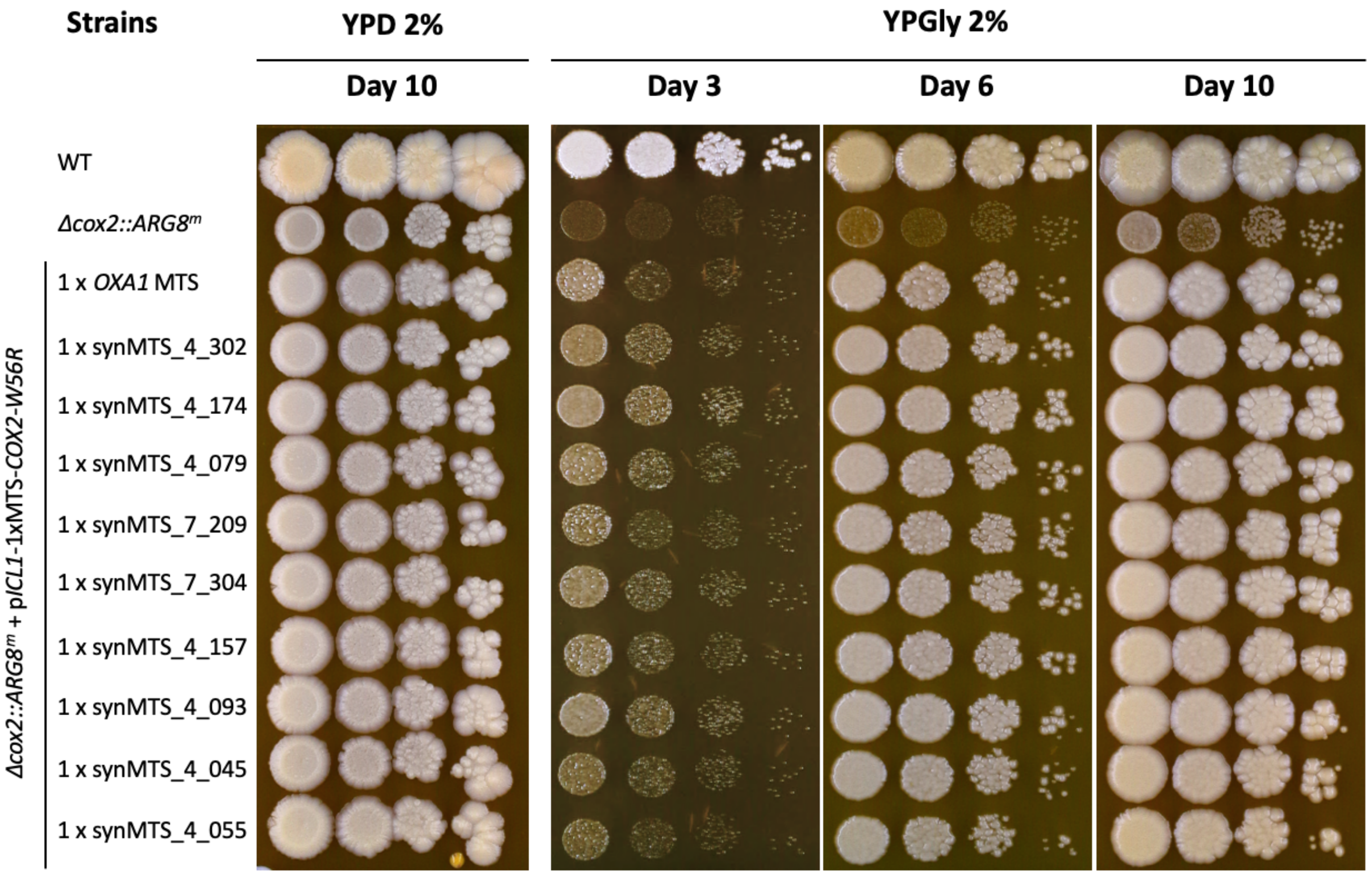

Figure S28 Spot-test testing the respiratory growth benefit conferred by a subset of nine synthetic MTS in the *COX2* deletion strain. The pictures were taken after incubating the plates for three, six, and ten days at 30°C.

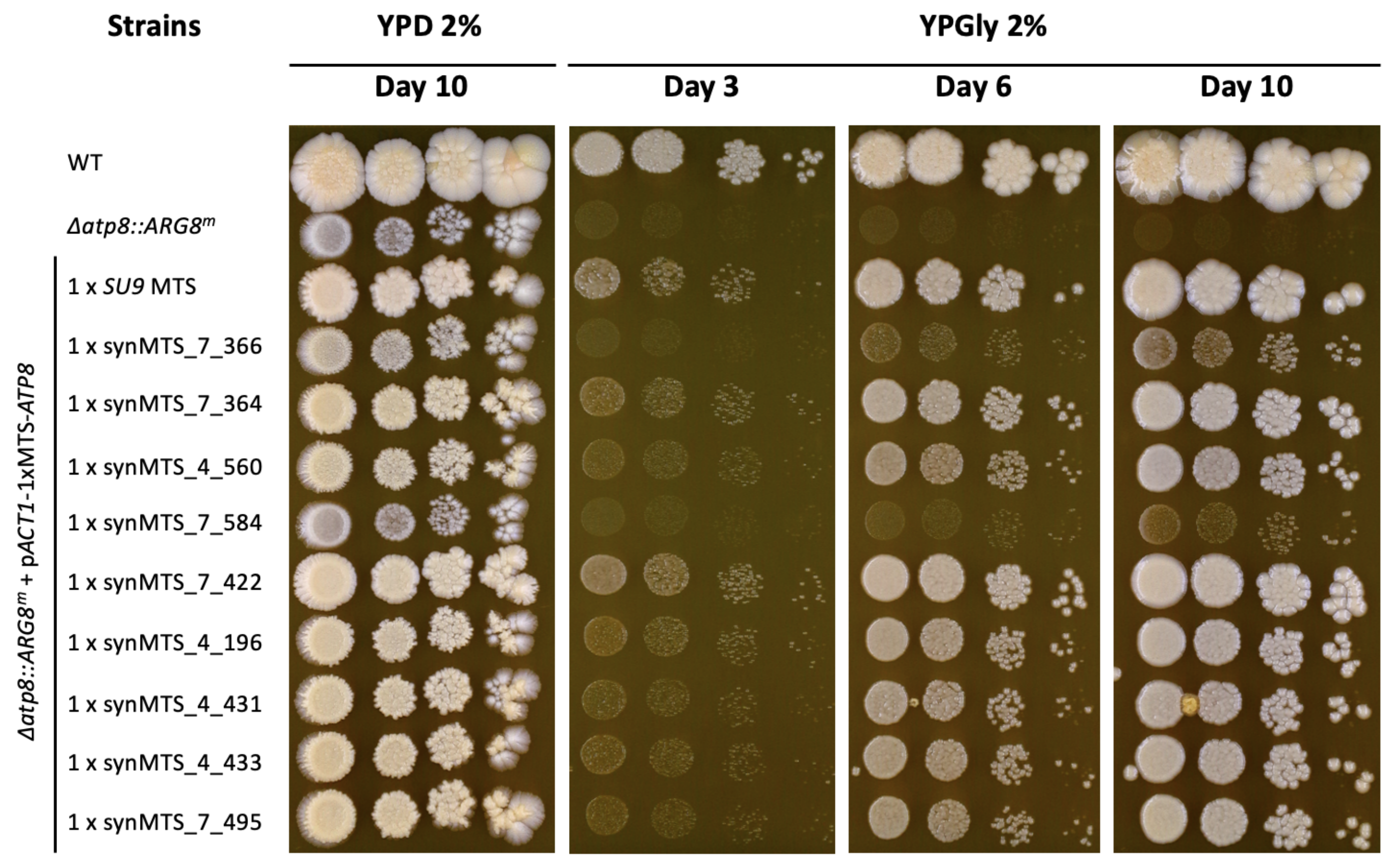

Figure S29 Spot-test testing the respiratory growth benefit conferred by a subset of nine synthetic MTS in the *ATP8* deletion strain. The pictures were taken after incubating the plates for three, six, and ten days at 30°C.

synMTS ID	Cherry	IQ-C	Cox4p	Cox2p-W56R	Atp9p	Atp8p
synMTS_4_029	++	25	++	-	-	-
synMTS_4_045	++	51	++	++	N/A	+
synMTS_4_055	++	37	++	++	N/A	-
synMTS_4_078	+	35	++	-	-	-
synMTS_4_079	++	41	++	++	N/A	-
synMTS_4_081	++	25	++	++	-	-
synMTS_4_082	++	35	++	-	N/A	++
synMTS_4_093	++	25	++	++	N/A	++
synMTS_4_098	+	37	++	-	N/A	-
synMTS_4_106	+	19	++	-	-	+
synMTS_4_122	++	24	++	++	-	-
synMTS_4_126	++	25	++	++	-	-
synMTS_4_157	++	46	++	++	N/A	++
synMTS_4_174	++	40	++	++	N/A	-
synMTS_4_196	++	34	++	-	N/A	++
synMTS_4_218	++	31	++	++	N/A	++
synMTS_4_229	++	34	++	-	N/A	-
synMTS_4_302	++	51	++	++	N/A	-
synMTS_4_308	++	18	++	++	N/A	-
synMTS_4_333	++	28	++	++	N/A	-
synMTS_4_361	++	33	++	++	N/A	+
synMTS_4_411	+	37	-	-	-	-
synMTS_4_431	++	38	++	++	N/A	++
synMTS_4_433	-	37	++	++	N/A	++
synMTS_4_472	++	26	++	++	N/A	++
synMTS_4_530	+	33	-	-	N/A	-
synMTS_4_560	++	44	++	+	++	++
synMTS_4_562	-	28	++	++	N/A	-
synMTS_7_027	-	27	++	-	N/A	+
synMTS_7_053	-	28	-	++	-	-
synMTS_7_089	++	24	++	++	-	-
synMTS_7_091	+	31	++	+	-	-
synMTS_7_106	+	36	+	++	-	-
synMTS_7_136	++	40	++	++	-	-
synMTS_7_173	++	48	++	-	-	-
synMTS_7_209	++	47	++	++	N/A	+
synMTS_7_210	++	46	++	++	N/A	-
synMTS_7_269	++	11	++	-	N/A	++
synMTS_7_304	++	42	++	++	N/A	++
synMTS_7_325	+	41	+	-	-	-
synMTS_7_364	++	45	++	++	++	++
synMTS_7_366	-	58	++	-	++	-
synMTS_7_386	++	32	++	+	N/A	++
synMTS_7_422	++	50	++	++	++	++
synMTS_7_430	++	7	++	-	N/A	+
synMTS_7_495	+	41	-	+	N/A	++
synMTS_7_527	+	42	++	-	-	+
synMTS_7_535	++	37	+	+	N/A	++
synMTS_7_547	+	34	+	-	N/A	-
synMTS_7_584	-	28	++	-	++	-
++	33		42	27	5	16
+	11		4	5	0	7
-	6		4	18	15	27
N/A	0		0	0	30	0

Table S11 Summary of the visual assessments of each synMTS with various passenger proteins. “++” indicates a satisfactory addressing to mitochondria or restoration of the respiratory growth of the deletion mutant, while “+” indicates a partial, but not satisfactory observation. A “-” indicates that the presequence is not functional. A “N/A” indicates that this synMTS has not been tested for the corresponding deletion mutant.

	Positive (mean ± SD)	Negative (mean ± SD)	p value
n	27	18	
Length	84.963 ± 22.229	75.167 ± 24.853	0.186
charge_sum	20.707 ± 5.639	18.872 ± 6.660	0.343
krcount_sum	20.889 ± 5.679	18.889 ± 6.498	0.296
krcount_avg	0.245 ± 0.012	0.251 ± 0.016	0.193
MitoFates	0.499 ± 0.235	0.448 ± 0.210	0.452
DeepMito	0.887 ± 0.033	0.872 ± 0.059	0.356
μHmax	0.816 ± 0.092	0.852 ± 0.104	0.241
Average of top 10% of μH	0.742 ± 0.079	0.771 ± 0.084	0.264
μHmean	0.412 ± 0.049	0.440 ± 0.053	0.077

Table S12 Properties of the synMTS sequences for the Cox2p-W56R passenger protein, divided into positive (++) and negative (-) data sets, according to Table S11. The synMTS that were labeled as + or "N/A" were determined to be inclusive and thus removed from consideration. Student's t-test were applied between the two data sets, with the *p* value reported in the last column..

	Positive (mean ± SD)	Negative (mean ± SD)	p value
n	5	15	
Length	59.000 ± 14.883	86.067 ± 24.479	0.012
charge_sum	15.160 ± 3.908	21.480 ± 6.873	0.025
krcount_sum	15.200 ± 3.701	21.600 ± 6.759	0.019
krcount_avg	0.258 ± 0.008	0.250 ± 0.016	0.147
MitoFates	0.717 ± 0.168	0.464 ± 0.263	0.029
DeepMito	0.960 ± 0.000	0.879 ± 0.061	0.000
μHmax	0.827 ± 0.075	0.867 ± 0.105	0.377
Average of top 10% of μH	0.764 ± 0.069	0.776 ± 0.089	0.777
μHmean	0.443 ± 0.032	0.431 ± 0.060	0.578

Table S13 Properties of the synMTS sequences for the Atp9p passenger protein, divided into positive (++) and negative (-) data sets, according to Table S11. The synMTS that were labeled as + or "N/A" were determined to be inclusive and thus removed from consideration. Student's t-test were applied between the two data sets, with the *p* value reported in the last column..

	Positive (mean ± SD)	Negative (mean ± SD)	p value
n	16	27	
Length	82.875 ± 24.342	83.148 ± 24.695	0.972
charge_sum	20.294 ± 6.210	20.752 ± 6.596	0.821
krcount_sum	20.375 ± 6.228	20.852 ± 6.509	0.813
krcount_avg	0.245 ± 0.013	0.250 ± 0.016	0.284
MitoFates	0.489 ± 0.264	0.453 ± 0.208	0.645
DeepMito	0.899 ± 0.049	0.890 ± 0.052	0.584
μHmax	0.810 ± 0.075	0.840 ± 0.106	0.289
Average of top 10% of μH	0.736 ± 0.073	0.758 ± 0.080	0.361
μHmean	0.413 ± 0.040	0.428 ± 0.060	0.352

Table S14 Properties of the synMTS sequences for the Atp8p passenger protein, divided into positive (++) and negative (-) data sets, according to Table S11. The synMTS that were labeled as + or "N/A" were determined to be inclusive and thus removed from consideration. Student's t-test were applied between the two data sets, with the *p* value reported in the last column..

synMTS ID	Cargo	Phenotype	N6
synMTS_4_029	Cox2p-W56R	-	MFMMVK
synMTS_4_045	Cox2p-W56R	++	MGVRWM
synMTS_4_055	Cox2p-W56R	++	MHNRYQ
synMTS_4_078	Cox2p-W56R	-	MLNTNK
synMTS_4_079	Cox2p-W56R	++	MLQKVF
synMTS_4_081	Cox2p-W56R	++	MLYCKL
synMTS_4_082	Cox2p-W56R	-	MLYRYY
synMTS_4_093	Cox2p-W56R	++	MNCRVA
synMTS_4_098	Cox2p-W56R	-	MNNYKM
synMTS_4_106	Cox2p-W56R	-	MNVWRF
synMTS_4_122	Cox2p-W56R	++	MQIHPR
synMTS_4_126	Cox2p-W56R	++	MQSHQK
synMTS_4_157	Cox2p-W56R	++	MTISTK
synMTS_4_174	Cox2p-W56R	++	MVYRPW
synMTS_4_196	Cox2p-W56R	-	MYVWKC
synMTS_4_218	Cox2p-W56R	++	MFMFNK
synMTS_4_229	Cox2p-W56R	-	MGGRFT
synMTS_4_302	Cox2p-W56R	++	MMTRVC
synMTS_4_308	Cox2p-W56R	++	MNIRLW
synMTS_4_333	Cox2p-W56R	++	MQARIF
synMTS_4_361	Cox2p-W56R	++	MTCWRW
synMTS_4_411	Cox2p-W56R	-	MCGTKH
synMTS_4_431	Cox2p-W56R	++	MGLVRI
synMTS_4_433	Cox2p-W56R	++	MGPMTK
synMTS_4_472	Cox2p-W56R	++	MLIKPQ
synMTS_4_530	Cox2p-W56R	-	MQSYHR
synMTS_4_562	Cox2p-W56R	++	MVSRQW
synMTS_7_027	Cox2p-W56R	-	MFCLKI
synMTS_7_053	Cox2p-W56R	++	MHGGRC
synMTS_7_089	Cox2p-W56R	++	MLYCRS
synMTS_7_106	Cox2p-W56R	++	MNIQVR
synMTS_7_136	Cox2p-W56R	++	MQFRVP
synMTS_7_173	Cox2p-W56R	-	MVAKCA
synMTS_7_209	Cox2p-W56R	++	MAIKWN
synMTS_7_210	Cox2p-W56R	++	MAMRPF
synMTS_7_269	Cox2p-W56R	-	MIVKLN
synMTS_7_304	Cox2p-W56R	++	MNVPTK
synMTS_7_325	Cox2p-W56R	-	MQHYHK
synMTS_7_364	Cox2p-W56R	++	MVIRTM
synMTS_7_366	Cox2p-W56R	-	MVQAKT
synMTS_7_422	Cox2p-W56R	++	MFSRFT
synMTS_7_430	Cox2p-W56R	-	MGNHIK
synMTS_7_527	Cox2p-W56R	-	MQSRHA
synMTS_7_547	Cox2p-W56R	-	MTFNRS
synMTS_7_584	Cox2p-W56R	-	MWTMRV
synMTS_4_078	Atp9p	-	MLNTNK
synMTS_4_081	Atp9p	-	MLYCKL
synMTS_4_106	Atp9p	-	MNVWRF
synMTS_4_122	Atp9p	-	MQIHPR
synMTS_4_126	Atp9p	-	MQSHQK
synMTS_4_411	Atp9p	-	MCGTKH
synMTS_4_560	Atp9p	++	MVLRSH
synMTS_7_053	Atp9p	-	MHGGRC
synMTS_7_089	Atp9p	-	MLYCRS
synMTS_7_091	Atp9p	-	MMCKLY
synMTS_7_106	Atp9p	-	MNIQVR

Continued on next page

Table S15 – Continued from previous page

synMTS ID	Cargo	Phenotype	N6
synMTS_7_136	Atp9p	-	MQFRVP
synMTS_7_173	Atp9p	-	MVAKCA
synMTS_7_325	Atp9p	-	MQHYHK
synMTS_7_364	Atp9p	++	MVIRTM
synMTS_7_366	Atp9p	++	MVQAKT
synMTS_7_422	Atp9p	++	MFSRFT
synMTS_7_495	Atp9p	-	MNSRIH
synMTS_7_527	Atp9p	-	MQSRHA
synMTS_7_584	Atp9p	++	MWTMRV
synMTS_4_029	Atp8p	-	MFMMVK
synMTS_4_055	Atp8p	-	MHNRYQ
synMTS_4_078	Atp8p	-	MLNTNK
synMTS_4_079	Atp8p	-	MLQKVF
synMTS_4_081	Atp8p	-	MLYCKL
synMTS_4_082	Atp8p	++	MLYRYY
synMTS_4_093	Atp8p	++	MNCRVA
synMTS_4_098	Atp8p	-	MNNYKM
synMTS_4_122	Atp8p	-	MQIHPR
synMTS_4_126	Atp8p	-	MQSHQK
synMTS_4_157	Atp8p	++	MTISTK
synMTS_4_174	Atp8p	-	MVYRPW
synMTS_4_196	Atp8p	++	MYVWKC
synMTS_4_218	Atp8p	++	MFMFNK
synMTS_4_229	Atp8p	-	MGGRFT
synMTS_4_302	Atp8p	-	MMTRVC
synMTS_4_308	Atp8p	-	MNIRLW
synMTS_4_333	Atp8p	-	MQARIF
synMTS_4_411	Atp8p	-	MCGTKH
synMTS_4_431	Atp8p	++	MGLVRI
synMTS_4_433	Atp8p	++	MGPMTK
synMTS_4_472	Atp8p	++	MLIKPQ
synMTS_4_530	Atp8p	-	MQSYHR
synMTS_4_560	Atp8p	++	MVLRSH
synMTS_4_562	Atp8p	-	MVSRQW
synMTS_7_053	Atp8p	-	MHGGRC
synMTS_7_089	Atp8p	-	MLYCRS
synMTS_7_091	Atp8p	-	MMCKLY
synMTS_7_106	Atp8p	-	MNIQVR
synMTS_7_136	Atp8p	-	MQFRVP
synMTS_7_173	Atp8p	-	MVAKCA
synMTS_7_210	Atp8p	-	MAMRPF
synMTS_7_269	Atp8p	++	MIVKLN
synMTS_7_304	Atp8p	++	MNVPTK
synMTS_7_325	Atp8p	-	MQHYHK
synMTS_7_364	Atp8p	++	MVIRTM
synMTS_7_366	Atp8p	-	MVQAKT
synMTS_7_386	Atp8p	++	MWTHVR
synMTS_7_422	Atp8p	++	MFSRFT
synMTS_7_495	Atp8p	++	MNSRIH
synMTS_7_535	Atp8p	++	MSINNR
synMTS_7_547	Atp8p	-	MTFNRS
synMTS_7_584	Atp8p	-	MWTMRV

Table S15: List of the synMTS sequences used for motifs discovery analysis and the generation of the N6 logos based on the respiratory phenotypes obtained for the different spot-tests.

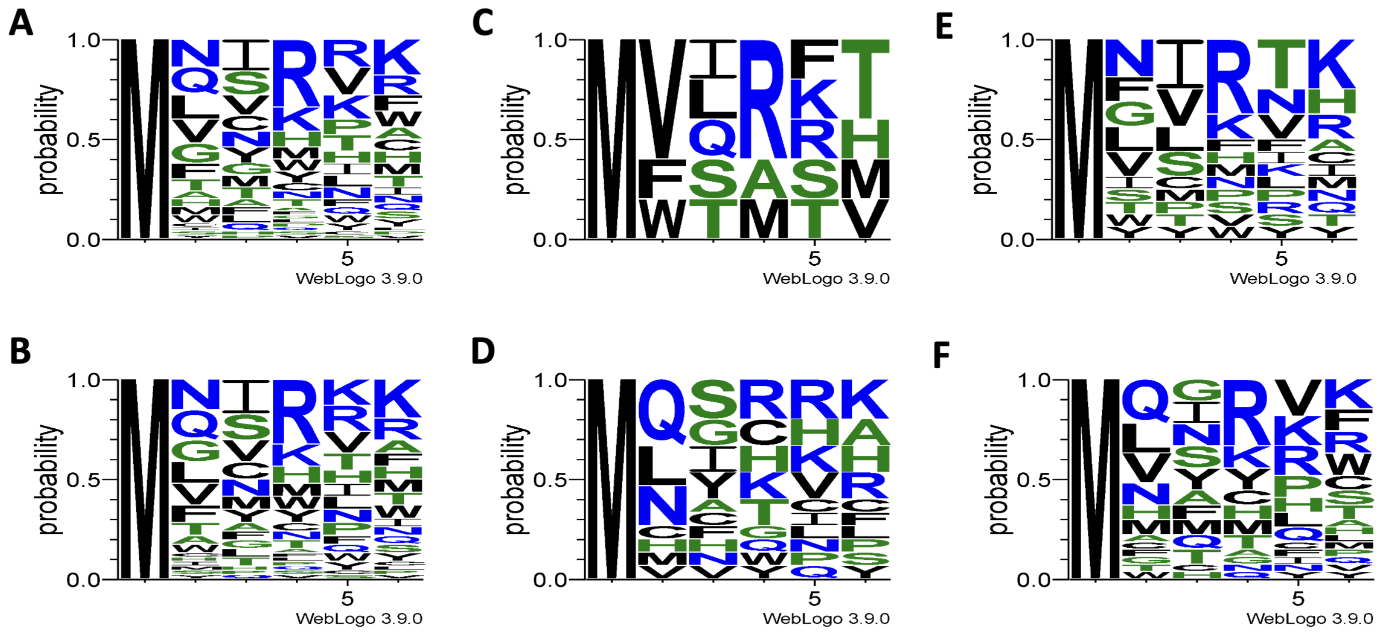

Figure S30 Analysis of the composition of the first six N-terminal residues as described by Nashed and collaborators (6). We focused this analysis on the functional presequences (Phenotype "++") versus non-functional presequences (Phenotype "-") as described in Table S12. The six first residues were extracted and submitted to WebLogo 3.9.0 web site (3). The returned logos are presenting the motifs obtained for Cox2p-W56R (A= function, B= non-functional), Atp9p (C= function, D= non-functional) and Atp8p (E= function, F= non-functional).

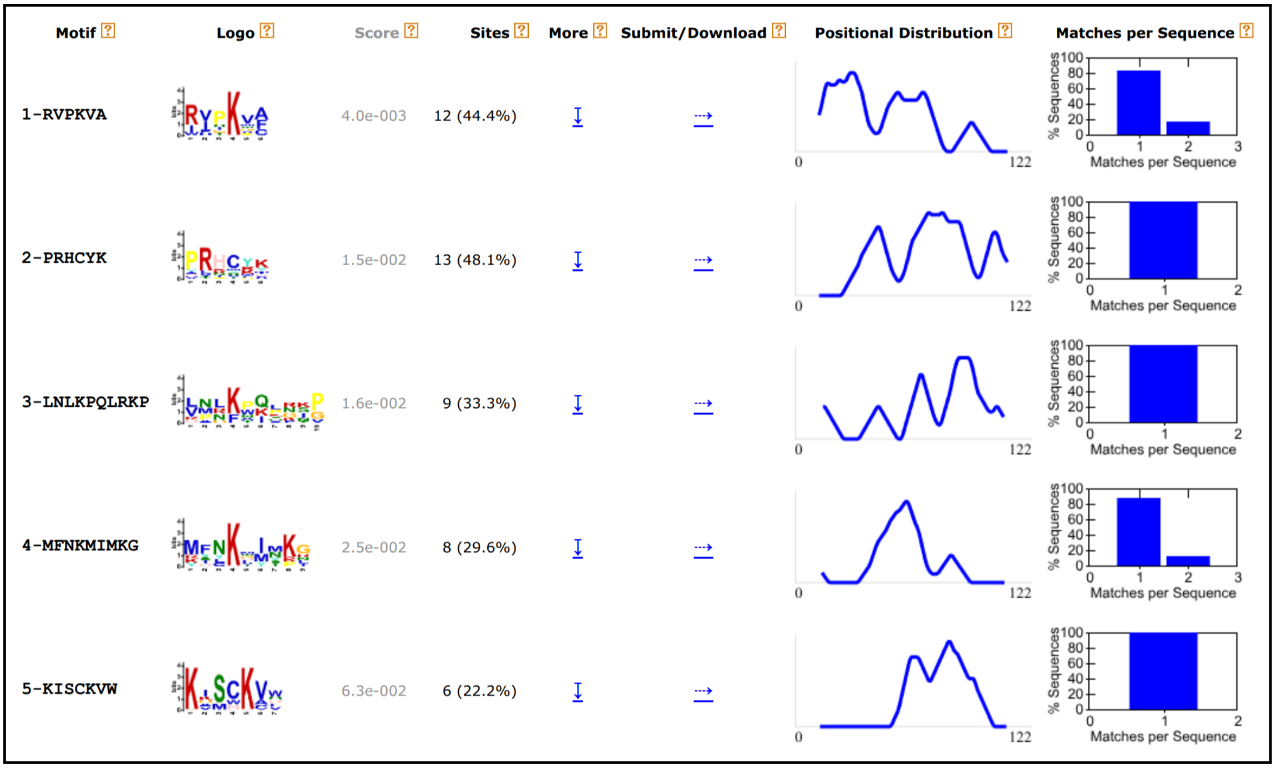

Figure S31 Screen shot from ungapped motif discovery using Streme (1) and the usual amino acids for the passenger protein Cox2p-W56R.

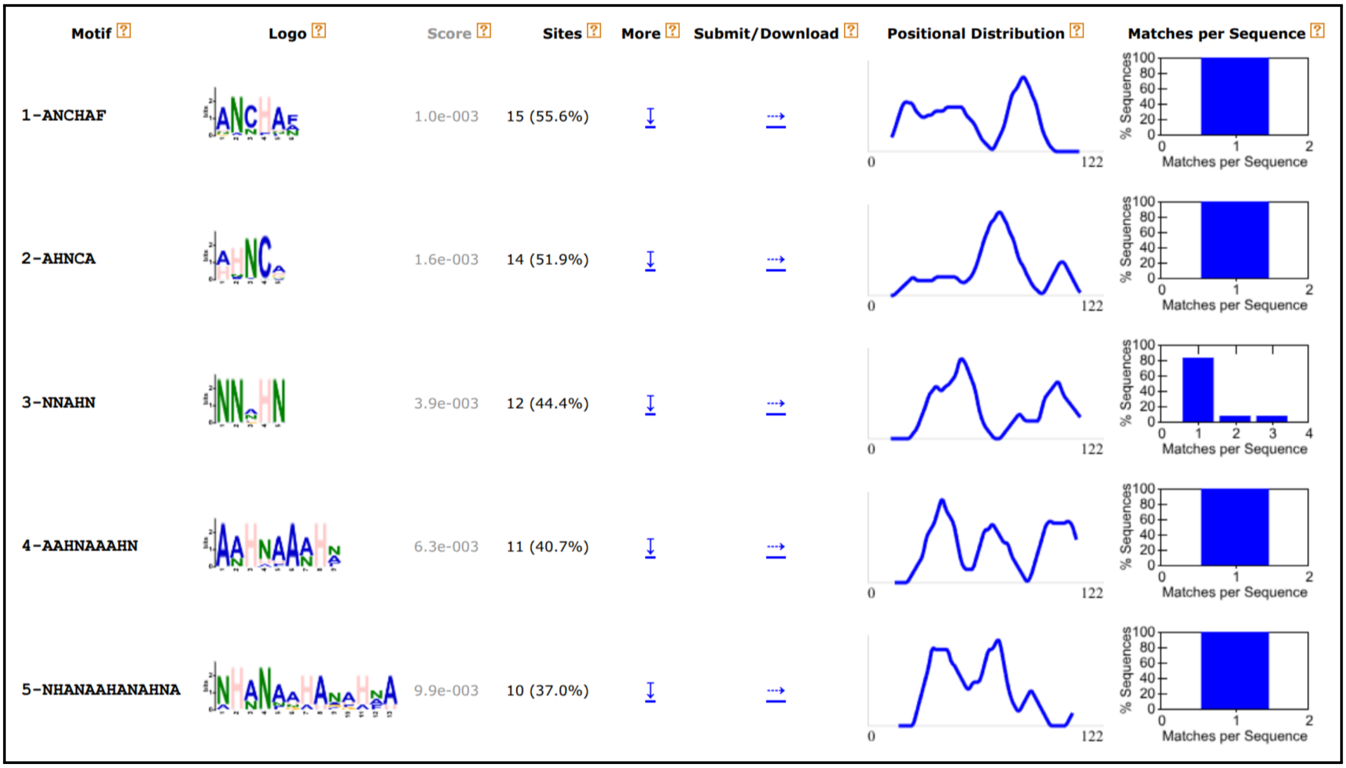

Figure S32 Screen shot from ungapped motif discovery using Streme (1) and amino acid categories of Table S10 for the passenger protein Cox2p-W56R.

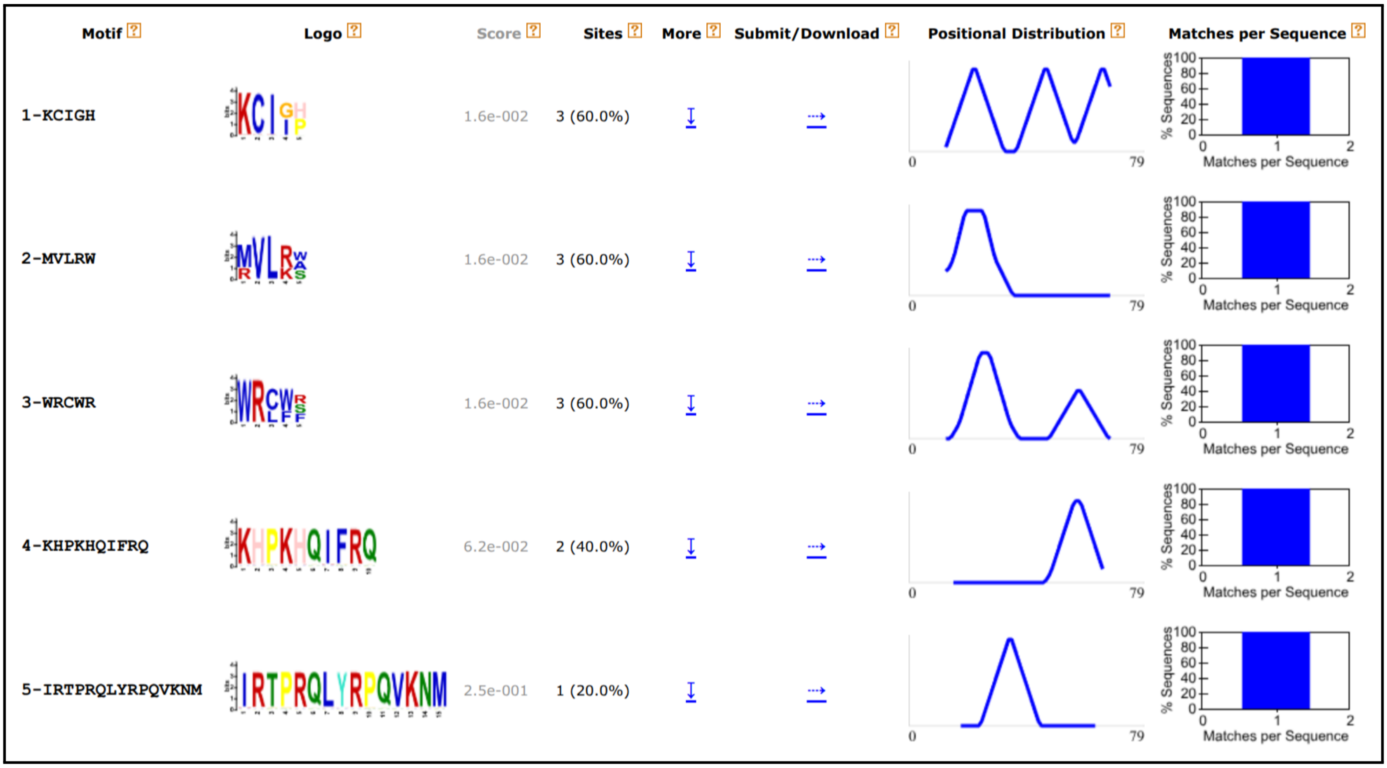

Figure S33 Screen shot from ungapped motif discovery using Streme (1) and the usual amino acids for the passenger protein Atp9p.

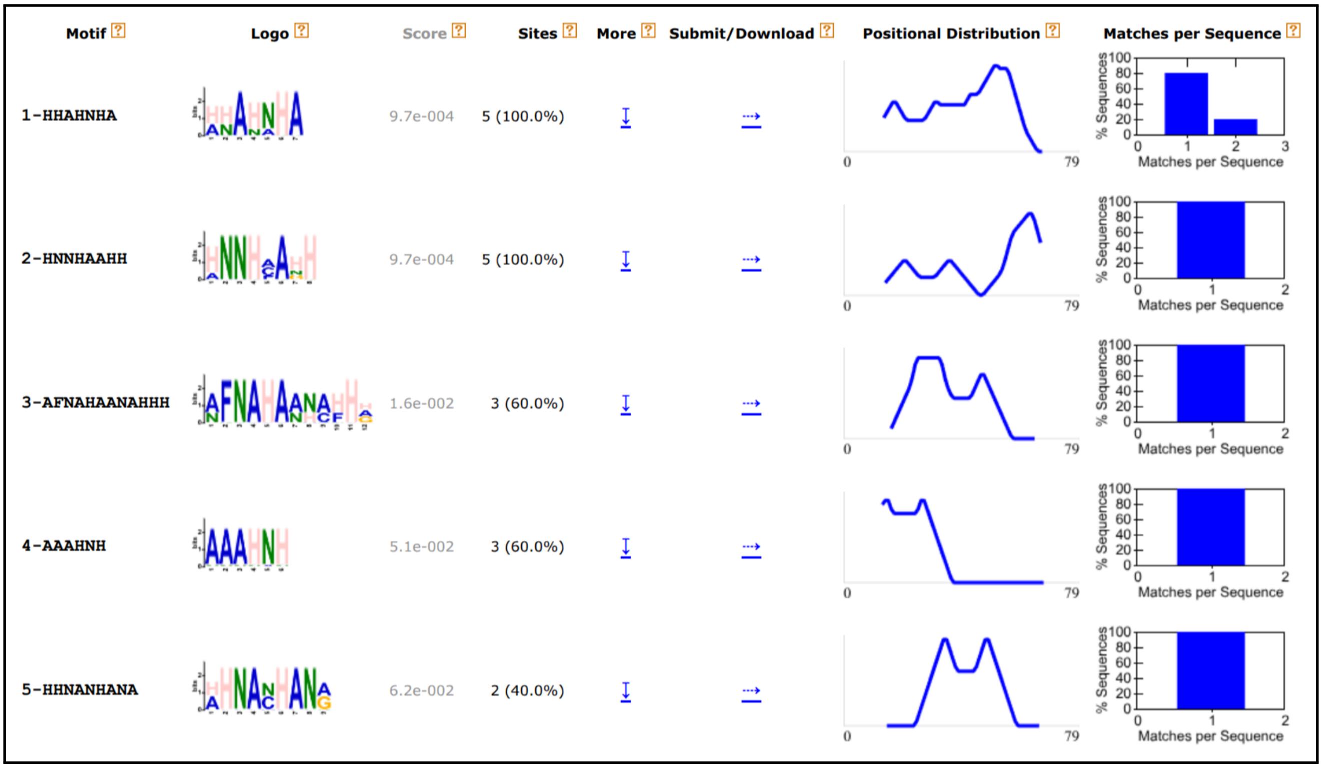

Figure S34 Screen shot from ungapped motif discovery using Streme (1) and amino acid categories of Table S10 for the passenger protein Atp9p.

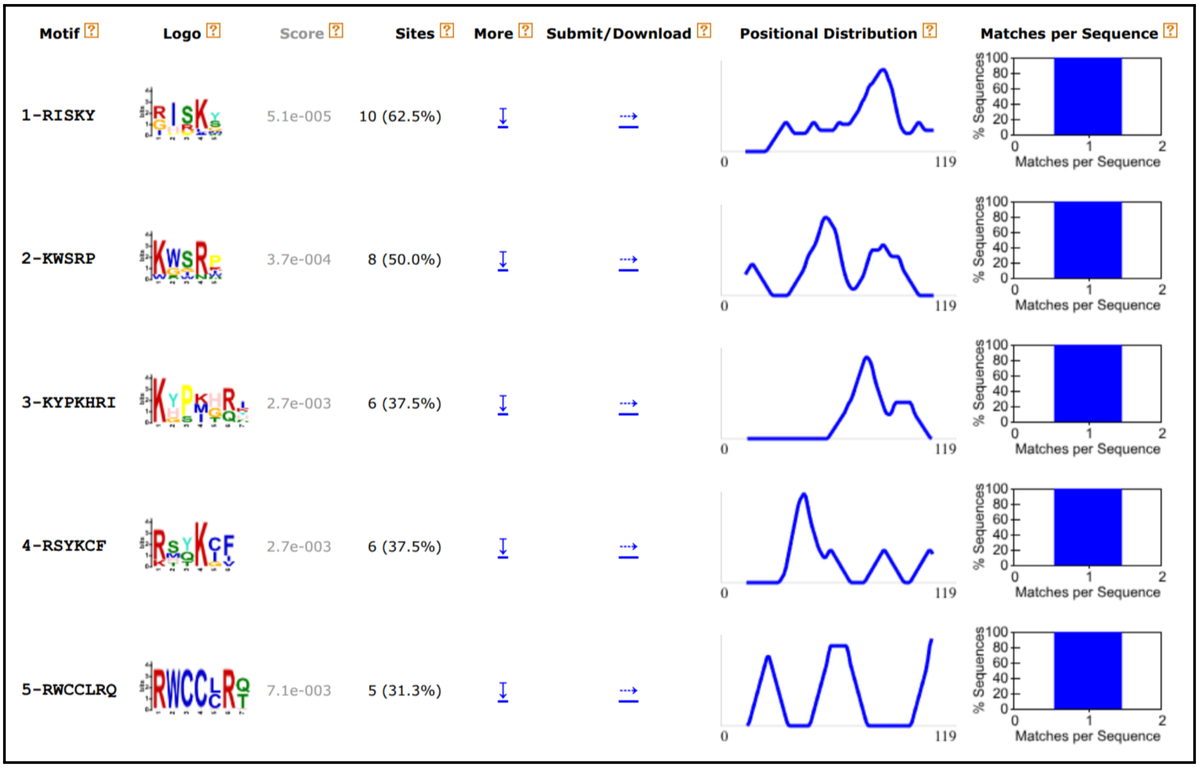

Figure S35 Screen shot from ungapped motif discovery using Streme (1) and the usual amino acids for the passenger protein Atp8p.

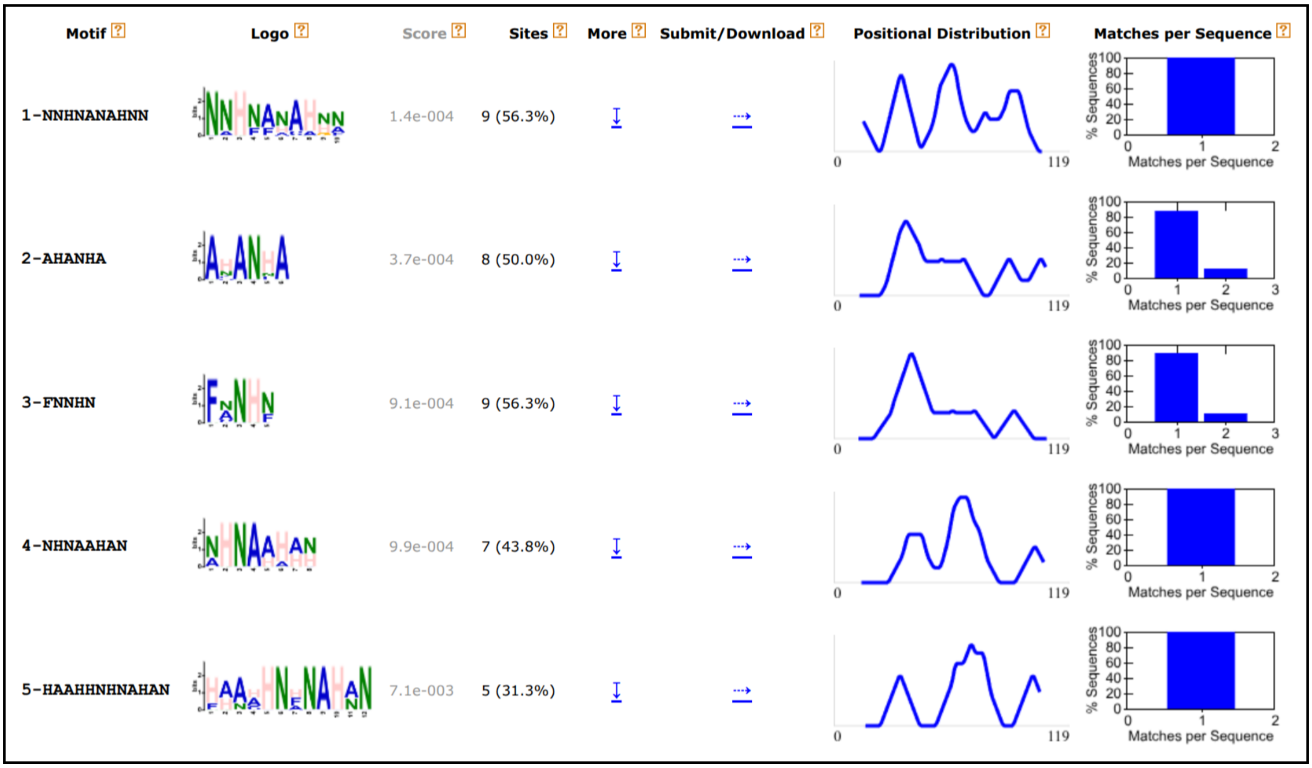

Figure S36 Screen shot from ungapped motif discovery using Streme (1) and amino acid categories of Table S10 for the passenger protein Atp8p.

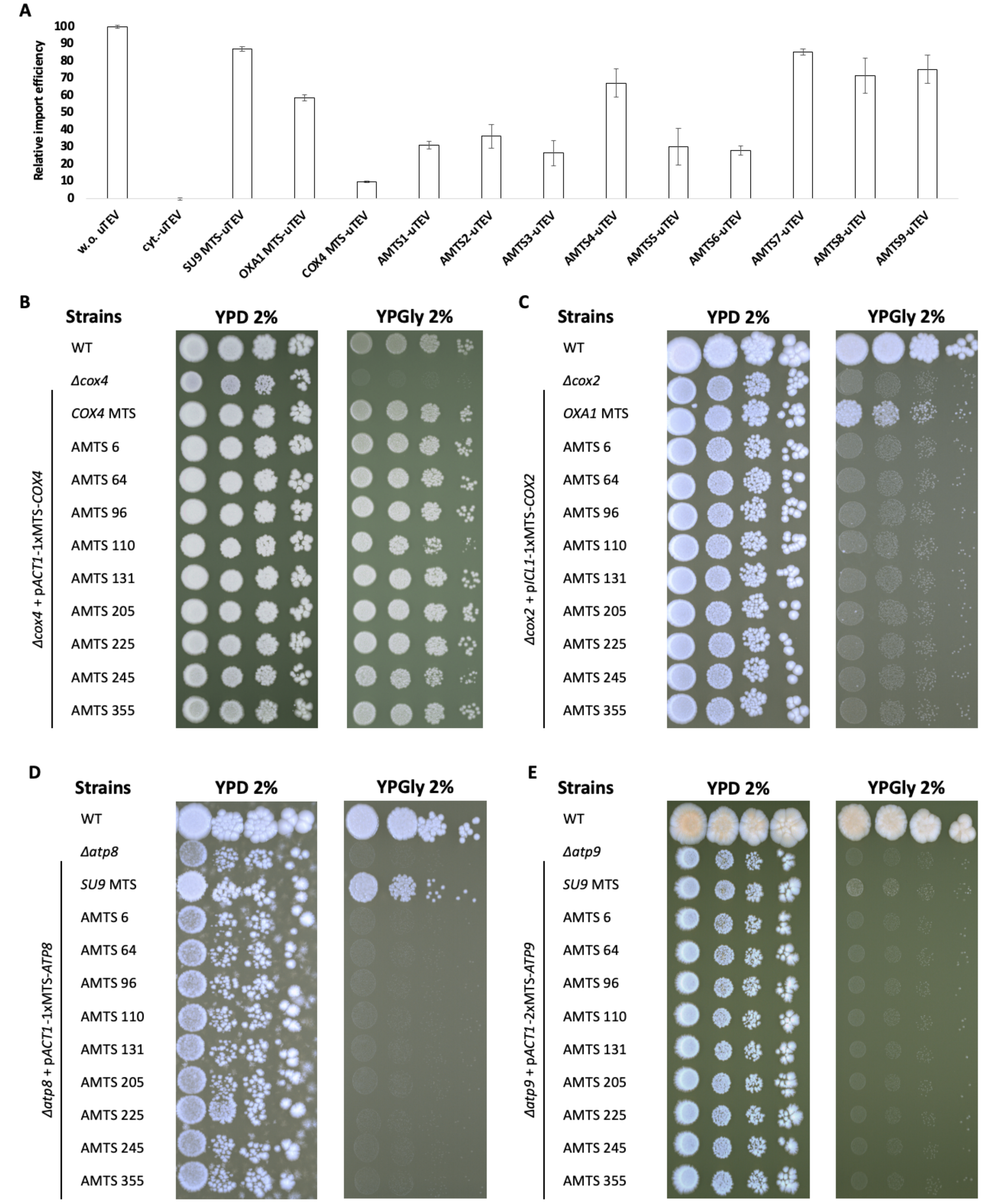

Figure S37 Analysis of the import properties of the artificial mitochondrial targeting sequences (AMTS) (2) using the IQ-Compete assay (A). Evaluation of the restoration of the respiratory growth of the *COX4* (B, 3 days incubation), *COX2* (C, 6 days incubation), *ATP8* (D, 10 days incubation) and *ATP9* (E, 10 days incubation) deletion strains when using the nine different AMTS. The pictures were taken after the indicated incubation period at 30°C.

References

1. T. L. Bailey. Streme: accurate and versatile sequence motif discovery. *Bioinformatics*, 37(18):2834–2840, 2021.
2. A. G. Boob, S.-I. Tan, A. Zaidi, N. Singh, et al. Design of diverse, functional mitochondrial targeting sequences across eukaryotic organisms using variational autoencoder. *Nature Communications*, 16(1):4151, May 2025.
3. G. E. Crooks, G. Hon, J.-M. Chandonia, and S. E. Brenner. Weblogo: A sequence logo generator. *Genome Research*, 14(6):1188–1190, 2004.
4. J. E. Hopcroft, R. Motwani, and J. D. Ullman. *Introduction to Automata Theory, Languages, and Computation*. Pearson Education, Inc., third edition, 2007.
5. T. P. Hopp and K. R. Woods. Prediction of protein antigenic determinants from amino acid sequences. *Proceedings of the National Academy of Sciences*, 78(6):3824–3828, June 1981.
6. S. Nashed, H. E. Barbry, M. Benchouaia, A. Dijoux-Maréchal, et al. Functional mapping of n-terminal residues in the yeast proteome uncovers novel determinants for mitochondrial protein import. *PLoS Genetics*, 19(8):e1010848, 2023.
7. D. L. Nelson and M. M. Cox. *Lehninger Principles of Biochemistry*. W. H. Freeman and Company, fifth edition, 2008.
